# Synthesis and biological evaluation of 3-arylcoumarin derivatives as potential anti-diabetic agents

**DOI:** 10.1080/14756366.2018.1518958

**Published:** 2018-10-26

**Authors:** Yuheng Hu, Bing Wang, Jie Yang, Teng Liu, Jie Sun, Xiaojing Wang

**Affiliations:** a School of Medicine and Life Sciences, University of Jinan-Shandong Academy of Medical Sciences, Jinan, Shandong, China;; b Institute of MateriaMedica, Shandong Academy of Medical Sciences, Jinan, Shandong, China;; c Key Laboratory for Biotech-Drugs Ministry of Health, Jinan, Shandong, China;; d Key Laboratory for Rare & Uncommon Diseases of Shandong Province, Jinan, Shandong, China

**Keywords:** 3-Arylcoumarin, α-glucosidase inhibitor, antidiabetic, AGEs inhibitor

## Abstract

A variety of substituted 3-arylcoumarin derivatives were synthesised through microwave radiation heating. The method has characteristics of environmental friendliness, economy, simple separation, and purification process, less by-products and high reaction yield. Those 3-arylcoumarin derivatives were screened for antioxidant, α-glucosidase inhibitory and advanced glycation end-products (AGEs) formation inhibitory. Most compounds exhibited significant antioxidant and AGEs formation inhibitory activities. Anti-diabetic activity studies showed that compounds **11** and **17** were equipotent to the standard drug glibenclamide *in vivo*. According to the experimental results, the target compound **35** can be used as a lead compound for the development of new anti-diabetic drugs. The whole experiment showed that anti-diabetic activity is prevalent in 3-arylcoumarins, which added a new natural skeleton to the development of anti-diabetic active drugs.

## 1. Introduction

Arylcoumarin is a class of naturally-occurring compounds, the basic skeleton of which is arylbenzopyranones. It mainly includes 3-arylcoumarin and 4-arylcoumarin. Arylcoumarin was discovered in abundance existence in some plants. Later on, scientists conducted a lot of syntheses and activity experiments on the basis of the natural structure of arylcoumarins by splitting and derivatisation^1–3^. Among them, the research on 3-arylcoumarins received great attention. Studies have shown that arylcoumarins exhibit a variety of activities, such as anti-cancer^4^, anti-leukemia^5^, anticoagulant, anti-allergic^6^, anti-inflammatory^7^, antioxidant^8^, antimicrobial^9–11^, estrogen receptor affinity^12^, adenosine receptor affinity^7^, and monoamine oxidase B inhibition activity^13^^,^^14^. Rich pharmacological activity has stimulated interest in the research of arylcoumarins. These compounds have similarities in chemical structure with coumarins, flavones, isoflavones, and *trans*-stilbenes and they are related to each other in the process of biosynthesis in plants^15^. Therefore, they are highly regarded by researchers. Isoflavones, the most abundant phytoestrogens in soy beans, are structurally similar to 3-arylcoumarin derivatives. The compounds have been found effective in the management of diabetes by acting on peroxisome proliferator-activated receptors^16^. Researchers found that resveratrol appears a good candidate as a neutraceutical support for the therapy of obesity and type II diabetes^17^^,^^18^. In recent years, coumarins and flavonoids have received wide attention owing to their excellent advanced glycation end products (AGEs) inhibitory and anti-inflammatory activities^19^^,^^20^. Minpei Kuroda et al.^21^ reported that 3-arylcoumarins have the effect of significantly decreasing the blood glucose levels of genetically diabetic mice. Antioxidant compounds, its free radical scavenger properties imply a protective effect against oxidative stress in the early stages of diabetic nephropathy, leading to symptom relief and improvement in organ function. Antioxidant compounds in reducing diabetes-related neurotoxicity by the restoration of the nervous fiber myelination with the normalisation of pain behavior^22^. These antioxidants fully protected astrocytes from the caspase 3 apoptotic signaling activation induced by toxic substance and it is well known that glia has a pivotal role in neuropathy development and astrocytes may be involved in the maintenance of neuropathic pain^23^^,^^24^.

**Scheme 1.**
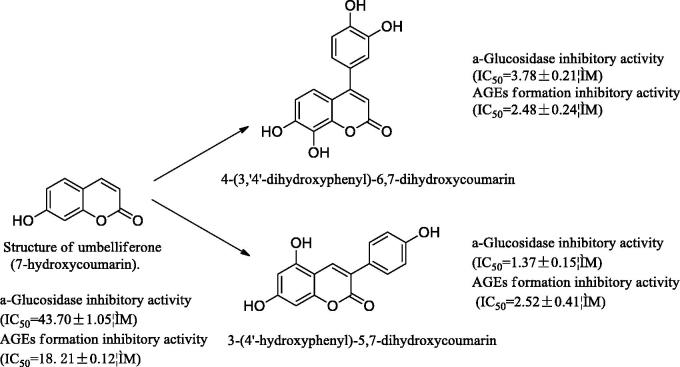
Umbelliferone is derivatised as 3-arylcoumarin and 4-arylcoumarin. They have inhibitory activity on α-glucosidase inhibitory activity and AGEs formation inhibitory activity.

Umbelliferone (UMB), a derivative of coumarin, is a benzopyrone in nature, and it is present in the fruits of bitter orange (*Citrus aurantium*)^25^. It has been reported that UMB has a certain hypoglycemic activity^26^. Coumarin may be a prodrug, and 7-hydroxycoumarin is the pharmacologically active agent^27^. UMB is derivatised as 3-arylcoumarin and 4-arylcoumarin (Scheme 1). Here we focus on the synthesis of 3-arylcoumarin and its pharmacological activity. This work has designed and synthesised a series of compounds, and carried out a series of active screening around the research of anti-diabetes.

## 2. Results and discussion

### 2.1. Chemistry

**Scheme 2.**
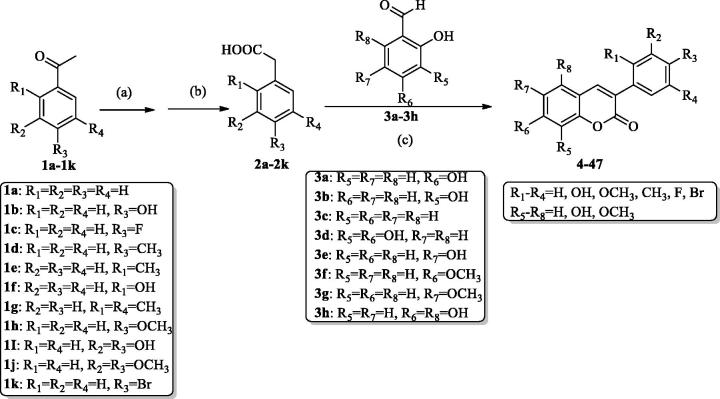
General synthetic route to 3-arylcoumarin derivatives. (a) sulfur, *p*-toluene sulfonic acid, morpholine, 120 °C; (b) NaOH (aq), Tetrabutylammonium bromide, 100 °C; (c) Acetic anhydride, Et_3_N, 112 °C; HCl.

The common synthetic strategies for the target compounds 3-arylcoumarin derivatives are summarised in (Scheme 2). Substituted phenylacetic acid **2a**–**2k** were synthesised with acetophenone derivatives **1a**–**1k**. The substituted phenylacetic acid and substituted salicylaldehyde **3a**–**3h** were used as starting materials to obtain the corresponding compounds **4**–**47** by Perkin reaction. Details on the chemical and spectroscopic characterizations of compounds 4-47 were described in the Supporting Information. In order to confirm the optimal reaction conditions, 4-hydroxyphenylacetic acid **2b** with 2,4-dihydroxybenzaldehyde **3a** were chosen as model substrates. This paper screened different microwave power and reaction time. It was found that this reaction had the highest yield of 96% could be achieved at microwave power of 100 W reaction time of 70 min (Table 1). The previous reaction by heating in an oil bath required a reaction for 6 h, and now the reaction time was shortened to 70 min by microwave heating. Some of the crude product can be obtained by a recrystallisation purification method with a high yield (Table 2). The results showed that microwave heating method had such advantages as low cost, low-toxicity, and good commercial availability, rendering the synthetic process more environmental friendly and economical.

**Table 1.** The reaction conditions and yield.

Yield (%)		Reaction time (min)				
		50	60	70	80	90
Microwave power (W)	60	80	85	86	87	89
	70	83	87	89	90	86
	80	77	79	86	96	86
	90	88	93	93	95	93
	100	85	95	96	93	91

**Table 2.** Compounds **4**–**47**.

Product	R_1_	R_2_	R_3_	R_4_	R_5_	R_6_	R_7_	R_8_	Yield (%)
**4**	H	H	H	H	H	OH	H	H	78.15
**5**	H	H	H	H	OH	H	H	H	54.2
**6**	H	H	OH	H	H	H	H	H	35.29
**7**	H	H	F	H	H	H	H	H	49.58
**8**	H	H	CH_3_	H	OH	H	H	H	55.55
**9**	H	H	CH_3_	H	H	OH	H	H	39.29
**10**	CH_3_	H	H	H	OH	H	H	H	37.22
**11**	CH_3_	H	H	H	H	OH	H	H	41.21
**12**	H	H	OH	H	H	OH	H	H	95.28
**13**	H	H	H	H	H	OH	H	OH	57.09
**14**	H	H	OH	H	OH	H	H	H	51.18
**15**	H	H	H	H	OH	OH	H	H	39.37
**16**	OH	H	H	H	H	OH	H	H	44.88
**17**	H	H	OH	H	H	H	OH	H	73.23
**18**	H	H	F	H	H	OH	H	H	44.92
**19**	H	H	F	H	OH	H	H	H	51.97
**20**	CH_3_	H	H	CH_3_	H	OH	H	H	52.26
**21**	CH_3_	H	H	CH_3_	OH	H	H	H	44.74
**22**	H	H	OCH_3_	H	OH	H	H	H	40.67
**23**	H	H	CH_3_	H	OH	OH	H	H	43.28
**24**	H	H	OCH_3_	H	H	OH	H	H	45.15
**25**	H	H	CH_3_	H	H	OH	H	OH	46.56
**26**	H	H	OH	H	H	OCH_3_	H	H	42.16
**27**	H	H	OH	H	H	H	OCH_3_	H	43.66
**28**	H	H	OH	H	H	OH	H	OH	29.26
**29**	H	OH	OH	H	H	OH	H	H	22.22
**30**	H	H	OH	H	OH	OH	H	H	66.66
**31**	H	H	F	H	H	OH	H	OH	45.82
**32**	H	H	F	H	OH	OH	H	H	56.62
**33**	CH_3_	H	H	CH_3_	OH	OH	H	H	45.04
**34**	H	H	OCH_3_	H	OH	OH	H	H	48.94
**35**	H	H	OCH_3_	H	H	OH	H	OH	54.25
**36**	H	OH	OH	H	OH	OH	H	H	55.59
**37**	H	OCH_3_	OCH_3_	H	H	OH	H	H	72.5
**38**	H	OCH_3_	OCH_3_	H	OH	H	H	H	32.21
**39**	H	OCH_3_	OCH_3_	H	H	H	OH	H	69.82
**40**	H	H	Br	H	H	H	H	H	53.32
**41**	H	H	OH	H	H	Et_2_N	H	H	26.51
**42**	H	OCH_3_	OCH_3_	H	H	OH	H	OH	96.18
**43**	H	OCH_3_	OCH_3_	H	OH	OH	H	H	50.64
**44**	H	H	Br	H	H	OH	H	H	56.43
**45**	H	H	Br	H	OH	H	H	H	55.52
**46**	H	H	Br	H	H	OH	H	OH	52.15
**47**	H	H	Br	H	OH	OH	H	H	54.65

**Scheme 3.**
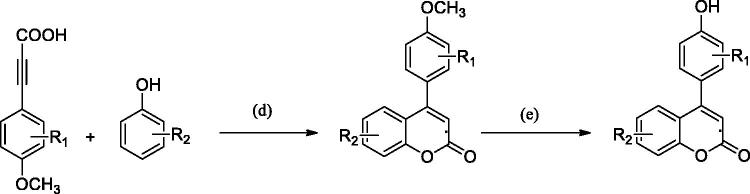
The main synthesis step of 4-arylcoumarin derivatives. (d) montmorillonite K-10, nitrobenzene, 100 °C; (e) I_2_, Al, acetonitrile.

Our group has previously synthesised a series of 4-arylcoumarins (Scheme 3)^2^. 4-Arylcoumarin is more difficult to synthesise than 3-arylcoumarin and has more reaction steps. The synthesis of 4-arylcoumarin has certain guiding significance for the synthesis of 3-arylcoumarin. In the following experiments and discussion, we will compare the *in vitro* activities of 3-arylcoumarin and 4-arylcoumarin with the same substituents sites.

### 2.2. Biological evaluation

#### 2.2.1. *In vitro* antioxidant activity

All the synthesised compounds were evaluated for their antioxidant activities in the way of scavenging 2,2-diphenyl-1-picrylhydrazyl (DPPH)^28^ and hydroxyl radical^29^. Vitamin C was used as a reference compound in this assay. As shown in Table 3, most compounds of **4–47** demonstrated moderate to great scavenging activity, among which compound **36** with several hydroxyl groups showed an excellent activity in both aspects.

**Table 3.** Biological evaluation *In vitro.*

Product	DPPH	OH	α-Glucosidaseinhibitory activity	AGEs inhibitory activity
	IC_50_ Value (μM)			
**4**	76.85 ± 1.30	>1000	25.67 ± 0.50	21.81 ± 3.07
**5**	>1000	>1000	280.38 ± 74.79	48.36 ± 3.53
**6**	>1000	>1000	60.88 ± 0.55	>1000
**7**	>1000	>1000	>1000	>1000
**8**	>1000	>1000	69.60 ± 5.32	31.86 ± 2.02
**9**	60.20 ± 1.27	>1000	118.29 ± 41.47	>1000
**10**	>1000	>1000	>1000	>1000
**11**	564.21 ± 5.20	>1000	16.39 ± 1.23	>1000
**12**	756.42 ± 8.31	>1000	19.91 ± 3.39	1.42 ± 0.04
**13**	268.98 ± 0.04	>1000	13.46 ± 0.71	14.92 ± 0.20
**14**	167.05 ± 2.01	>1000	212.72 ± 40.35	45.24 ± 5.28
**15**	2.01 ± 0.08	996.24 ± 8.03	29.05 ± 2.87	47.60 ± 1.93
**16**	>1000	>1000	10.16 ± 0.75	20.47 ± 0.20
**17**	163.23 ± 7.13	>1000	11.54 ± 1.30	24.68 ± 0.12
**18**	>1000	>1000	86.91 ± 1.17	20.59 ± 0.39
**19**	39.14 ± 3.83	>1000	>1000	34.73 ± 1.84
**20**	>1000	>1000	>1000	>1000
**21**	>1000	>1000	>1000	212.97 ± 4.77
**22**	15.63 ± 0.11	>1000	>1000	23.06 ± 0.82
**23**	3.21 ± 0.04	800.82 ± 41.83	25.48 ± 0.52	75.93 ± 10.75
**24**	53.92 ± 1.83	>1000	27.42 ± 0.11	>1000
**25**	603.32 ± 7.42	>1000	11.49 ± 0.37	45.75 ± 1.27
**26**	68.69 ± 13.36	>1000	>1000	65.89 ± 1.34
**27**	42.01 ± 0.97	>1000	70.26 ± 2.95	64.55 ± 0.78
**28**	41.51 ± 0.18	>1000	1.37 ± 0.15	2.52 ± 0.41
**29**	11.85 ± 0.04	981.55 ± 30.04	29.89 ± 5.15	7.48 ± 0.59
**30**	2.18 ± 0.04	696.52 ± 19.59	19.04 ± 1.11	>1000
**31**	595.18 ± 11.29	>1000	19.08 ± 0.26	3.12 ± 0.33
**32**	2.06 ± 0.07	969.74 ± 70.85	>1000	33.49 ± 1.99
**33**	2.98 ± 0.03	903.16 ± 41.88	>1000	2.91 ± 0.11
**34**	3.66 ± 0.03	>1000	18.80 ± 1.02	>1000
**35**	154.61 ± 10.49	270.14 ± 41.55	10.81 ± 0.63	5.21 ± 0.07
**36**	1.33 ± 0.03	244.02 ± 11.78	247.34 ± 36.22	14.16 ± 1.33
**37**	173.42 ± 42.58	>1000	>1000	4.23 ± 0.23
**38**	16.28 ± 0.44	>1000	20.23 ± 0.44	22.28 ± 1.34
**39**	>1000	>1000	13.09 ± 0.57	8.02 ± 0.20
**40**	>1000	>1000	>1000	12.99 ± 0.40
**41**	>1000	>1000	13.43 ± 0.65	>1000
**42**	5.89 ± 0.06	>1000	39.08 ± 0.76	18.31 ± 0.38
**43**	4.24 ± 0.06	886.85 ± 15.82	>1000	25.22 ± 1.72
**44**	>1000	>1000	>1000	7.51 ± 0.38
**45**	487.82 ± 95.05	595.87 ± 28.77	>1000	>1000
**46**	>1000	>1000	>1000	47.75 ± 0.15
**47**	2.37 ± 0.06	688.44 ± 49.70	35.71 ± 0.48	298.83 ± 40.15
**Vitaminc**	19.60 ± 1.88	787.50 ± 15.74		
**Acarbose**			0.050 ± 0.003	
**AG**				269.87 ± 7.19

Each value represents the mean ± SD (*n* = 3).

DPPH is widely used to evaluate antioxidant capacities of natural and synthetic products. Although the main skeleton of all compounds is same, the slight difference in their inhibitory potential might result from the different substitution patterns on benzene ring. As shown in Table 3, the number and position of hydroxy groups were correlated with DPPH radical scavenging activity. Generally, the increase of hydroxy groups and the decrease of methoxygroups were positive to radical scavenging capacity. Moreover, compounds with adjacent 7,8-dihydroxy groups or 3′,4′-dihydroxy groups possessed a great capacity to scavenge DPPH radical, especially compounds **15**, **23**, **30**, **32**–**34**, **36**, and **37**, which were better than positive reference substance Vitamin C (IC_50_ = 34.94 ± 0.85 µM). These conclusions were similar to reference [30].

In addition, it can be found from the data that almost all compounds with two adjacent hydroxy groups exhibit hydroxyl radical scavenging activity. Especially, the IC_50_ values of compounds **35** and **36** were 245.74 ± 11.87 and 268.68 ± 41.26 µM, respectively, which were threefolds of the value of Vitamin C (IC_50_ = 787.50 ± 15.74 µM).

#### 2.2.2. In vitro α-glucosidase inhibitory activity^31^

a-Glucosidases, enzymes anchored in the brush border of the small intestine, are responsible for catalyzing the hydrolysis of carbohydrates^32^. Their inhibitors were useful for the treatment of type II diabetes mellitus^33^. Though all the compounds were tested for α-glucosidase inhibitory activity *in vitro*, as shown in Table 3, only eight compounds (**11**, **13**, **16**, **17**, **25**, **28**, **35**, and **39**) presented moderate to excellent inhibitory activity for a-glucosidase. Notably, **28** (IC_50_ = 1.37 ± 0.67 µM) had relatively strong activity, which was a little weaker than acarbose (IC_50_ = 0.05 ± 0.003 µM). The 3-arylcoumarin with 7-hydroxyl group showed good inhibitory activity, indicating that 7-hydroxyl group was very important for inhibiting a-glucosidase. Comparison of the IC_50_ values, **4** > **13** and **12** > **28**, showed that 3-arylcoumarin with 7-hydroxyl group was weaker than that with 5,7-dihydroxy groups in meta-position. Comparison of the IC_50_ values, **12** > **17** > **28**, showed that 3-arylcoumarin with 7-hydroxyl group was weaker than that with 6-hydroxyl group. However, the 3-arylcoumarin with 6-hydroxyl group was weaker than that of 3-arylcoumarin with 5,7-dihydroxy groups in meta-positi on in α-glucosidase inhibitory activity. Comparison of the IC_50_ values, **13** > **25** > **35** > **28**, showed that 3-arylcoumarin with 4′-hydroxy was stronger than that with 4′-methoxy and 4′-methyl in α-glucosidase inhibitory activity and the inhibitory activity of 4′-methyl and 4′-methoxy group was similar but stronger than that of hydrogen. After compound **35** was converted to **28** via demethylation, its IC_50_ value decreased from 10.81 ± 0.63 µM to 1.37 ± 0.67 µM. This suggests that the 4′-hydroxyl group is another active site in α-glucosidase inhibitory activity. According to *in vitro* α-glucosidase inhibition test results, we decided to study *in vivo* hypoglycemic activity of compounds**11**, **17**, **25**, **28**, and **35**.

#### 2.2.3. *In vitro* inhibitory activity of AGEs formation^34^

AGEs are a group of complex and heterogeneous compounds, which are implicated in a number of biochemical abnormalities associated with diabetes^35^. Therefore, the discovery of AGEs inhibitory activity would be beneficial to the prevention and treatment of diabetic or other pathogenic complications. The results listed in Table 3 displayed that most of the target compounds presented better inhibitory activity to AGEs, even better than amino guanidine (AG) (IC_50_ = 269.87 ± 7.19 µM). The IC_50_ values of compounds **12**, **28**, **33**, **31**, **37**, **39**, and **44** were less than 10.00 µM, and the inhibitory effect was over 10 times compared with the positive control. When the 3-arylcoumarin both have the 4′-hydroxy groups, those compounds with 7-hydroxy group were better than those with 5,7-dihydroxy groups and which were stronger than those with 6-hydroxy group. The 4′-fluoro group had substantially no effect on the activity compared with the 4′-hydroxy group, but 4′-bromo group decreased the inhibitory activity. Comparison of the IC_50_ values, **35 **>** 28**, showed that 3-arylcoumarin with 4′-hydroxy is stronger than that with 4′-methoxy and 4′-methyl in AGEs formation inhibitory activity.

Our research group has conducted in-depth studies on the synthesis and hypoglycemic activity of 4-arylcoumarin compounds. Those 4-arylcoumarin compounds were screened for antioxidant, α-glucosidase inhibitory, aldose reductase 2 inhibitory and AGE formation inhibitory effects^2^. The α-glucosidase inhibitory activity and AGEs formation inhibitory activity of 3-arylcoumarin and 4-arylcoumarin substituted at the same position (Table 4). In summary, 3-arylcoumarin substituted at the same position is superior to 4-arylcoumarin in terms of α-glucosidase inhibitory activity and AGEs formation inhibitory activity. Therefore, we have studied the synthesis and preliminary activity of 3-arylcoumarin with good practical significance.

**Table 4.** Comparison of *in vitro* activity of 3-arylcoumarin and 4-arylcoumarin.

a: 3-arylcoumarin; b: 4-arylcoumarin; G: α-glucosidase inhibitory activity; A: AGEs formation inhibitory activity.

The 3-arylcoumarin derivatives are structurally similar to the *trans*-stilbene and isoflavone compounds. Therefore, we have selected synthetic compounds **28** and resveratrol, a representative compound of *trans*-stilbene, genistein a representative of isoflavone compound, to compare α-glucosidase inhibitory activity and AGEs formation inhibitory activity (Table 5). The number and location of the hydroxyl groups of these three compounds were basically the same. We found that compound **28** we synthesised is superior to the other two compounds in both α-glucosidase inhibitory activity and AGEs formation inhibitory activity. It can be seen that the closed pyrone ring can enhance α-glucosidase inhibition and AGEs formation inhibitory activity. The 3-arylcoumarin compound having a *trans*-stilbene structure is superior to the *trans*-stilbene structured isoflavone.

**Table 5.** Compound **28** resveratrol and genistein have a comparative effect on α-glucosidase inhibitory activity and AGEs formation inhibitory activity.

#### 2.2.4. Oral toxicity to mice

With reference to Lorke’s method^36^, kunming mice were used as targets to estimate the oral toxicity of each compound to mice. We select compounds **11**, **17**, **25**, **28**, and **35** at concentrations of 10, 100, and 1000 mg/kg to test their oral toxicity in the first phase, and 1600, 2900, and 5000 mg/kg at the second phase. Results showed that none of the tested compounds significantly affected mice’ viability. No death and no appetite-suppressant effect were detected in the tested mice in 14 days. Since no death or damage was observed throughout the experiment, the LD_50_ was higher than 5000 mg/kg for the three compounds assayed, indicating their innocuousness for mice.

#### 2.2.5. Acute hypoglycemic assay

The anti-diabetic activity was determined by using a standard method^31^. As shown in Table 6, the target compounds **11**, **17**, **25**, **28**, and **35** (10 mg/kg, 30 mg/kg, and 100 mg/kg of body weight [bw]) caused decreases in blood glucose levels in streptozotocin (STZ)-diabetic mice compared with normal mice (Table 7). Especially, **35** (30 mg/kg of bw) caused significant decreases in blood glucose levels when compared with vehicle-treated groups (*p* < 0.05). The target compound **35** (10 mg/kg of bw) caused significant decreases in blood glucose levels even better than positive control at 5, 7, and 9 h. In STZ-diabetic animals, the hypoglycemic effect of **17** and **35** (30 mg/kg of bw) was larger than 50% started from 3 h and persisted throughout the experiment (Table 6). The highest anti-hyperglycemic effect of **35** was observed at doses of 10 and 30 mg/kg at 7 h (∼73.66%) and 9 h (∼74.54%), respectively (Table 6). In diabetic animals, the highest anti-hyperglycemic effect of **17** was observed at doses of 30 and 100 mg/kg at 9 h (∼70.39%) and 7 h (∼70.51%), respectively (Table 6). The target compounds **11** and **17** showed similar anti-diabetic activity throughout the experiment. Glibenclamide (10 mg/kg of bw), used as a positive control, showed maximum hypoglycemic effect at 9 h in STZ-induced diabetic mice (Table 6). The administration of the target compounds **11**, **17**, and **35** (10 mg/kg, 30 mg/kg, and 100 mg/kg of bw) showed significant decreases of blood glucose levels in normoglycemic mice (Table 7).

**Table 6.** Acute effect of compounds **11**, **17**, **25**, **28** and **35** on blood glucose levels in STZ-diabetics mice.

Test samples	Blood glucose concentration(mM)						
	Dose(*per os*) mg/kg of bw	0h	1.5h	3h	5h	7h	9h
Control (vehicle)	–	24.7 ± 5.8	22.7 ± 5.4	15.9 ± 4.9	15.4 ± 6.5	14.9 ± 7.1	15.8 ± 5.3
			(−8.23)	(−35.63)	(−37.79)	(−39.88)	(−35.97)
Glibenclamide	10	16.9 ± 4.3	7.2 ± 4.0*	6.9 ± 1.6	6.7 ± 2.5	6.5 ± 2.2	4.9 ± 2.5**
			(−55.28)	(−57.14)	(−55.90)	(−59.63)	(−70.81)
**11**	10	24.9 ± 4.1	20.6 ± 5.9	15.5 ± 8.5	12.8 ± 10.4	11.4 ± 8.2	11.9 ± 8.5
			(−17.27)	(−37.75)	(−48.59)	(−54.22)	(−52.21)
**11**	30	25.3 ± 6.3	20.0 ± 6.3	16.5 ± 6.4	11.3 ± 4.0	9.5 ± 3.9	8.1 ± 4.2
			(−20.95)	(−34.78)	(−55.41)	(−62.40)	(−68.14)
**11**	100	23.5 ± 5.4	18.3 ± 3.9	15.3 ± 3.9	11.1 ± 4.4	8.9 ± 2.8	8.2 ± 2.6
			(−21.88)	(−34.89)	(−52.76)	(−62.21)	(−65.27)
**17**	10	26.9 ± 3.8	24.5 ± 3.0	22.8 ± 2.3	16.6 ± 5.8	14.8 ± 8.0	14.7 ± 9.5
			(−8.92)	(−15.24)	(−38.29)	(−44.98)	(−45.35)
**17**	30	23.3 ± 3.2	17.9 ± 5.7	11.6 ± 4.8	8.3 ± 3.8	7.4 ± 6.7	6.9 ± 7.9*
			(−23.18)	(−50.22)	(−64.38)	(−68.24)	(−70.39)
**17**	100	23.4 ± 5.7	18.8 ± 6.0	12.5 ± 4.7	7.6 ± 3.8	6.9 ± 2.6	8.0 ± 2.3
			(−19.66)	(−46.58)	(−67.52)	(−70.51)	(−65.81)
**25**	10	19.7 ± 6.1	15.7 ± 6.6	14.9 ± 6.5	9.7 ± 3.4	6.9 ± 3.9	8.5 ± 2.2
			(−20.30)	(−24.37)	(−50.76)	(−64.97)	(−56.85)
**25**	30	24.8 ± 4.9	21.7 ± 3.9	15.8 ± 4.5	8.9 ± 6.3	9.0 ± 4.8	8.9 ± 3.8
			(−12.50)	(−36.23)	(−64.11)	(−63.71)	(−64.11)
25	100	23.0 ± 4.8	18.3 ± 3.5	17.2 ± 2.9	9.9 ± 2.7	8.1 ± 1.6	9.1 ± 2.3
			(−20.44)	(−25.36)	(−56.95)	(−64.78)	(−60.43)
**28**	10	15.3 ± 3.8	14.8 ± 5.8	11.4 ± 4.2	8.9 ± 2.6	6.4 ± 1.6	7.0 ± 2.4
			(−3.27)	(−25.49)	(−41.83)	(−58.17)	(−54.25)
**28**	30	18.2 ± 6.7	14.9 ± 6.6	13.5 ± 6.1	12.7 ± 6.7	10.3 ± 5.6	7.3 ± 4.5
			(−18.13)	(−25.82)	(−30.22)	(−43.41)	(−59.89)
**28**	100	18.2 ± 4.1	17.3 ± 6.7	13.0 ± 6.6	11.0 ± 6.4	11.8 ± 6.0	10.2 ± 4.6
			(−4.95)	(−28.57)	(−39.56)	(−35.17)	(−43.96)
**35**	10	20.5 ± 7.1	13.1 ± 6.4*	10.7 ± 5.7	5.6 ± 3.1*	5.4 ± 3.4*	5.7 ± 3.1*
			(−36.10)	(−47.81)	(−72.68)	(−73.66)	(−72.20)
**35**	30	21.6 ± 4.4	12.0 ± 3.7*	10.5 ± 5.1	6.3 ± 4.3	5.7 ± 6.7*	5.5 ± 5.2*
			(−44.44)	(−51.39)	(−70.83)	(−73.61)	(−74.54)
**35**	100	20.3 ± 4.4	15.1 ± 5.0	10.5 ± 4.9	7.2 ± 2.6	7.2 ± 3.9	6.4 ± 2.0*
			(−25.62)	(−48.28)	(−64.53)	(−64.53)	(−68.47)

Each value is the mean ± SEM for six mice in each group.

* *p* < 0.05 significantly different ANOVA followed by Dunnett’s *t*-test for comparison with respect to initial levels in each group.

% Variation of glycemia are in parentheses.

**Table 7.** Acute effect of compounds **11**, **17** and **35** on blood glucose levels in normal mice.

Test samples	Blood glucose concentration(mM)						
	Dose (*per os*) mg/kg of bw	0h	1.5h	3h	5h	7h	9h
Control (vehicle)	–	10.9 ± 1.1	9.2 ± 0.3	8.6 ± 0.5	8.3 ± 0.3	7.8 ± 0.5	7.3 ± 0.4
			(−15.60)	(−21.10)	(−24.16)	(−28.75)	(−33.33)
Glibenclamide	10	9.8 ± 1.5	6.4 ± 1.4*	5.8 ± 0.8**	5.0 ± 1.4**	4.3 ± 0.6**	4.1 ± 0.7**
			(−34.58)	(−41.36)	(−49.15)	(−56.61)	(−58.31)
**11**	10	10.0 ± 0.7	9.1 ± 0.2	7.7 ± 0.6	6.6 ± 0.5	5.6 ± 0.4**	5.5 ± 1.2**
			(−15.27)	(−28.35)	(−38.01)	(−47.98)	(−48.91)
**11**	30	10.2 ± 1.2	8.7 ± 1.2	6.9 ± 1.1	5.9 ± 1.1*	5.2 ± 0.8**	5.1 ± 0.6**
			(−13.62)	(−30.90)	(−40.86)	(−47.84)	(−49.17)
**11**	100	10.4 ± 0.7	7.0 ± 1.1	5.9 ± 0.6*	4.8 ± 1.3**	4.7 ± 0.4**	4.3 ± 0.3**
			(−31.37)	(−42.16)	(−52.94)	(−54.25)	(−58.17)
**17**	10	10.6 ± 0.4	7.9 ± 0.7	6.9 ± 0.8	5.7 ± 0.7**	5.4 ± 0.5**	5.1 ± 0.4**
			(−23.96)	(−34.19)	(−45.37)	(−48.56)	(−51.12)
**17**	30	10.8 ± 0.3	8.4 ± 1.0	6.9 ± 0.9	5.3 ± 0.7**	5.3 ± 1.1**	4.5 ± 0.6**
			(−18.39)	(−33.55)	(−48.71)	(−48.71)	(−56.45)
**17**	100	10.4 ± 0.2	7.5 ± 1.2	6.3 ± 0.9*	5.2 ± 0.5**	4.7 ± 0.4**	4.3 ± 0.4**
			(−25.90)	(−37.71)	(−49.18)	(−53.44)	(−58.03)
**35**	10	10.8 ± 0.6	9.2 ± 0.6	8.2 ± 0.5	8.0 ± 0.4	7.4 ± 0.5	7.1 ± 0.3
			(−14.55)	(−24.15)	(−25.70)	(−31.27)	(−34.06)
**35**	30	10.5 ± 0.9	8.7 ± 1.0	7.2 ± 0.8	6.4 ± 1.0	5.8 ± 0.7**	5.9 ± 0.6
			(−17.72)	(−31.65)	(−39.24)	(−44.94)	(−44.30)
**35**	100	10.6 ± 0.4	8.7 ± 0.5	6.3 ± 1.0*	5.4 ± 0.6*	4.8 ± 0.6**	4.6 ± 0.7**
			(−18.18)	(−40.44)	(−49.22)	(−55.17)	(−57.05)

Each value is the mean ± SEM for six mice in each group.

* *p* < 0.05 significantly different ANOVA followed by Dunnett’s *t*-test for comparison with respect to initial levels in each group.

% Variation of glycemia are in parentheses.

#### 2.2.6. Effects of daily treatment with compounds 11, 17, and 35 in STZ-induced diabetic mice

**Figure 1.**
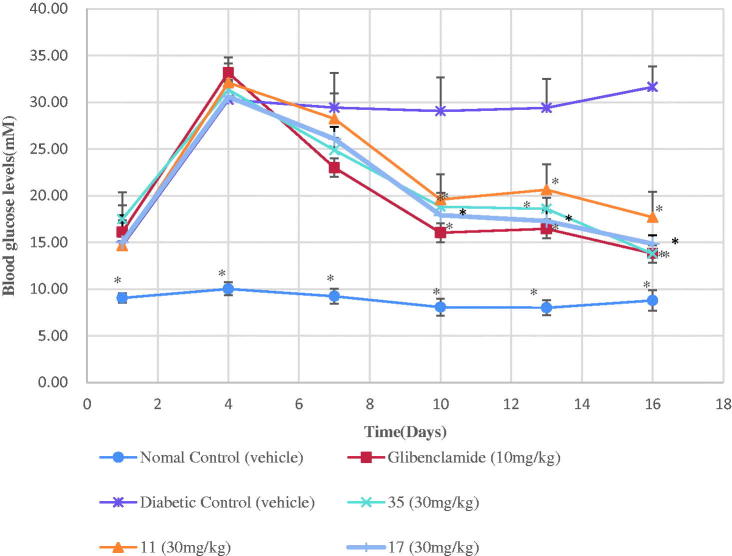
Long-term effect of the compounds **11**, **17** and **35** on blood glucose levels in STZ-diabetic mice. Each value is the mean ± SEM for six mice in each group. *p < 0.05 significantly different ANOVA followed by Dunnett’s *t*-test for comparison with the diabetic control group at same time.

**Figure 2.**
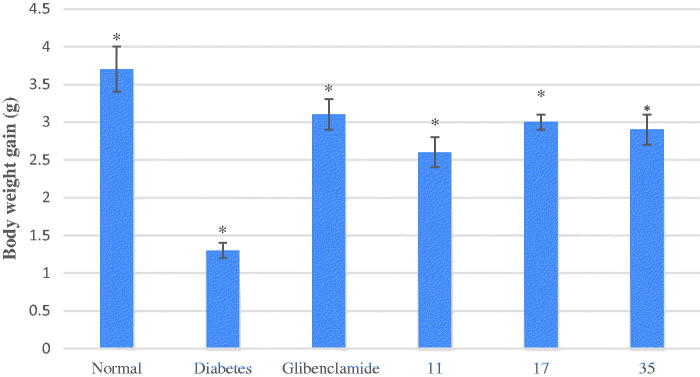
Body weight changes of daily treatment with compounds **11**, **17** and **35** in STZ-induced diabetic mice. Each value is the mean ± SEM for six mice in each group. *p < 0.05 significantly different ANOVA followed by Dunnett’s *t*-test vs. the diabetic control group at same time.

The long-term anti-hyperglycemic effect of the target compounds **11**, **17**, and **35** was tested by using a classical chronic experiment with STZ-induced diabetic mice^37^. The results demonstrated that daily oral administration of the target compounds **17** and **35** (30 mg/kg of bw each time), once a day, for 16 days, induced obviously anti-hyperglycemic effect in the STZ-diabetic mice (Figure 1). The target compound **11** was less efficient in decreasing blood glucose levels in diabetic mice (Figure 1). Compounds **35** restored blood glucose levels to near normal values at the end of the experiment (Figure 1) (16 days) and the effect was equipotent to that of the glibenclamide which was used as a positive control^38^. The treatments with the target compounds **11**, **17**, and **35** also prevented bw loss in hyperglycemic mice (Figure 2) and the effect was equipotent to that of glibenclamide. Furthermore, **17** and **35** were more efficient than **11** in preventing bw loss in hyperglycemic mice (Figure 2).

#### 2.2.7. Oral glucose tolerance test of compounds 11, 17, and 35 on in STZ-induced diabetic mice

**Figure 3.**
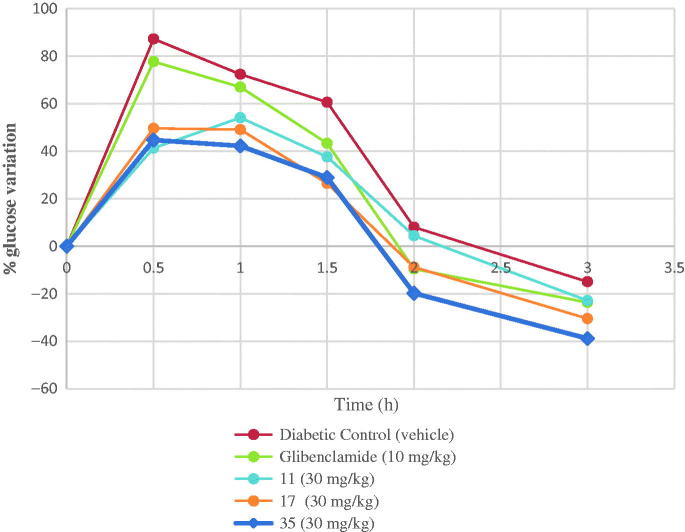
Oral glucose tolerance test of compounds **11**, **17**, and **35** on in STZ-induced diabetic mice. Each value is the mean ± SEM for six mice in each group. *p < 0.05 significantly different ANOVA followed by Dunnett’s *t*-test vs. the diabetic control group at same time.

The oral glucose tolerance test was determined by using a standard method^39^. Compared with vehicle-treated group (*p* < 0.05) (Figure 3), the target compounds **17** and **35** (30 mg/kg of bw) caused significant decrease in the postprandial glycemia peak in both normal (data not shown) and STZ-diabetic mice. The oral glucose tolerance effect of the target compound **35** (30 mg/kg of bw) was stronger than that of the glibenclamide (10 mg/kg of bw) used as a positive control throughout the experiment. Compounds **17** and **35** decreased blood glucose levels to below positive control group the values of the end of the experiment (Figure 3).

## 3. Conclusions

Forty-four 3-arylcoumarin derivatives were synthesised by using microwave reactor heating method. Compared with traditional methods, this method is more environmental friendly, more sustainable and economical, more convenient in isolation and purification processes owing to less byproducts, and the yield is relatively higher than traditional methods. We then studied the pharmacological activity of the synthesised compounds. All the synthesised compounds were evaluated for their antioxidant activities in the way of scavenging DPPH and hydroxyl radical. Most compounds of **4–47** demonstrated moderate to great scavenging activity, among which compound **36** with several hydroxyl groups showed an excellent activity in both aspects.

Compounds **12**, **28**, and **35** showed significant effect on the treatment of diabetic complications. Notably, **17**, **28**, and **35** had relatively strong activity, which displayed a little weaker capacity than acarbose. Oral toxicity tests indicated their innocuousness for mice. We selected compounds **11**, **17**, **25**, **28**, and **35** to investigate their anti-diabetic activity *in vivo*. June Hyuk Jang et al.^40^ reported acarbose as a positive control, the effects of scopoletin on hyperglycemia following a meal were investigated in normal mice and mice with STZ-induced diabetes. Treatment of mice with antioxidants viz., Vitamin C and taurine resulted in only a marginal to moderate protection against STZ-induced elevation in the plasma glucose levels^41^. Jeong-Ho Oak et al.^42^ reported that AG (100 mg/kg/day) treatment had no significant effect on STZ-induced hyperglycemic mice. The results showed the effect of the target compounds **17** and **35** were equipotent to that of the glibenclamide used as a positive control *in vivo*. In conclusion, the target compound **35** offered a potential drug design concept for the development of therapeutic or preventive agents for diabetes and complications of diabetes.

## 4. Experimental

### 4.1. Synthesis

#### 4.1.1. Materials and methods

Melting points were determined using a Thiele tube and were uncorrected. The FT-IR spectra were recorded using a Thermo-Nicolet Nexus 670 spectrometer with KBr pellets. The ^1^HNMR and ^13^CNMR spectra were recorded with a Bruker AM-600 spectrometer (Billercia, MA) with TMS as the internal standard. Chemical shifts were reported at room temperature on a scale (ppm) with DMSO-d6 as the solvents and *J* values are given in Hertz.

Mass spectra were obtained with an Agilent Trap VL LC/MS spectrometer (Santa Clara, CA). The absorbance was recorded by a Hitachi U-3000 UV spectrophotometer (Tokyo, Japan). Columnchromatography was performed on silica gel (200–300 mesh). Unless otherwise noted, all solvents and reagents were commercially available and used without further purification.

#### 4.1.2. General method for synthesis of compounds 2a-k

A solution of sulfur (1.28 g, 40 mmol) and *p*-toluenesulfonic acid (0.1 g, 0.6 mmol) in morpholine (6 ml, 60 mmol). Compound **1a** (20 mmol) was added and the mixture was heated in an oil bath at 120 °C for 8–10 h. Methanol (30 ml) and activated carbon (0.1g) were added and the mixture was heated in an oil bath for 5 min then allowed to cool to room temperature. Ethanol (30 ml, 70%) and NaOH solution (3 ml, 50%) were added to the mixture. After being stirred and heated to reflux for 4 h, the resulting mixture was quenched by the addition of 100 ml water, later the solution was acidified to pH 3–4 with concentrated HCl and precipitated crystals. The precipitate was filtrated and the filter cake was washed with water to give phenylacetic acid **2a** (1.99 g, 73% yield). Compounds **2d–k** were obtained using the same procedures.

#### 4.1.3. General method for the synthesis of 3-arylcoumarin 4–47

Compound **2a** (1.36 g, 10 mmol) and compound 3a (1.54 g, 10 mmol) were added to triethylamine (5.56 g, 55 mmol) and acetic anhydride (6.12 g, 60 mmol) and then the mixture was heated by microwave reactor and refluxed for 70 min. The resulting mixture was quenched by the addition of water, and a large amount of solid was precipitated. The resulting mixture was quenched by the addition of water, and a large amount of solid was precipitated. The filtrate was vacuum filtered and the resulting solid was washed three times with water .The obtained solid was dissolved in 10 ml of absolute ethanol, and then reacted by adding hydrochloric acid (40 ml, 20%) at 80 °C for 3 h (TLC monitoring). The reaction solution was poured into 50 ml of ice water and stirred to precipitate a large amount of light yellow solid. After standing and filtration, the solid was washed with water until the pH of the wash solution was nearly neutral. The solid was recrystallised from ethanol/water to obtain compound **4** (1.86 g, 73% yield). Compounds **5–47** were obtained using the same procedures.

***7-Hydroxy-3-arylcoumarin (4).*** White solid, yield: 78%, m.p. 201.2–203.3 °C, IR (KBr, ν, cm^−1^): 3225(OH); 1716(C = O); 3057, 1610, 1580, 1446, 988, 842 (Ar).1HNMR (600 MHz, DMSO-*d*_6_) δ(ppm): 9.59 (s, 1H), 7.42 (d, *J* = 8.6 Hz, 2H), 7.16 (d, *J* = 16.4 Hz, 1H), 6.94 (d, *J* = 16.4 Hz, 1H), 6.77 (d, *J* = 8.6 Hz, 2H), 6.72 (d, *J* = 2.2 Hz, 2H) and 6.37 (t, *J* = 2.2 Hz, 1H). 13CNMR (150 MHz, DMSO-*d*_6_) δ (ppm): 161.11, 157.85, 140.08, 129.43, 128.43, 128.40, 125.62, 116.03, 104.52, 99.75. MS: *m/z*(%): 238.9[M + 1]^+^, 182.9

***8-Hydroxy-3-arylcoumarin (5).*** White solid, yield 54%, m.p. 199.8–200.1 °C. IR (KBr, ν, cm^−1):3201^(OH); 1681(C = O); 1607, 1572, 1470, 1136, 958(Ar).1HNMR (600 MHz, DMSO-*d*_6_) δ(ppm): 10.24 (s, 1H), 8.20 (s, 1H), 7.73 (d, *J* = 7.7 Hz, 2H), 7.46 (t, *J* = 7.4 Hz, 2H), 7.42 (d, *J* = 7.2 Hz, 1H), 7.18 (dt, *J* = 15.4, 7.6 Hz, 2H), 7.10 (d, *J* = 7.7 Hz, 1H). 13CNMR (150 MHz, DMSO-*d*_6_) δ (ppm): 160.11, 144.80, 142.14, 141.52, 135.22, 128.99, 128.68, 127.18, 125.03, 120.90, 119.10, 118.48. MS: *m/z*(%): 238.9[M + 1]^+^, 210.8, 182.8, 164.7.

***3-(4-Hydroxyphenyl)-coumarin (6).*** White solid, yield 35%, m.p.202.3–203.3 °C. IR (KBr, ν, cm^−1^): 3211(OH); 1688(C = O); 3065, 1610, 1517, 1453, 1118, 957, 814 (Ar).1HNMR (600 MHz, DMSO-*d*_6_) δ(ppm): 9.76 (s, 1H), 8.14 (s, 1H), 7.75 (dd, *J* = 7.7, 1.5 Hz, 1H), 7.60 (m, 2H), 7.58 (m, 1H), 7.41 (d, *J* = 8.3 Hz, 1H), 7.36 (td, *J* = 7.6, 1.0 Hz, 1H), 6.84 (m, 2H). 13CNMR (150 MHz, DMSO-*d*_6_) δ (ppm): 160.37, 158.47, 153.09, 138.97, 131.59, 130.31, 128.79, 127.19, 125.69, 124.98, 120.18, 116.21, 115.53. MS: *m/z*(%): 239.0 [M + 1]^+^, 182.8.

***3-(4-Fluorophenyl)-coumarin (7).*** White solid, yield 50%, m.p.196.7–197.7 °C. IR (KBr, ν, cm^−1^): 1696(C = O); 3058, 1604, 1513, 1455, 1128, 951, 824, 815 (Ar); 1093(C-F).1HNMR (600 MHz, DMSO-*d*_6_) δ(ppm): 8.27 (s, 1H), 7.79 (m, 3H), 7.64 (t, *J* = 7.8 Hz, 1H), 7.45 (d, *J* = 8.3 Hz, 1H), 7.39 (t, *J* = 7.5 Hz, 1H), 7.32 (t, *J* = 8.8 Hz, 2H). 13CNMR (150 MHz, DMSO-*d*_6_) δ (ppm): 163.51, 161.88, 160.20, 153.43, 141.05, 132.24, 131.52, 131.50, 131.24, 131.18, 129.13, 126.33, 125.12, 119.92, 116.36, 115.69, 115.55. MS: *m/z*(%): 241.1[M + 1]^+^, 184.9, 164.8.

***8-Hydroxy-3-(4-methylphenyl)-coumarin (8).*** White solid, yield 56%, m.p.211.7–212.5 °C, IR (KBr, ν, cm^−1^): 3220(OH); 1686(C = O); 1607, 1576, 1514, 1470, 1132, 950, 834 (Ar).1HNMR (600 MHz, DMSO-*d*_6_) δ(ppm): 10.22 (s, 1H), 8.17 (s, 1H), 7.64 (d, *J* = 8.0 Hz, 2H), 7.27 (d, *J* = 8.0 Hz, 2H), 7.18 (m, 2H), 7.10 (m, 1H), 2.36 (s, 3H). 13CNMR (150 MHz, DMSO-*d*_6_) δ (ppm): 160.15, 144.77, 142.03, 140.82, 138.51, 132.30, 129.25, 128.83, 127.04, 124.98, 120.96, 119.00, 118.32, 21.31. MS: *m/z*(%): 253.0[M + 1]^+^, 224.8, 196.8, 178.8.

***7-Hydroxy-3-(4-methylphenyl)-coumarin (9).*** White solid, yield 39%, m.p.242.9–244.1 °C. IR (KBr, ν, cm^−1^): 3236(OH); 1692(C = O); 1591, 1512, 1451, 1123, 943, 819 (Ar).1HNMR (600 MHz, DMSO-*d*_6_) δ (ppm): 10.59 (s, 1H), 8.12 (s, 1H), 7.60 (m, 3H), 7.25 (d, *J* = 7.9 Hz, 2H), 6.82 (dd, *J* = 8.5, 2.3 Hz, 1H), 6.76 (d, *J* = 2.3 Hz, 1H), 2.34 (s, 3H).13CNMR (150 MHz, DMSO-*d*_6_) δ (ppm): 161.56, 160.59, 155.27, 140.94, 137.91, 132.66, 130.35, 129.22, 128.57, 122.57, 113.82, 112.52, 102.16, 21.28. MS: *m/z*(%): 253.0[M + 1]^+^, 224.8, 196.8, 181.8.

***8-Hydroxy-3-(2-methylphenyl)-coumarin (10).*** White solid, yield 37%, m.p.212.7–213.0 °C. IR (KBr, ν, cm^−1^): 3205(OH); 1677(C = O); 3058, 1612, 1471, 1142, 1060, 959 (Ar).1HNMR (600 MHz, DMSO-*d*_6_) δ (ppm): 10.25 (s, 1H), 7.98 (s, 1H), 7.30 (m, 4H), 7.18 (t, *J* = 6.8 Hz, 2H), 7.13 (dd, *J* = 6.9, 2.4 Hz, 1H), 2.22 (s, 3H). 13CNMR (150 MHz, DMSO-*d*_6_) δ (ppm): 159.84, 144.93, 143.20, 142.44, 136.87, 135.56, 130.42, 130.39, 128.92, 128.71, 126.18, 125.03, 120.58, 118.96, 118.50, 19.97. MS: *m/z*(%): 253.0[M + 1]^+^, 224.9, 196.8, 181.7.

***7-Hydroxy-3-(2-methylphenyl)-coumarin (11).*** White solid, yield 41%, m.p.214.1–214.6 °C. IR (KBr, ν, cm^−1^): 3204 (OH); 1677(C = O); 1612, 1471, 1060, 959(Ar). 1HNMR (600 MHz, DMSO-*d*_6_) δ (ppm): 10.60 (s, 1H), 7.90 (s, 1H), 7.57 (d, *J* = 8.5 Hz, 1H), 7.27 (m, 4H), 6.83 (dd, *J* = 8.5, 2.1 Hz, 1H), 6.79 (d, *J* = 2.0 Hz, 1H), 2.20 (s, 3H). 13CNMR (150 MHz, DMSO-*d*_6_) δ (ppm): 161.65, 160.26, 155.69, 143.20, 136.96, 135.87, 130.53, 130.35, 130.29, 128.66, 126.15, 124.17, 113.78, 112.10, 102.35, 20.02. MS: *m/z*(%): 253.1[M + 1]^+^, 224.7, 196.7, 181.6, 146.5.

***7-Hydroxy-3-(4-hydroxyphenyl)-coumarin (12).*** White solid, yield 95%, m.p.327.1–328.0 °C. IR (KBr, ν, cm^−1^): 3386(OH); 1675(C = O); 1607, 1515, 1446, 1121, 951, 835(Ar). 1HNMR (600 MHz, DMSO-*d*_6_) δ (ppm): 10.52 (s, 1H), 9.64 (s, 1H), 8.03 (s, 1H), 7.55 (dd, *J* = 13.6, 8.6 Hz, 3H), 6.82 (m, 3H), 6.74 (d, *J* = 2.1 Hz, 1H). 13CNMR (150 MHz, DMSO-*d*_6_) δ (ppm): 161.20, 160.74, 157.95, 155.00, 139.64, 130.06, 129.99, 126.16, 122.70, 115.45, 113.72, 112.65, 102.12. MS: *m/z*(%): 255.0[M + 1]^+^, 226.9, 198.9, 184.8.

***5,7-Dihydroxy-3-arylcoumarin (13).*** White solid, yield 57%, m.p.259.4–260.9 °C. IR (KBr, ν, cm^−1^): 3259(OH); 1677(C = O); 1609, 1519, 1447, 1124, 1013, 956, 840(Ar). 1HNMR (600 MHz, DMSO-*d*_6_) δ (ppm): 10.75 (s, 1H), 10.43 (s, 1H), 8.07 (s, 1H), 7.66 (m, 2H), 7.43 (dd, *J* = 10.5, 4.7 Hz, 2H), 7.36 (dd, *J* = 8.2, 6.5 Hz, 1H), 6.30 (d, *J* = 2.1 Hz, 1H), 6.24 (d, *J* = 2.0 Hz, 1H). 13CNMR (150 MHz, DMSO-*d*_6_) δ (ppm):162.59, 160.75, 156.68, 156.28, 136.36, 135.92, 128.67, 128.59, 128.22, 120.13, 102.81, 98.83, 94.15. MS: *m/z*(%): 255.0[M + 1]^+^, 226.9, 198.8, 148.8.

***8-Hydroxy-3-(4-hydroxyphenyl)-coumarin (14).*** White solid, yield 51%,m.p.237.8–238.7 °C. IR (KBr, ν, cm^−1^): 3387, 3280(OH); 1703(C = O); 1610, 1513, 1473, 1122, 1060, 952, 837 (Ar).1HNMR (600 MHz, DMSO-*d*_6_) δ (ppm): 10.18 (s, 1H), 9.73 (s, 1H), 8.08 (s, 1H), 7.59 (m, 2H), 7.16 (m, 2H), 7.07 (dd, *J* = 7.3, 2.1 Hz, 1H), 6.85 (m, 2H).13CNMR (150 MHz, DMSO-*d*_6_) δ (ppm): 160.30, 158.41, 144.71, 141.79, 139.41, 130.30, 127.02, 125.77, 124.92, 121.12, 118.79, 117.94, 115.50. MS: *m/z*(%): 254.9[M + 1]^+^, 226.8, 198.8, 164.8, 132.8.

***7,8-Dihydroxy-3-arylcoumarin (15).*** White solid, yield 39%, m.p.254.0–255.0 °C. IR (KBr, ν, cm^−1^): 3508(OH); 1724(C = O); 3052, 1621, 1591, 1514, 1445, 1104, 1022, 955, 814(Ar).

^1^HNMR (600 MHz, DMSO-*d*_6_) δ (ppm): 10.15 (s, 1H), 9.42 (s, 1H), 8.12 (s, 1H), 7.71 (dd, *J* = 5.2, 3.3 Hz, 2H), 7.45 (dd, *J* = 10.3, 4.7 Hz, 2H), 7.38 (m, 1H), 7.11 (d, *J* = 8.5 Hz, 1H), 6.84 (d, *J* = 8.4 Hz, 1H). 13CNMR (150 MHz, DMSO-*d*_6_) δ (ppm): 160.52, 150.08, 143.67, 142.18, 135.68, 132.30, 128.78, 128.63, 128.43, 122.41, 119.68, 113.27, 113.26. MS: *m/z* (%): 254.8[M + 1]^+^, 226.7, 198.7, 184.7, 132.7.

***7-Hydroxy-3-(2-hydroxyphenyl)-coumarin (16).*** White solid, yield 45%, m.p.200.3–202.3 °C. IR (KBr, ν, cm^−1^): 3202(OH); 1676(C = O); 1648, 1582, 1518, 1449, 1136, 959, 852(Ar). 1HNMR (600 MHz, DMSO-*d*_6_) δ (ppm): 10.53 (s, 1H), 9.49 (s, 1H), 7.90 (s, 1H), 7.56 (d, *J* = 8.5 Hz, 1H), 7.24 (d, *J* = 7.3 Hz, 1H), 7.20 (t, *J* = 7.7 Hz, 1H), 6.90 (d, *J* = 8.1 Hz, 1H), 6.84 (t, *J* = 7.4 Hz, 1H), 6.81 (dd, *J* = 8.5, 2.1 Hz, 1H), 6.76 (d, *J* = 1.9 Hz, 1H). 13CNMR (150 MHz, DMSO-*d*_6_) δ (ppm): 161.36, 160.24, 155.54, 155.44, 142.86, 131.35, 130.07, 129.76, 123.07, 121.71, 119.13, 116.11, 113.58, 112.24, 102.26. MS: *m/z* (%): 255.0[M + 1]^+^, 226.9, 198.8, 164.7, 132.7.

***6-Hydroxy-3-(4-hydroxyphenyl)-coumarin (17).*** White solid, yield 73%, m.p.274.8–275.6 °C. IR (KBr, ν, cm^−1^):3265(OH); 1686(C = O); 1606, 1514, 1460, 1132, 975, 812 (Ar).1HNMR (600 MHz, DMSO-*d*_6_) δ (ppm): 9.72 (d, *J* = 4.7 Hz, 2H), 8.05 (s, 1H), 7.58 (m, 2H), 7.25 (d, *J* = 8.9 Hz, 1H), 7.07 (d, *J* = 2.8 Hz, 1H), 7.00 (dd, *J* = 8.9, 2.9 Hz, 1H), 6.84 (m, 2H). 13CNMR (150 MHz, DMSO-*d*_6_) δ (ppm): 160.61, 158.38, 154.24, 146.50, 138.99, 130.33, 127.18, 125.87, 120.71, 119.61, 117.06, 115.47, 112.75. MS: *m/z* (%): 254.6[M + 1]^+^, 226.5, 198.5, 164.5, 132.6.

***7-Hydroxy-3-(4-fluorophenyl)-coumarin (18).*** White solid, yield 45%, m.p.245.7–247.4 °C. IR (KBr, ν, cm^−1^): 3202(OH); 1679(C = O); 1624, 1508, 1453, 1122, 959, 831 (Ar).1HNMR (600 MHz, DMSO-*d*_6_) δ (ppm): 10.63 (s, 1H), 8.16 (s, 1H), 7.75 (m, 2H), 7.60 (d, *J* = 8.5 Hz, 1H), 7.28 (m, 2H), 6.83 (dd, *J* = 8.5, 2.3 Hz, 1H), 6.76 (d, *J* = 2.2 Hz, 1H). 13CNMR (150 MHz, DMSO-*d*_6_) δ (ppm): 163.15, 161.75, 161.52, 160.56, 155.39, 141.58, 131.96, 131.94, 130.89, 130.83, 130.47, 121.63, 115.58, 115.44, 113.91, 112.41, 102.20. MS: *m/z* (%): 256.8[M + 1]^+^, 236.7, 200.8, 164.7.

***8-Hydroxy-3-(4-fluorophenyl)-coumarin (19).*** White solid, yield 52%, m.p.201.3–203.1 °C. IR (KBr, ν, cm^−1^): 3233(OH); 1686(C = O); 1605, 1509, 1472, 1134, 957, 840 (Ar); 1083(C-F).1HNMR (600 MHz, DMSO-*d*_6_) δ (ppm): 10.25 (s, 1H), 8.21 (s, 1H), 7.80 (m, 2H), 7.31 (m, 2H), 7.19 (m, 2H), 7.11 (dd, *J* = 7.2, 2.2 Hz, 1H). 13CNMR (150 MHz, DMSO-*d*_6_) δ (ppm): 163.47, 161.84, 160.13, 144.81, 142.11, 141.48, 131.60, 131.58, 131.23, 131.17, 126.15, 125.06, 120.85, 119.09, 118.51, 115.65, 115.51. MS: *m/z* (%): 257.0[M + 1]^+^, 228.9, 200.9, 182.9, 153.9.

***7-Hydroxy-3-(2,5-dimethylphenyl)-coumarin (20).*** White solid, yield 52%, m.p.189.1–190.6 °C. IR (KBr, ν, cm^−1^): 3218(OH); 11681(C = O); 1593, 1455, 1116, 1013, 951, 815 (Ar).1HNMR (600 MHz, DMSO-*d*_6_) δ (ppm): 10.25 (s, 1H), 8.21 (s, 1H), 7.80 (m, 2H), 7.31 (m, 2H), 7.18 (m, 2H), 7.11 (dd, *J* = 7.2, 2.2 Hz, 1H). 13CNMR (150 MHz, DMSO-*d*_6_) δ (ppm): 161.60, 160.23, 155.66, 143.07, 135.70, 135.04, 133.78, 131.04, 130.27, 130.24, 129.23, 124.32, 113.76, 112.11, 102.34, 20.91, 19.55. MS: *m/z* (%): 267.2[M + 1]^+^, 238.9, 210.9, 192.8, 146.6.

***8-Hydroxy-3-(2,5-dimethylphenyl)-coumarin (21).*** White solid, yield 45%, m.p.183.2–187.4 °C. IR (KBr, ν, cm^−1^): 3243(OH); 1676(C = O); 1611, 1500, 1470, 1060, 947, 816 (Ar).1HNMR (600 MHz, DMSO-*d*_6_) δ (ppm): 10.24 (s, 1H), 7.96 (s, 1H), 7.18 (m, 3H), 7.13 (m, 2H), 7.10 (s, 1H), 2.30 (s, 3H), 2.16 (s, 3H). 13CNMR (150 MHz, DMSO-*d*_6_) δ (ppm): 159.81, 144.92, 143.08, 142.42, 135.40, 135.10, 133.70, 130.91, 130.32, 129.49, 128.86, 125.02, 120.59, 118.92, 118.46, 20.91, 19.50. MS: *m/z* (%): 267.1[M + 1]^+^, 223.9, 210.8, 180.8, 146.6.

***8-Hydroxy-3-(4-methoxyphenyl)-coumarin (22).*** White solid, yield 41%, m.p.223.5–225.8 °C. IR (KBr, ν, cm^−1^): 3394(OH); 1705(C = O); 1607, 1513, 1470, 1125, 1030, 926, 831 (Ar); 1289, 1195(C-O), 2834(-OCH_3_).1HNMR (600 MHz, DMSO-*d*_6_) δ (ppm): 10.20 (s, 1H), 8.14 (s, 1H), 7.71 (m, 2H), 7.16 (m, 2H), 7.05 (ddd, *J* = 7.2, 1.9 Hz, 4H), 3.81 (s, 3H). 13CNMR (150 MHz, DMSO-*d*_6_) δ (ppm): 160.26, 160.02, 144.75, 141.89, 140.04, 130.29, 127.40, 126.71, 124.96, 121.05, 118.89, 118.12, 114.18, 114.13, 55.70. MS: *m/z* (%): 269.0[M + 1]^+^.

***7,8-Dihydroxy-3-(4-methylphenyl)-coumarin (23).*** White solid, yield 43%, m.p.226.8–227.5 °C. IR (KBr, ν, cm^−1^): 3155(OH); 1682(C = O); 1619, 1517, 1315, 1125, 1029, 910, 810 (Ar).1HNMR (600 MHz, DMSO-*d*_6_) δ (ppm): 10.13 (s, 1H), 9.40 (s, 1H), 8.08 (s, 1H), 7.61 (m, 2H), 7.25 (d, *J* = 7.9 Hz, 2H), 7.10 (d, *J* = 8.5 Hz, 1H), 6.84 (d, *J* = 8.4 Hz, 1H), 2.34 (s, 3H). 13CNMR (150 MHz, DMSO-*d*_6_) δ (ppm): 160.56, 149.92, 143.56, 141.51, 137.84, 132.75, 132.27, 129.20, 128.61, 122.32, 119.54, 113.30, 113.23, 21.28. MS: *m/z* (%): 269.1[M + 1]^+^, 222.8, 212.9, 194.9, 166.9.

***7-Hydroxy-3-(4-methoxyphenyl)-coumarin (24).*** White solid, yield 45%, m.p.227.8–228.8 °C. IR (KBr, ν, cm^−1^): 3226(OH); 1697(C = O); 1611, 1513, 1464, 1300, 1030, 947, 827 (Ar); 1247, 1181(C-O), 2834(-OCH_3_).1HNMR (600 MHz, DMSO-*d*_6_) δ (ppm): 10.56 (s, 1H), 8.08 (s, 1H), 7.66 (d, *J* = 8.8 Hz, 2H), 7.58 (d, *J* = 8.5 Hz, 1H), 7.00 (d, *J* = 8.8 Hz, 2H), 6.82 (dd, *J* = 8.5, 2.2 Hz, 1H), 6.75 (d, *J* = 2.1 Hz, 1H), 3.80 (s, 3H). 13CNMR (150 MHz, DMSO-*d*_6_) δ (ppm): 161.38, 160.70, 159.62, 155.12, 140.23, 130.20, 129.98, 127.78, 122.32, 114.09, 113.79, 112.59, 102.15, 55.66. MS: *m/z* (%): 269.0[M + 1]^+^, 240.9, 212.9, 122.9, 106.9.

***5,7-Dihydroxy-3-(4-methylphenyl)-coumarin (25).*** White solid, yield 47%, m.p.220.1–222.0 °C. IR (KBr, ν, cm^−1^): 3382(OH); 1675(C = O); 1589, 1508, 1470, 1013, 831 (Ar).

^1^HNMR (600 MHz, DMSO-*d*_6_) δ (ppm): 10.72 (s, 1H), 10.40 (s, 1H), 8.03 (s, 1H), 7.56 (d, *J* = 8.1 Hz, 2H), 7.23 (d, *J* = 8.0 Hz, 2H), 6.30 (d, *J* = 2.0 Hz, 1H), 6.23 (d, *J* = 1.9 Hz, 1H), 2.34 (s, 3H). 13CNMR (150 MHz, DMSO-*d*_6_) δ (ppm): 162.41, 160.78, 156.55, 156.16, 137.59, 135.71, 133.00, 129.25, 128.42, 120.07, 102.82, 98.80, 94.12, 21.25. MS: *m/z*(%): 269.0[M + 1]^+^, 240.9, 212.9, 148.6.

***7-Methoxy-3-(4-hydroxyphenyl)-coumarin (26).*** White solid, yield 42%, m.p.209.0–209.8 °C. IR (KBr, ν, cm^−1^): 3336(OH); 1685(C = O); 1606, 1506, 1436, 1127, 1023, 947, 821 (Ar); 1269, 1172(C-O).1HNMR (600 MHz, DMSO-*d*_6_) δ (ppm): 9.68 (s, 1H), 8.08 (s, 1H), 7.66 (m, 1H), 7.56 (m, 2H), 6.97 (m, 2H), 6.83 (m, 2H), 3.86 (d, *J* = 1.6 Hz, 3H). 13CNMR (150 MHz, DMSO-*d*_6_) δ (ppm): 162.40, 160.62, 158.10, 154.90, 139.31, 130.05, 129.80, 125.97, 123.65, 115.48, 113.71, 112.98, 100.63, 56.38. MS: *m/z*(%): 268.9[M + 1]^+^, 240.8, 212.8, 162.7.

***6-Methoxy-3-(4-hydroxyphenyl)-coumarin (27).*** White solid, yield 44%, m.p.195.6–197.2 °C. IR (KBr, ν, cm^−1^): 3333(OH); 1683(C = O); 1606, 1515, 1465, 1131, 1036, 972, 837 (Ar); 1265, 1176(C-O).1HNMR (600 MHz, DMSO-*d*_6_) δ (ppm): 9.75 (s, 1H), 8.09 (s, 1H), 7.59 (d, *J* = 8.6 Hz, 2H), 7.35 (d, *J* = 9.0 Hz, 1H), 7.30 (d, *J* = 2.9 Hz, 1H), 7.17 (dd, *J* = 9.0, 3.0 Hz, 1H), 6.85 (d, *J* = 8.6 Hz, 2H), 3.82 (d, *J* = 5.9 Hz, 3H). 13CNMR (150 MHz, DMSO-*d*_6_) δ (ppm): 160.48, 158.49, 156.09, 147.50, 138.83, 130.31, 127.46, 125.75, 120.64, 119.04, 117.28, 115.54, 110.95, 56.13. MS: *m/z*(%): 268.5[M + 1]^+^, 240.4, 212.4, 184.3, 132.4.

***5,7-Dihydroxy-3-(4-hydroxyphenyl)-coumarin (28).*** White solid, yield 29%, m.p.298.1–299.6 °C. IR (KBr, ν, cm^−1^): 3248(OH); 1666(C = O); 1608, 1513, 1444, 1116, 1007, 987, 838 (Ar).1HNMR (600MH_Z_, DMSO-*d*_6_) δ (ppm): 10.85 (s, 1H), 10.51 (s, 1H), 9.71 (s, 1H), 7.96 (s, 1H), 7.50 (d, *J* = 8.6 Hz, 3H), 6.82 (d, *J* = 8.6 Hz, 3H), 6.40 (d, *J* = 2.0 Hz, 1H), 6.24 (d, *J* = 1.9 Hz, 1H). 13CNMR (150 MHz, DMSO-*d*_6_) δ (ppm): 162.16, 161.00, 157.78, 156.41, 155.84, 134.61, 129.75, 126.49, 120.09, 115.50, 102.84, 98.94, 94.00. MS: *m/z*(%): 270.9[M + 1]^+^, 242.8, 214.7, 186.6, 158.6.

***7-Hydroxy-3-(3,4-dihydroxyphenyl)-coumarin (29).*** White solid, yield 22%, m.p.306.0–307.8 °C. IR (KBr, ν, cm^−1^): 3294(OH); 1685(C = O); 1609, 1513, 1454, 1148, 1042, 837 (Ar).1HNMR (600 MHz, DMSO-*d*_6_) δ (ppm): 10.51 (s, 1H), 9.13 (s, 1H), 9.02 (s, 1H), 7.99 (s, 1H), 7.57 (d, *J* = 8.5 Hz, 1H), 7.18 (d, *J* = 2.2 Hz, 1H), 6.99 (dd, *J* = 8.2, 2.2 Hz, 1H), 6.78 (ddd, *J* = 34.2, 17.2, 2.2 Hz, 3H). 13CNMR (150 MHz, DMSO-*d*_6_) δ (ppm): 161.16, 160.65, 154.94, 146.13, 145.23, 139.55, 130.07, 126.59, 122.78, 119.96, 116.26, 115.78, 113.70, 112.65, 102.07. MS: *m/z*(%): 270.8[M + 1]^+^, 252.7, 214.6, 180.6, 162.6.

***7,8-Dihydroxy-3-(4-hydroxyphenyl)-coumarin (30).*** White solid, yield 67%, m.p.287.5–288.9 °C. IR (KBr, ν, cm^−1^): 3448(OH); 1682(C = O); 3087, 1609, 1519, 1439, 1321, 1013, 821 (Ar).1HNMR (600 MHz, DMSO-*d*_6_) δ (ppm): 10.05 (s, 1H), 9.63 (s, 1H), 9.37 (s, 1H), 7.99 (s, 1H), 7.55 (m, 1H), 7.07 (d, *J* = 8.5 Hz, 1H), 6.82 (dt, *J* = 4.3, 2.6 Hz, 1H). 13CNMR (150 MHz, DMSO-*d*_6_) δ (ppm): 160.71, 157.91, 149.55, 143.35, 140.22, 132.23, 130.02, 126.25, 122.45, 119.24, 115.43, 113.44, 113.17. MS: *m/z*(%): 270.9[M + 1]^+^, 242.8, 214.8, 196.8, 168.9.

***5,7-Dihydroxy-3-(4-fluorophenyl)-coumarin (31).*** White solid, yield 46%, m.p.267.0–267.6 °C. IR (KBr, ν, cm^−1^): 3232(OH); 1676(C = O); 1612, 1307, 1008, 951, 826 (Ar); 1071(C-F).1HNMR (600 MHz, DMSO-*d*_6_) δ (ppm): 10.80 (s, 1H), 10.49 (s, 1H), 8.10 (d, *J* = 1.8 Hz, 1H), 7.63 (m, 4H), 6.27 (d, *J* = 36.4 Hz, 2H). 13CNMR (150 MHz, DMSO-*d*_6_) δ (ppm): 162.87, 160.54, 156.82, 156.34, 136.65, 135.11, 131.58, 130.64, 121.42, 118.77, 102.78, 98.87, 94.20. MS: *m/z*(%): 272.9[M + 1]^+^, 226.8, 216.9, 198.9, 186.8, 170.8.

***7,8-Dihydroxy-3-(4-fluorophenyl)-coumarin (32).*** White solid, yield 57%, m.p.259.8–260.7 °C. IR (KBr, ν, cm^−1^): 3172(OH); 1677(C = O); 1612, 1511, 1470, 1156, 1026, 835 (Ar); 1089(C-F). 1HNMR (600 MHz, DMSO-*d*_6_) δ (ppm): 10.17 (s, 1H), 9.43 (s, 1H), 8.12 (s, 1H), 7.76 (m, 2H), 7.27 (dd, *J* = 14.8, 5.9 Hz, 2H), 7.10 (d, *J* = 8.4 Hz, 1H), 6.85 (d, *J* = 8.4 Hz, 1H). 13CNMR (150 MHz, DMSO-*d*_6_) δ (ppm): 163.12, 161.50, 160.54, 150.11, 143.64, 142.15, 132.31, 132.06, 132.03, 130.93, 130.87, 121.39, 119.67, 115.56, 115.42, 113.31, 113.19. MS: *m/z*(%): 272.9[M + 1]^+^, 252.8, 216.9, 198.9.

***7,8-Dihydroxy-3-(2,5-dimethylphenyl)-coumarin (33).*** White solid, yield 45%, m.p.190.3–190.5 °C. IR (KBr, ν, cm^−1^): 3193(OH); 1679(C = O); 1612, 1511, 1465, 1340, 1040, 935, 843 (Ar). 1HNMR (600 MHz, DMSO-*d*_6_) δ (ppm): 10.14 (s, 1H), 9.41 (s, 1H), 7.84 (s, 1H), 7.15 (d, *J* = 7.7 Hz, 1H), 7.11 (dd, *J* = 7.8, 1.2 Hz, 1H), 7.06 (m, 2H), 6.84 (d, *J* = 8.4 Hz, 1H), 2.29 (s, 3H), 2.14 (s, 3H). 13CNMR (150 MHz, DMSO-*d*_6_) δ (ppm): 160.20, 149.94, 143.93, 143.61, 135.82, 135.04, 133.79, 132.41, 131.05, 130.25, 129.19, 124.11, 119.38, 113.18, 112.88, 20.91, 19.56. MS: *m/z*(%): 282.9[M + 1]^+^.

***7,8-Dihydroxy-3-(4-methoxyphenyl)-coumarin (34).*** White solid, yield 49%, m.p.234.4–235.4 °C. IR (KBr, ν, cm^−1^): 3299(OH); 1697(C = O); 1605, 1517, 1470, 1308, 1130, 1012, 830 (Ar). 1HNMR (600 MHz, DMSO-*d*_6_) δ (ppm): 10.09 (s, 1H), 9.39 (s, 1H), 8.05 (s, 1H), 7.67 (m, 2H), 7.08 (d, *J* = 8.5 Hz, 1H), 7.00 (m, 2H), 6.83 (d, *J* = 8.4 Hz, 1H), 3.80 (s, 3H). 13CNMR (151 MHz, DMSO-*d*_6_) δ (ppm): 160.67, 159.59, 149.73, 143.44, 140.80, 132.26, 130.01, 127.88, 122.07, 119.38, 114.07, 113.38, 113.21, 55.66. MS: *m/z*(%): 285.0[M + 1]^+^, 256.9, 238.9, 228.9.

***5,7-Dihydroxy-3-(4-methoxyphenyl)-coumarin (35).*** White solid, yield 54%, m.p.259.4–260.8 °C. IR (KBr, ν, cm^−1^): 3279(OH); 1681(C = O); 1610, 1509, 1474, 1121, 1009, 955, 810 (Ar); 1299, 1181(C-O).1HNMR (600 MHz, DMSO-*d*_6_) δ (ppm): 10.69 (s, 1H), 10.37 (s, 1H), 8.00 (s, 1H), 7.62 (d, *J* = 8.6 Hz, 2H), 6.99 (d, *J* = 8.7 Hz, 2H), 6.29 (d, *J* = 0.8 Hz, 1H), 6.23 (s, 1H), 3.79 (s, 3H). 13CNMR (151 MHz, DMSO-*d*_6_) δ (ppm): 162.20, 160.90, 159.42, 156.43, 156.02, 135.05, 129.81, 128.16, 119.88, 114.11, 102.86, 98.80, 94.10, 55.63. MS: *m/z*(%): 285.1[M + 1]^+^, 270.0, 256.9, 228.9, 122.6.

***7,8-Dihydroxy-3-(3,4-dihydroxyphenyl)-coumarin (36).*** White solid, yield 56%, m.p.294.1–295.5 °C. IR (KBr, ν, cm^−1^): 3313(OH); 1686(C = O); 1610, 1529, 1467, 1306, 1104, 1051, 959, 871 (Ar).1HNMR (600 MHz, DMSO-*d*_6_) δ (ppm): 10.05 (s, 1H), 9.36 (s, 1H), 9.12 (s, 1H), 9.02 (s, 1H), 7.95 (s, 1H), 7.19 (d, *J* = 2.1 Hz, 1H), 7.07 (d, *J* = 8.4 Hz, 1H), 7.00 (dd, *J* = 8.2, 2.1 Hz, 1H), 6.80 (dd, *J* = 17.7, 8.3 Hz, 2H). 13CNMR (150 MHz, DMSO-*d*_6_) δ (ppm): 160.62, 149.51, 146.09, 145.21, 143.29, 140.13, 132.20, 126.69, 122.54, 119.99, 119.25, 116.31, 115.79, 113.44, 113.14. MS: *m/z*(%): 287.0[M + 1]^+^, 268.8, 240.8, 212.8, 184.7.

***7-Hydroxy-3-(3,4-dimethoxyphenyl)-coumarin (37).*** White solid, yield 73%, m.p.220.8–222.1 °C. IR (KBr, ν, cm^−1^): 3355(OH); 1731(C = O); 3068, 1622, 1605, 1519, 1449, 1313, 1013, 860 (Ar); 1276, 1177(C-O), 2842(-OCH_3_).1HNMR (600 MHz, DMSO-*d*_6_) δ (ppm): 10.56 (s, 1H), 8.13 (s, 1H), 7.58 (d, *J* = 8.5 Hz, 1H), 7.31 (dd, *J* = 7.7, 5.1 Hz, 2H), 7.01 (d, *J* = 8.3 Hz, 1H), 6.79 (dd, *J* = 40.3, 5.3 Hz, 2H), 3.80 (m, 6H). 13CNMR (150 MHz, DMSO-*d*_6_) δ (ppm): 159.48, 153.28, 148.73, 147.73, 145.58, 138.78, 126.66, 125.90, 120.78, 119.61, 118.85, 116.12, 111.80, 111.71, 110.82, 55.09, 55.03. MS: *m/z*(%): 299.0[M + 1]^+^, 270.9, 237.9, 182.9, 136.9.

***8-Hydroxy-3-(3,4-dimethoxyphenyl)-coumarin (38).*** White solid, yield 32%, m.p.162.8–163.8 °C. IR (KBr, ν, cm^−1^): 3233(OH); 1685(C = O); 1605, 1516, 1466, 1133, 1024, 868 (Ar); 1294, 1171(C-O), 2834(-OCH_3_).1HNMR (600 MHz, DMSO-*d*_6_) δ (ppm): 10.21 (s, 1H), 8.18 (s, 1H), 7.36 (dd, *J* = 4.4, 2.4 Hz, 2H), 7.18 (m, 2H), 7.09 (dd, *J* = 7.4, 2.1 Hz, 1H), 7.04 (d, *J* = 9.0 Hz, 1H), 3.82 (d, *J* = 7.2 Hz, 6H). 13CNMR (150 MHz, DMSO-*d*_6_) δ (ppm): 160.18, 149.75, 148.76, 144.75, 141.87, 140.22, 127.57, 126.75, 124.97, 121.77, 121.03, 118.88, 118.13, 112.66, 111.84, 56.10, 56.04. MS: *m/z*(%): 298.9[M + 1]^+^, 283.9, 270.9, 237.9, 210.9.

***6-Hydroxy-3-(3,4-dimethoxyphenyl)-coumarin (39).*** White solid, yield 70%, m.p.216.1–217.9 °C. IR (KBr, ν, cm^−1^): 3284(OH); 1663(C = O); 1519, 1451, 1129, 1023, 873 (Ar); 1264, 1173(C-O).1HNMR (600 MHz, DMSO-*d*_6_) δ (ppm): 9.74 (s, 1H), 8.15 (s, 1H), 7.34 (m, 2H), 7.27 (d, *J* = 8.9 Hz, 1H), 7.09 (d, *J* = 2.7 Hz, 1H), 7.03 (m, 2H), 3.81 (d, *J* = 5.9 Hz, 6H). 13CNMR (150 MHz, DMSO-*d*_6_) δ (ppm): 160.48, 154.28, 149.73, 148.73, 146.58, 139.78, 127.66, 126.90, 121.78, 120.61, 119.85, 117.12, 112.80, 112.71, 111.82, 56.09, 56.03. MS: *m/z*(%): 299.1[M + 1]^+^, 284.0, 271.0, 255.8, 237.8.

***3-(4-brominephenyl)-coumarin (40).*** White solid, yield 53%, m.p.192.8–193.8 °C. IR (KBr, ν, cm^−1^): 1714(C = O); 3048, 1607, 1453, 1300, 1115, 1005, 955, 836 (Ar); 513(C-Br).1HNMR (600 MHz, DMSO-*d*_6_) δ (ppm): 8.32 (s, 1H), 7.79 (dd, *J* = 7.7, 1.4 Hz, 1H), 7.71 (m, 2H), 7.68 (m, 2H), 7.64 (ddd, *J* = 8.7, 7.5, 1.6 Hz, 1H), 7.45 (d, *J* = 8.3 Hz, 1H), 7.40 (td, *J* = 7.6, 0.9 Hz, 1H). 13CNMR (150 MHz, DMSO-*d*_6_) δ (ppm): 159.98, 153.49, 141.38, 134.33, 132.44, 131.67, 131.07, 129.24, 126.16, 125.17, 122.47, 119.86, 116.40. MS: *m/z*(%): 301.8[M + 1]^+^, 221.9.

***7-Diethylamino-3-(4-hydroxyphenyl)-coumarin (41).*** White solid, yield 27%, m.p.190.2–191.8 °C. IR (KBr, ν, cm^−1^): 3280(OH); 1686(C = O); 1613, 1519, 1455, 1310, 1135, 1013, 831 (Ar).1HNMR (600 MHz, DMSO-*d*_6_) δ (ppm): 9.57 (s, 1H), 7.94 (s, 1H), 7.53 (d, *J* = 8.3 Hz, 2H), 7.48 (d, *J* = 8.8 Hz, 1H), 6.80 (d, *J* = 8.3 Hz, 2H), 6.71 (d, *J* = 8.7 Hz, 1H), 6.54 (s, 1H), 3.44 (q, *J* = 6.9 Hz, 4H), 1.14 (t, *J* = 7.0 Hz, 6H). 13CNMR (150 MHz, DMSO-*d*_6_) δ (ppm): 161.15, 157.51, 155.89, 150.49, 139.84, 132.00, 129.75, 129.70, 129.14, 126.69, 119.62, 115.39, 109.50, 109.06, 96.60, 46.17, 44.53, 12.82. MS: *m/z*(%): 310.3[M + 1]^+^, 281.1, 254.1.

***5,7-Dihydroxy-3-(3,4-dimethoxyphenyl)-coumarin (42).*** White solid, yield 96%, m.p.322.0–324.1 °C. IR (KBr, ν, cm^−1^): 3468(OH); 1674(C = O); 3050, 1515, 1457, 1320, 1007, 840 (Ar); 1246, 1140(C-O), 2841(-OCH_3_).1HNMR (600 MHz, DMSO-*d*_6_) δ (ppm): 10.70 (s, 1H), 10.39 (s, 1H), 8.03 (s, 1H), 7.24 (m, 2H), 7.00 (d, *J* = 8.5 Hz, 1H), 6.30 (d, *J* = 2.1 Hz, 1H), 6.23 (d, *J* = 1.9 Hz, 1H), 3.80 (dd, *J* = 8.5, 6H). 13CNMR (150 MHz, DMSO-*d*_6_) δ (ppm): 162.24, 160.85, 156.46, 156.00, 149.13, 148.72, 135.28, 128.43, 121.19, 120.00, 112.42, 111.94, 102.83, 98.82, 94.09, 56.03, 56.00. MS: *m/z*(%): 315.0[M + 1]^+^, 298.9, 270.8, 244.9.

***7,8-Dihydroxy-3-(3,4-dimethoxyphenyl)-coumarin (43).*** White solid, yield 51%, m.p.290.5–291.9 °C. IR (KBr, ν, cm^−1^): 3402(OH); 1685(C = O); 1611, 1519, 1113, 1022, 847 (Ar); 1262(C-O).1HNMR (600 MHz, DMSO-*d*_6_) δ (ppm): 10.10 (s, 1H), 9.38 (s, 1H), 8.09 (s, 1H), 7.31 (m, 2H), 7.09 (d, *J* = 8.5 Hz, 1H), 7.01 (d, *J* = 8.2 Hz, 1H), 6.83 (d, *J* = 8.4 Hz, 1H), 3.80 (d, *J* = 8.6 Hz, 7H). 13CNMR (150 MHz, DMSO-*d*_6_) δ (ppm): 160.59, 149.74, 149.30, 148.74, 143.43, 140.99, 132.25, 128.10, 122.13, 121.43, 119.39, 113.36, 113.22, 112.55, 111.87, 56.07, 56.03. MS: *m/z*(%): 315.0[M + 1]^+^, 299.9, 287.0, 271.9, 259.0.

***7-Hydroxy-3-(4-brominephenyl)-coumarin (44).*** White solid, yield 56%, m.p.270.5–271.6 °C. IR (KBr, ν, cm^−1^): 3231(OH); 1677(C = O); 1589, 1449, 1122, 943, 856 (Ar); 528(C-Br).1HNMR (600 MHz, DMSO-*d*_6_) δ (ppm): 10.66 (s, 1H), 8.21 (s, 1H), 7.68 (d, *J* = 8.6 Hz, 2H), 7.64 (d, *J* = 8.6 Hz, 2H), 7.61 (d, *J* = 8.5 Hz, 1H), 6.84 (dd, *J* = 8.5, 2.0 Hz, 1H), 6.76 (d, *J* = 1.9 Hz, 1H). 13CNMR (150 MHz, DMSO-*d*_6_) δ (ppm): 161.96, 160.34, 155.49, 141.89, 134.78, 131.58, 130.78, 130.62, 121.76, 121.35, 113.99, 112.38, 102.22. MS: *m/z*(%): 318.2[M + 1]^+^, 237.8, 209.8.

***8-Hydroxy-3-(4-brominephenyl)-coumarin (45).*** White solid, yield 56%, m.p.235.5–236.9 °C. IR (KBr, ν, cm^−1^): 3233(OH); 1686(C = O); 1608, 1469, 1131, 1012, 839 (Ar); 524(C-Br).1HNMR (600 MHz, DMSO-*d*_6_) δ (ppm): 10.26 (s, 1H), 8.25 (s, 1H), 7.71 (m, 2H), 7.67 (m, 2H), 7.19 (m, 2H), 7.12 (dd, *J* = 7.5, 2.0 Hz, 1H). 13CNMR (150 MHz, DMSO-*d*_6_) δ (ppm): 159.91, 144.82, 142.17, 141.81, 134.41, 131.63, 131.07, 125.97, 125.11, 122.38, 120.79, 119.19, 118.70. MS: *m/z*(%): 318.4[M + 1]^+^, 237.5, 209.6.

***5,7-Dihydroxy-3-(4-brominephenyl)-coumarin (46).*** White solid, yield 52%, m.p.288.8–289.8 °C. IR (KBr, ν, cm^−1^): 3249(OH); 1678(C = O); 1615, 1509, 1471, 1009, 830 (Ar); 564(C-Br).1HNMR (600 MHz, DMSO-*d*_6_) δ (ppm): 10.75 (s, 1H), 10.44 (s, 1H), 8.05 (s, 1H), 7.71 (m, 2H), 7.25 (t, *J* = 8.9 Hz, 2H), 6.29 (d, *J* = 2.1 Hz, 1H), 6.23 (d, *J* = 2.0 Hz, 1H). 13CNMR (150 MHz, DMSO-*d*_6_) δ (ppm): 162.62, 160.76, 156.68, 156.25, 136.35, 132.29, 131.07, 130.72, 130.67, 119.11, 115.57, 115.43, 102.76, 98.84, 94.15. MS: *m/z*(%): 333.9[M + 1]^+^.

***7,8-Dihydroxy-3-(4-brominephenyl)-coumarin (47).*** White solid, yield 55%, m.p.275.1–276.8 °C. IR (KBr, ν, cm^−1^): 3274(OH); 1692(C = O); 1619, 1510, 1465, 1316, 1129, 1010, 825 (Ar); 524(C-Br).1HNMR (600MH_Z_, DMSO-*d*_6_) δ (ppm): 10.22 (s, 1H), 9.45 (s, 1H), 8.17 (s, 1H), 7.69 (m, 2H), 7.64 (m, 2H), 7.11 (d, *J* = 8.5 Hz, 1H), 6.85 (d, *J* = 8.4 Hz, 1H). 13CNMR (150 MHz, DMSO-*d*_6_) δ (ppm): 160.31, 150.33, 143.71, 142.47, 134.88, 132.32, 131.56, 130.82, 121.69, 121.09, 119.85, 113.37, 113.15. MS: *m/z*(%): 334.5[M + 1]^+^, 253.5, 225.5.

### 4.2. Biological activity

#### 4.2.1. Animals

Normoglycemic Kunming mice, weight 18–20 g, were obtained from Jinan PengYue Experimental Animal Co., Ltd. Jinan, China, (License number: SCXK (Lu) 2014–0007). The animals were housed under standard laboratory conditions and maintained on a standard pellet diet and water *ad libitum*. All experiments involving living animals and their care were performed in strict accordance with the National Care and Use of Laboratory Animals by the National Animal Research Authority (China) and guidelines of Animal Care and Use issued by University of Jinan Institutional Animal Care and Use Committee. The experiments were approved by the Institutional Animal Care and Use Committee of the School of Medicine and Life Sciences, University of Jinan. All efforts were made to minimise animal’s suffering and to reduce the number of animals used.

#### 4.2.2. Influence on blood pressure

Influence on blood pressure was analyzed in adult normotensive mice (25–30 g). After 1 week of acclimation, mean arterial pressure values were measured using the tail-cuff method with a blood pressure monitor (BP-2010, Softron Beijing Biotechnology Co., Ltd.) from 0 to 8 h after administration of the standard and test compounds. The test compounds (40 mg/kg and 100mg/kg) and standard (Losartan, 20 mg/kg) were administered.

#### 4.2.3. *In vitro* antioxidant activity

The ability of the target compounds and vitamin C to scavenge the hydroxyl radical was evaluated with method described by Ismaili et al.^29^ with slight modifications. In test tube, 1.0 ml H_2_O_2_ solution (0.03%), 1.0 ml FeSO_4_ solution (2 mmol.L^−1^), 1.0 ml salicylic acid solution (10 mmol.L^−1^) and 1.0 ml of 3-arylcoumarins and standard water solution with different concentration (0.4–2.0 mg/mL) were mixed. Finally, 1.0 ml H_2_O_2_ solution (0.03%) was added to this mixture to start the reaction. Solutions were kept at 37 °C. After 30 min, the absorbance was measured at 510 nm against the reagent blank, where the distilled water was substituted for the H_2_O_2_ solution. The percentage of OH scavenging by target compounds and vitamin C was calculated the following equation:

$$\text{OH}\;\text{scavenging}\;\text{effect}\;(\%)=[A_{0}-(A_{1}-A_{2})]/A_{0}\times 100\%$$

where *A*_0_ is the absorbance of 1.0 ml distilled water +1.0 ml H_2_O_2_ + 1.0 ml FeSO_4_ + 1.0 ml salicylic acid +1.0 ml H_2_O_2_; *A*_1_ is the absorbance of 1.0 ml 3-arylcoumarins or standard +1.0 ml H_2_O_2_ + 1.0 ml FeSO_4_ + 1.0 ml salicylic acid +1.0 ml H_2_O_2_; *A*_2_ is the absorbance of 1.0 ml 3-arylcoumarins or standard +1.0 ml H_2_O_2_ + 1.0 ml FeSO_4_ + 1.0 ml absolute absolute ethanol +1.0 ml H_2_O_2_.

The effect of target compounds and vitamin C to scavenge the DPPH free radical was evaluated with the method described by Villano et al.^43^ with slight modifications.

Two millilitres of different concentrations of the sample solution was added to 2.0 ml of DPPH solution (0.04 mg/mL), shaken and allowed to react at room temperature for 30 min. The absorbance value was measured at 517 nm with an ultraviolet-visible spectrophotometer. The blank reference cuvette contained absolute ethanol. All measurements were performed in triplicate. IC_50_ values were calculated. The percentage of DPPH radical scavenging rate of the target compound is calculated as follows:

$$\text{DPPH}\;\text{free}\;\text{radical}\;\text{scavenging}\;\text{effect}\;(\%)=\left[A_{0}-\left(A_{1}-A_{2}\right)\right]/A_{0}\times 100\%$$

where *A*_0_ is the absorbance of 2.0 ml DPPH solution +2.0 ml absolute ethanol; *A*_1_ is the absorbance of 2.0 ml DPPH solution +2.0 ml 3-arylcoumarins solution or standard; *A*_2_ is the absorbance of 2.0 ml 3-arylcoumarins solution or standard +2.0 ml absolute ethanol.

#### 4.2.4. *In vitro* α-glucosidaseinhibitory activity

α-Glucosidase (G0660-750UN, Sigma Aldrich) and 4-Nitrophenyl α-d-glucopyranoside (PNPG, Macklin) were dissolved in phosphate buffer (pH 6.8, 100 mM), and the test compounds were dissolved in DMSO solution. The experiment was divided into blank group, control group, sample blank group, and sample group. The reagents were loaded in 96-well plates at the dose of the table (Table 8). The solution was bathed in 37 °C water for 10 min, and then enzyme solution was added. After reaction at 37 °C for 20 min, 70 μL Na_2_CO_3_ solution (0.2 mM) was added to stop the reaction. All experiments were run in triplicate. Acarbose (Sigma Aldrich) was used as a standard inhibitor. Since PNPG can produce glucose and *p*-Nitrophenol (PNP) under the action of α-glucosidase, PNP has the greatest absorption at 405 nm. The absorbance was determined by the microplate reader, and the inhibition rate of α-glucosidase and the IC_50_ value of each sample were calculated according to the formula.

$$\text{Inhibition}\;\text{rate}\;\%=\left\{\left[\left(A_{C}-A_{B}\right)-\left(A_{S}-A_{\text{SB}}\right)\right]/\left(A_{C}-A_{B}\right)\right\}\times 100\%$$

where *A*_C_ is the absorbance of control group; *A*_B_ is the absorbance of blank group; *A*_S_ is the absorbance of sample group; *A*_SB_ is the absorbance of sample blank group.

**Table 8.** The amount and order of each reactant of α-glucosidase inhibition test.

Reagents	Volume (μL)			
	Blank group	Control group	Sample blank group	Sample group
PBS	20	10	20	10
Compounds/inhibitors	0	0	10	10
PNPG	20	20	20	20
Water	10	10	0	0
Mix well and incubate at 37 °C for ten minutes				
α-Glucosidase	0	10	0	10
Mix well and react at 37 °C for 20 minutes				
Na_2_CO_3_	70	70	70	70

#### 4.2.5. *In vitro* inhibitory activity of AGEs formation

To prepare the AGEs reaction solution, 10 mg/mL of bovine serum albumin in 50 mM PBS (pH 7.4) was added to 0.2 M glucose, and 0.02% sodium azide was added to prevent bacterial growth. The reaction mixture (3.0 ml) was then mixed with various concentrations (0.5–1000 μg/mL) of the target compounds (1.0 ml) dissolved in DMSO. After incubating at 37 °C for 14 d, the fluorescence intensity of AGE was determined by a fluorospectrophotometer (PE, USA) with excitation and emission wavelengths at 350 nm and 420 nm, respectively. All experiments were run in triplicate. Aminoguanidine hydrochloride was used as a reference compound. The inhibition rate of AGEs formation and the IC_50_ value of each sample were calculated according to the formula.

$$\text{Inhibition}\;\text{rate}\;\%=\left\{\left[\left(A_{C}-A_{B}\right)-\left(A_{S}-A_{\text{SB}}\right)\right]/\left(A_{C}-A_{B}\right)\right\}\times 100\%$$

where *A*_C_ is the absorbance of control group(1.0 ml glucose +1.0 ml bovine serum albumin +1.0 ml sodium azide +1.0 ml DMSO); *A*_B_ is the absorbance of blank group(1.0 ml PBS +1.0 ml bovine serum albumin +1.0 ml sodium azide +1.0 ml DMSO); *A*_S_ is the absorbance of sample group (1.0 ml glucose +1.0 ml bovine serum albumin +1.0 ml sodium azide +1.0 ml target compound solution or Aminoguanidine hydrochloride solution); *A*_SB_ is the absorbance of sample blank group (1.0 ml PBS +1.0 ml bovine serum albumin +1.0 ml sodium azide +1.0 ml target compound solution or Aminoguanidine hydrochloride solution).

#### 4.2.6. Oral toxicity to mice

Experiments were performed on Kunming mice (male and female half, bw range, 25–30 g). Mice were housed in a climate and light controlled room with a 12 h light/dark cycle. Twelve hours before experiments, food was withheld, but animals had free access to drinking water. The compounds were suspended in vehicle (Tween-80, 0.2% in saline). The concentrations were adjusted to orally administrate 0.2 ml/10 g of bw. Mice were treated in two phases. In the first, intragastric doses of 10, 100 and 1000 mg/kg of bw of compounds were administered. On the second, the doses were adjusted to 1600, 2900, and 5000 mg/kg of bw of compounds. In both phases, mice were observed daily in a period of 14 days for mortality, toxic effects and/or changes in behavioural pattern. At the end of the experiments, the mice were sacrificed in a CO_2_ chamber.

#### 4.2.7. Acute hypoglycemic assay

Type II diabetes mellitus was induced in mice by a single intraperitoneal injection of freshly prepared STZ (Sigma Aldrich) dissolved in 0.1M citrate buffer, pH 4.5, in a volume of 110 mg/kg of bw. After 7 days of STZ administration, blood glucose levels of each mouse were determined. Mice with blood glucose levels higher than 11 mM were considered diabetic and were included in the study.

STZ-induced diabetic mice and normal mice were placed in single cages with wire-net floors and deprived of food for 12 h before experimentation but allowed free access to tap water throughout. The compounds (at the doses of 10, 30, and 100 mg/kg of bw) were suspended in 0.05% Tween-80 in saline solution. Glibenclamide (10 mg/kg of bw) was suspended in the same vehicle. The target compounds were freshly prepared immediately before experimentation and administered by the intragastrical route at the doses of 10 ml/kg of bw. Control mice received only the vehicle (0.05% Tween-80 in saline solution) by the same route. Blood glucose levels were measured at 0, 1.5, 3, 5, 7, and 9 h after drugs administration.

#### 4.2.8. Effects of daily treatment with compounds 11, 17, and 35 in STZ-induced diabetic mice

Type II diabetes mellitus was induced in 24 mice by the same method showed in the section of the induction of experimental diabetes. Thereafter, the target compounds **11**, **17**, **35**, and glibenclamide were administered once (9 a.m.) on a daily basis, for a period of 16 days, to STZ-induced diabetic mice which were divided into five groups (A-E). A six group of normal mice (Group F) was used as a control. Groups E (diabetic control) and F received vehicle (0.05% Tween-80 in saline solution) *per os* for 16 days. Group A received glibenclamide (10 mg/kg of bw daily). Groups B–D received different treatments: B, compound **11** (30 mg/kg of bw daily); C, compound **17** (30 mg/kg of bw daily); D, compound **35** (30 mg/kg of bw daily). All tested materials were suspended in the same vehicle and administered *per os* daily for 16 days. The animals were housed under standard laboratory conditions and maintained with free access to water and food during all the experiment. Blood glucose concentration on animals of diabetic (Groups A–E) and control group (Group F) was estimated at days 1, 4, 7, 10, 13, and 16. The bw of the animals were measured concomitantly to the blood glucose analyses. All experiments were carried out using six animals per group.

#### 4.2.9. Oral glucose tolerance test of compounds 11, 17, and 35 in STZ-induced diabetic mice

The target compounds **11**, **17**, and **35** (30 mg/kg of bw) and control drugs (glibenclamide 10 mg/kg of bw) were dissolved in the vehicle (Tween-80, NaCl 0.9%). Glucose (2 g/kg of bw) was used for the glucose tolerance tests. The control mice group received only the vehicle. All cases were treated orally. Blood samples were collected from caudal vein by means of a small incision in the end of the tail. Blood glucose levels were estimated by a commercial glucometer (Bayer Contour TS). For the anti-hyperglycemic tests, blood samples were obtained 0.5, 1, 1.5, 2, and 3 h after the glucose load. Percentage variation of glycemia for each group was calculated with respect to initial (0 h) level according to:

$$\%\;\text{Variation}\;\text{of}\;\text{glycemia}=\left[\left(G_{i}-G_{t}\right)/G_{i}\right]\times 100\%$$

where *G*_i_ is initial glycemia values and *G*_t_ is the glycemia value after samples administration. For the anti-hyperglycemic tests, data were represented as % variation of glycemia versus time. Studies were performed in normoglycemic and STZ-induced diabetic mice.

#### 4.2.10. Statistical analysis

Data were shown as mean ± SD Differences between individual groups were analyzed by using ANOVA followed by Dunett’s test. A difference with a *p* value of <0.05 was considered to be significant.

## Ethical Statement

All experiments involving living animals and their care were performed in strict accordance with the National Care and Use of Laboratory Animals by the National Animal Research Authority (China) and guidelines of Animal Care and Use issued by University of Jinan Institutional Animal Care and Use Committee. The experiments were approved by the Institutional Animal Care and Use Committee of the School of Medicine and Life Sciences, University of Jinan. All efforts were made to minimise animal’s suffering and to reduce the number of animals used.

## Supplementary Material

IENZ_1518958_Supplementary Material
