# Supplementary material for: Synthesis and biological evaluation of 3-arylcoumarin derivatives as potential anti-diabetic agents
Source: J Enzyme Inhib Med Chem. 2018 Oct 26;34(1):15–30. doi: 10.1080/14756366.2018.1518958 (PMC6211316; doi:10.1080/14756366.2018.1518958)

**Synthesis, and biological evaluation of 3-Arylcoumarin derivatives as potential antidiabetic agents**

Yuheng Hu^a,b,c,d^ , Bing Wang^a,b,c,d^, Jie Yang^a,b,c,d^, Teng Liu^a,b,c,d^, Jie Sun^a,b,c,d^*, Xiaojing Wang^a,b,c,d^*

^a^ *School of Medicine and Life Sciences, University of Jinan-Shandong Academy of Medical Sciences, Jinan 250200, Shandong, China*

^b^ *Institute of MateriaMedica, Shandong Academy of Medical Sciences, Jinan 250062, Shandong, China*

^c^ *Key Laboratory for Biotech-Drugs Ministry of Health, Jinan 250062, Shandong, China*

^d^ *Key Laboratory for Rare & Uncommon Diseases of Shandong Province, Jinan 250062, Shandong, China*

*Corresponding authors: E-Mail:

E-Mail: [sunjie310@126.com](mailto:sunjie310@126.com) (J. Sun)

E-Mail: [xiaojing6@gmail.com](mailto:xiaojing6@gmail.com) (X. Wang)

**Supporting Information**

**Contents**

[**IR(KBr) of compounds** **1**-**44** 2](#_Toc510451881)

[**Mass spectrum of compounds 1-44** 24](#_Toc510451882)

[**^1^H NMR(600 MHz, DMSO-d_6_) of compounds 1-44** 78](#_Toc510451883)

[**^13^C NMR(600 MHz, DMSO-d_6_) of compounds 1-44** 100](#_Toc510451884)

**IR(KBr) of compounds** **1**-**44**

**1**

**2**

**3**

**4**

**5**

**6**

**7**

**8**

**9**

**10**

**11**

**12**

**13**

**14**

**15**

**16**

**17**

**18**

**19**

**20**

**21**

**22**

**23**

**24**

**25**

**26**

**27**

**28**

**29**

**30**

**31**

**32**

**33**

**34**

**35**

**36**

**37**

**38**

**39**

**40**

**41**

**42**

**43**

**44**

**Mass spectrum of compounds 1-44**

**1**


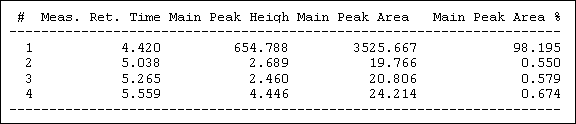


**2**


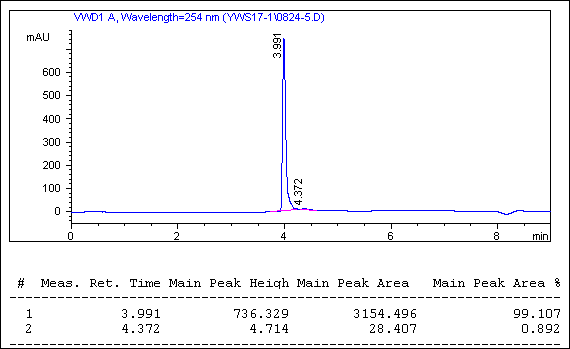


**3**


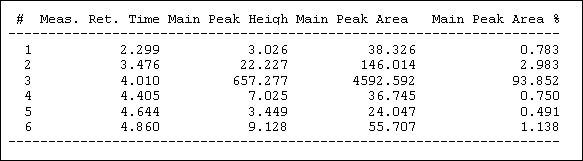


**4**

**5**


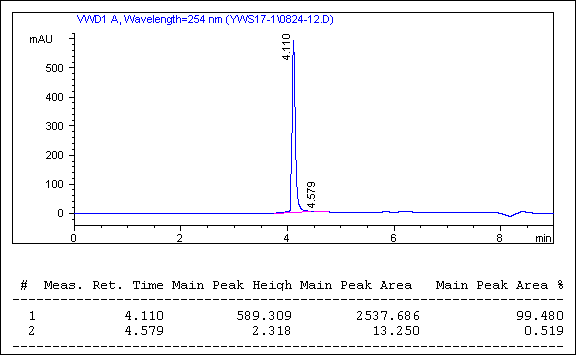


**6**


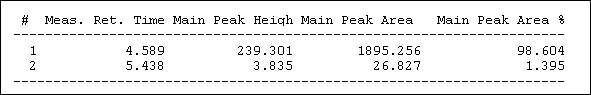


**7**

**8**

**9**


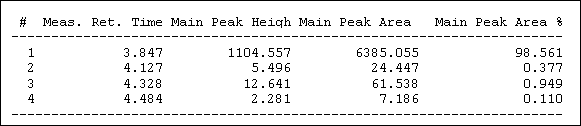


**10**


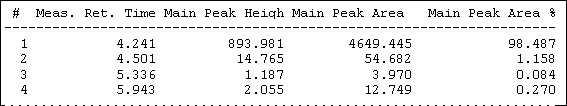


**11**


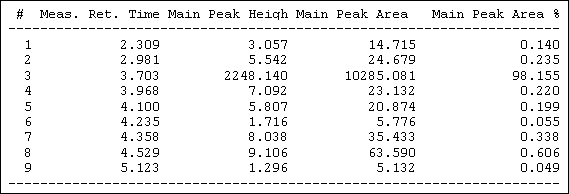


**12**


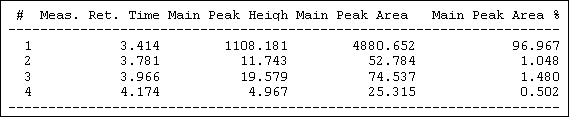


**13**


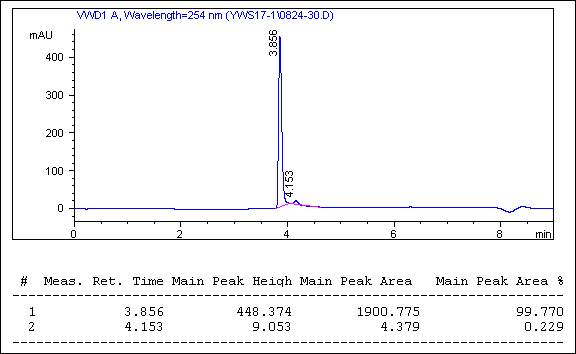


**14**


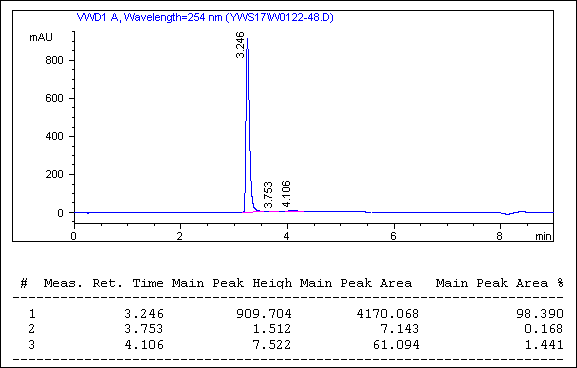


**15**

**16**


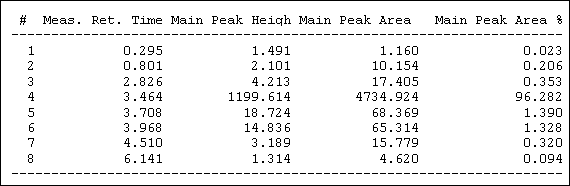
2

**17**

**18**

**19**


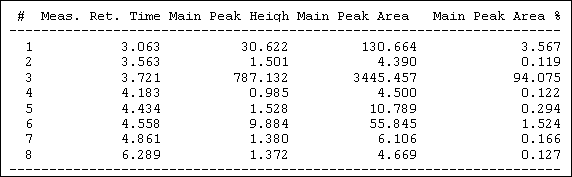


**20**


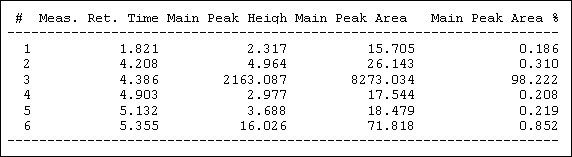


**21**

**22**


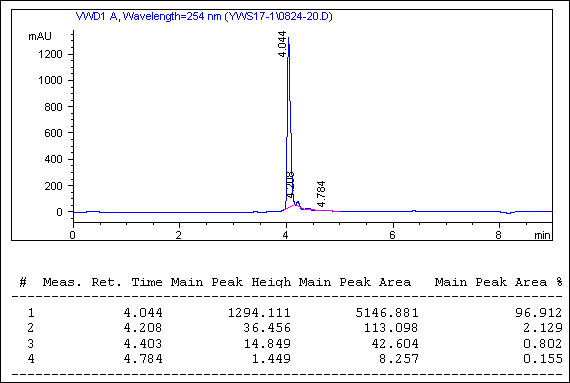


**23**

**24**


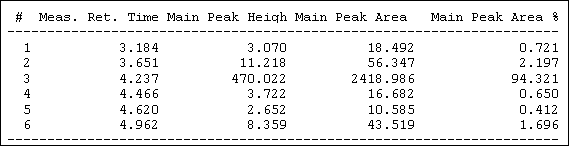


**25**


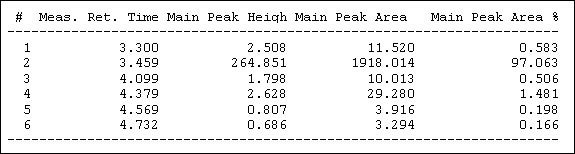


**26**


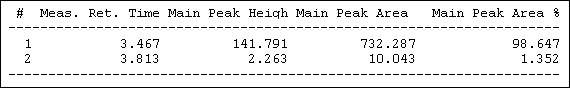


**27**


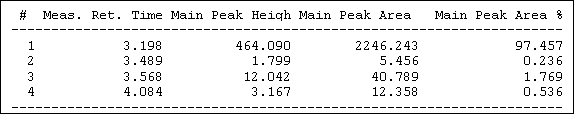


**29**


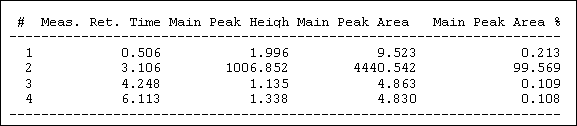


**30**


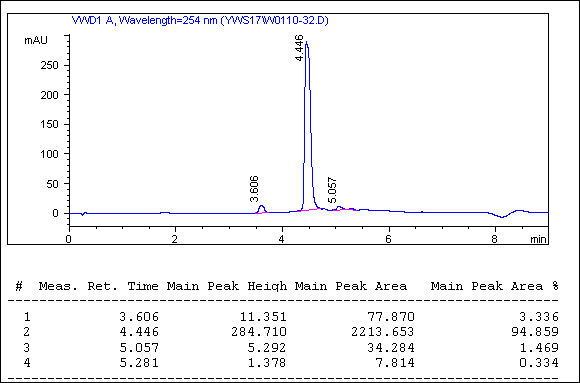


**31**

**32**


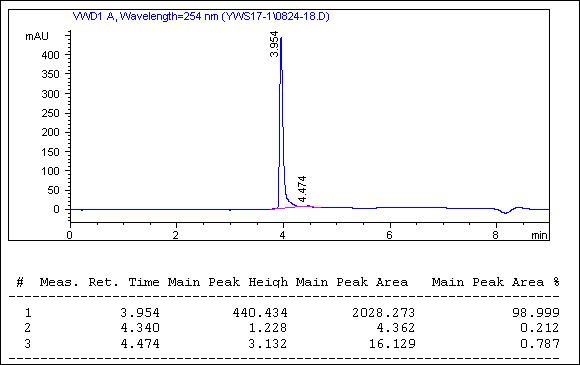


**33**

**34**


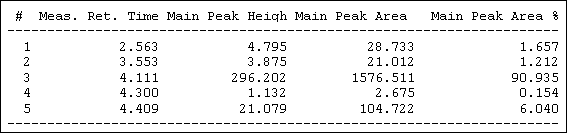


**35**


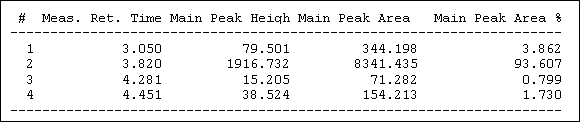


**36**

**37**

**38**


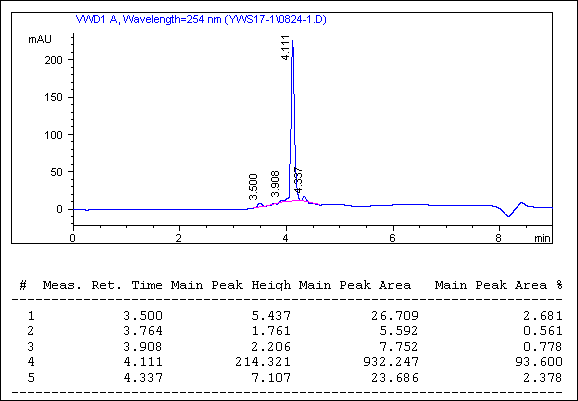


**39**


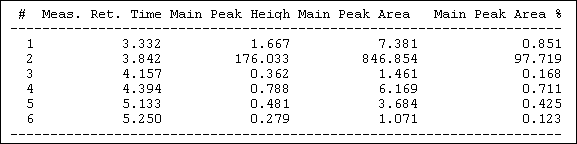


**40**

**41**

**42**

**44**

**^1^H NMR(600 MHz, DMSO-d_6_) of compounds 1-44**


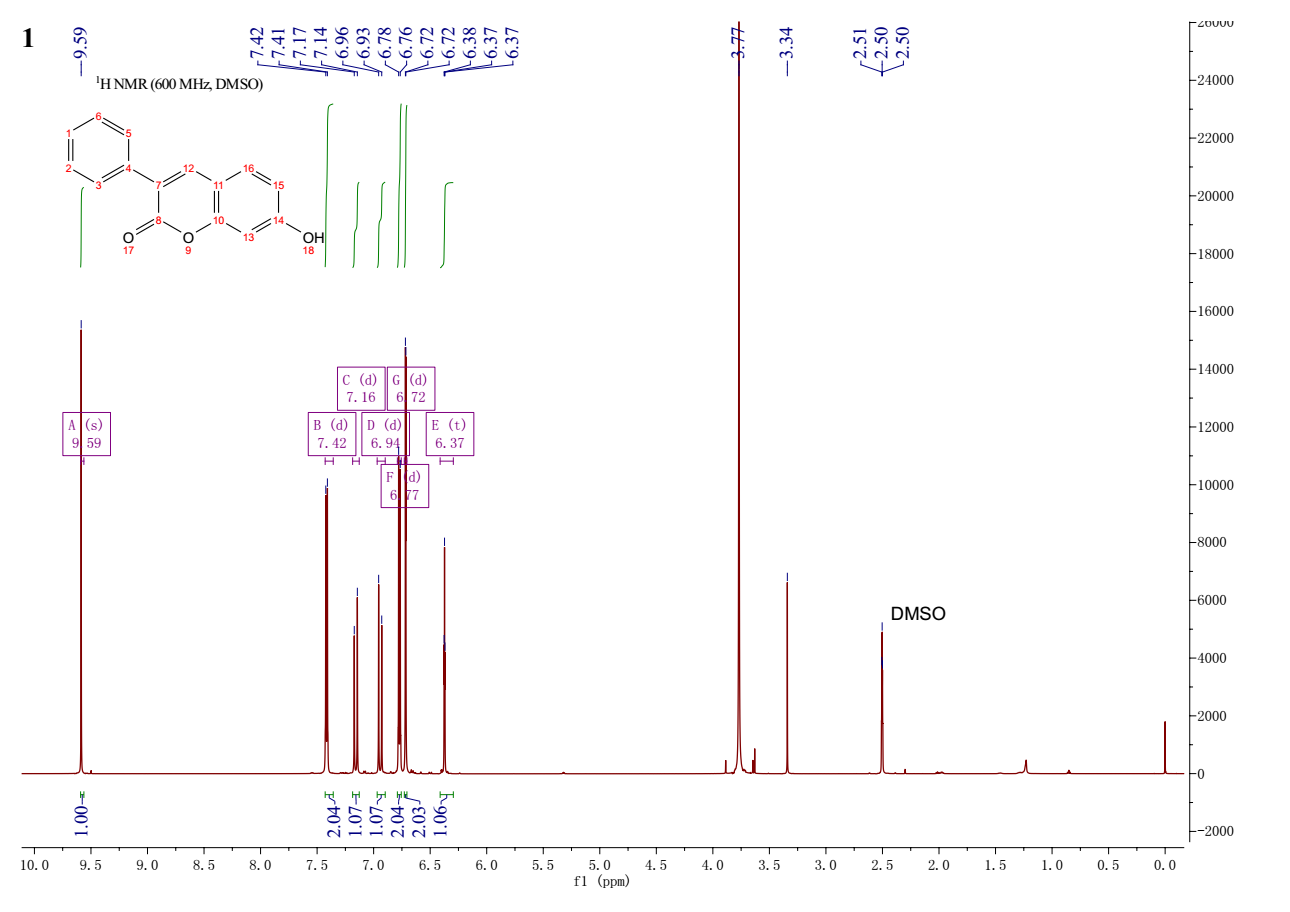


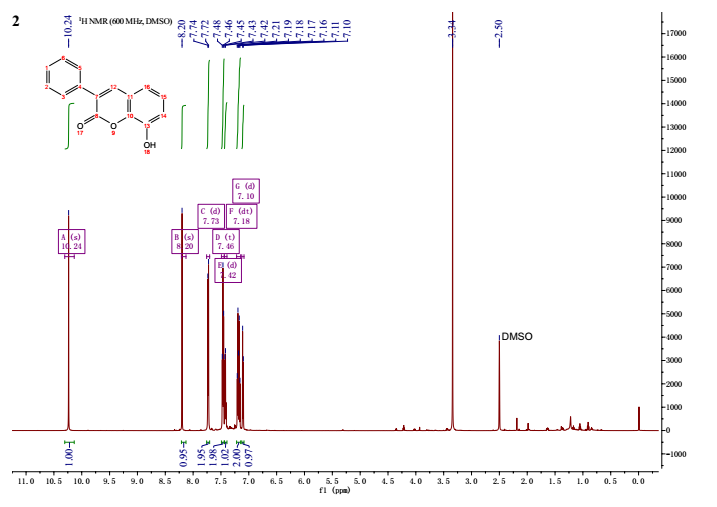


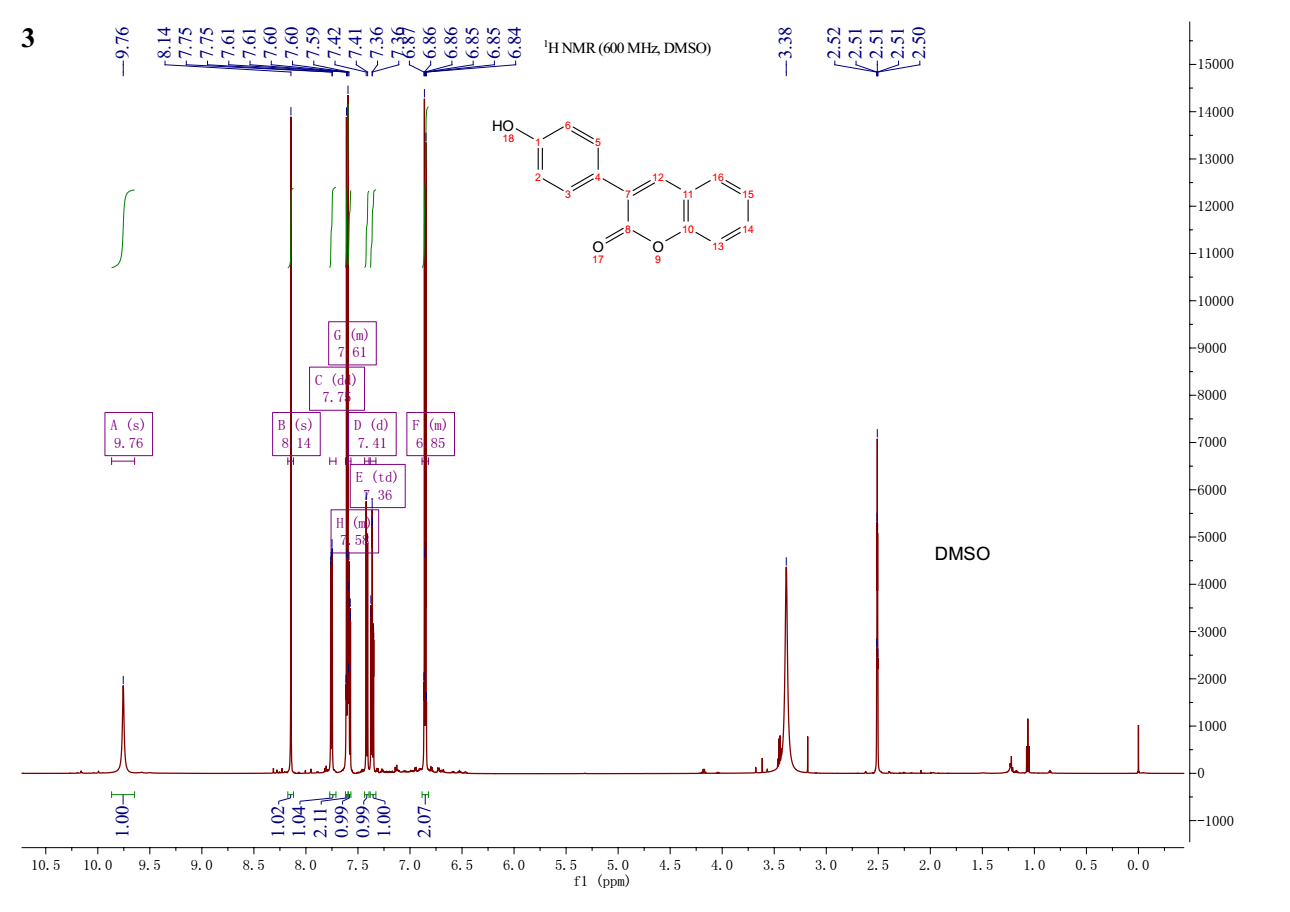


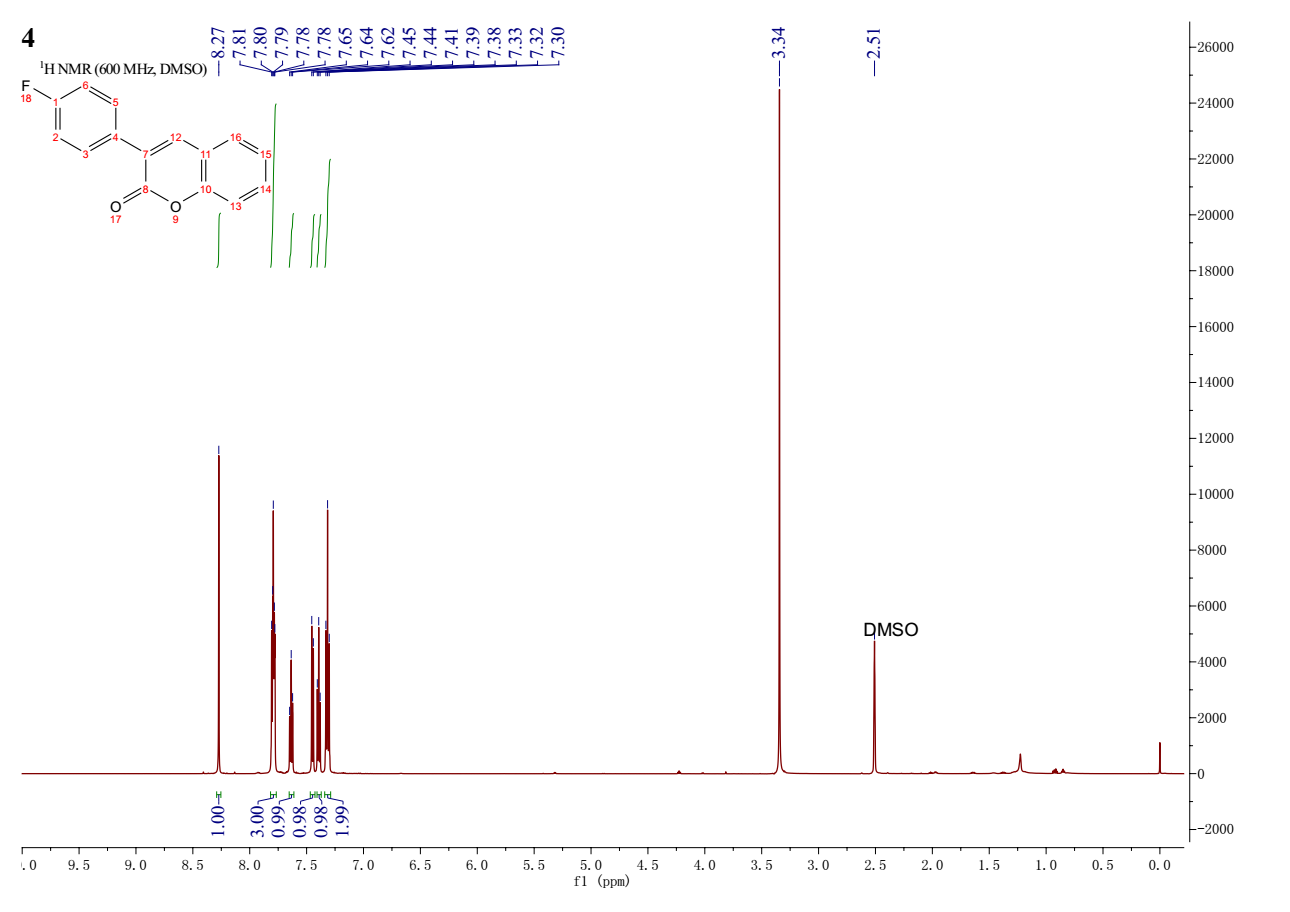


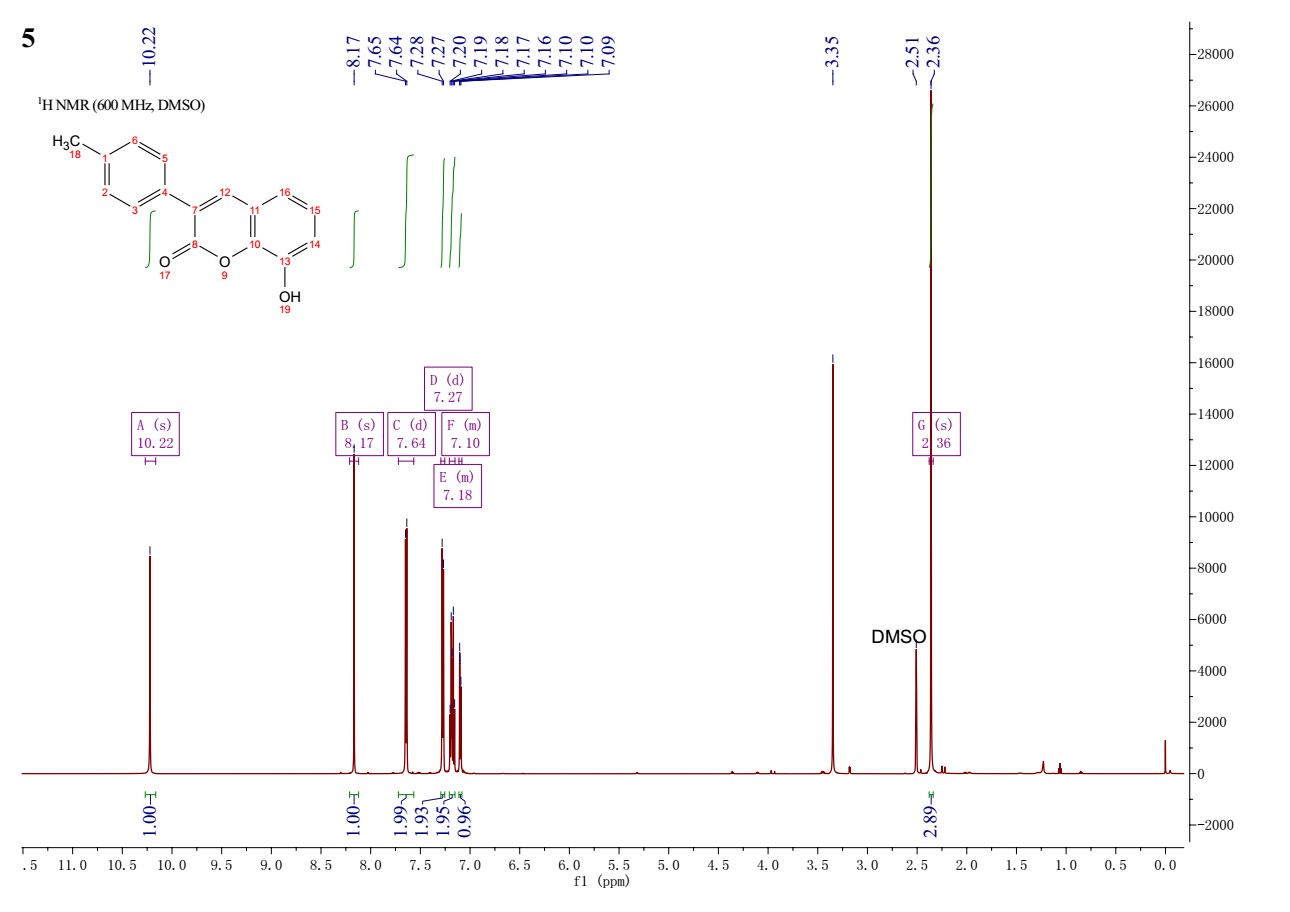


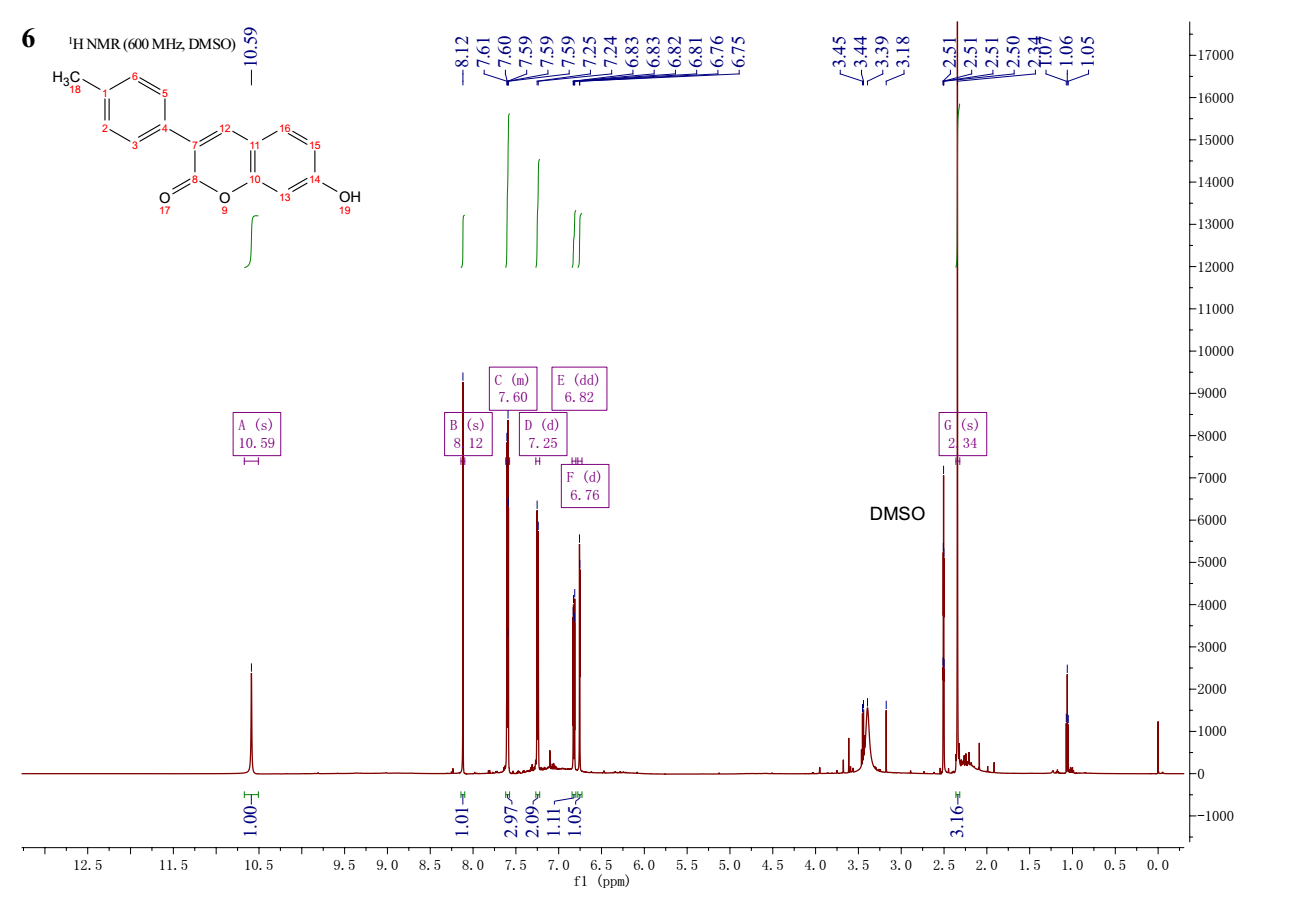


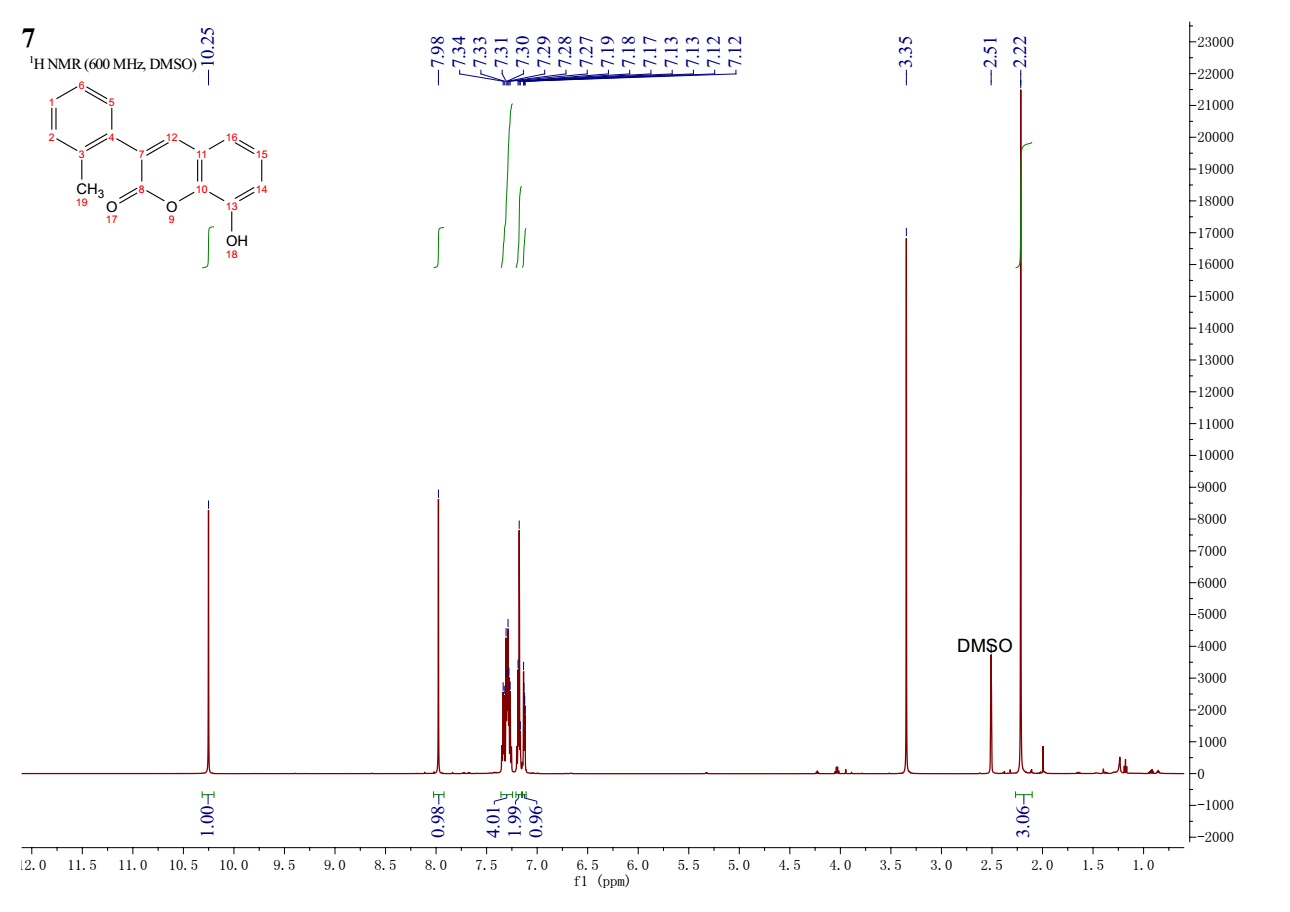


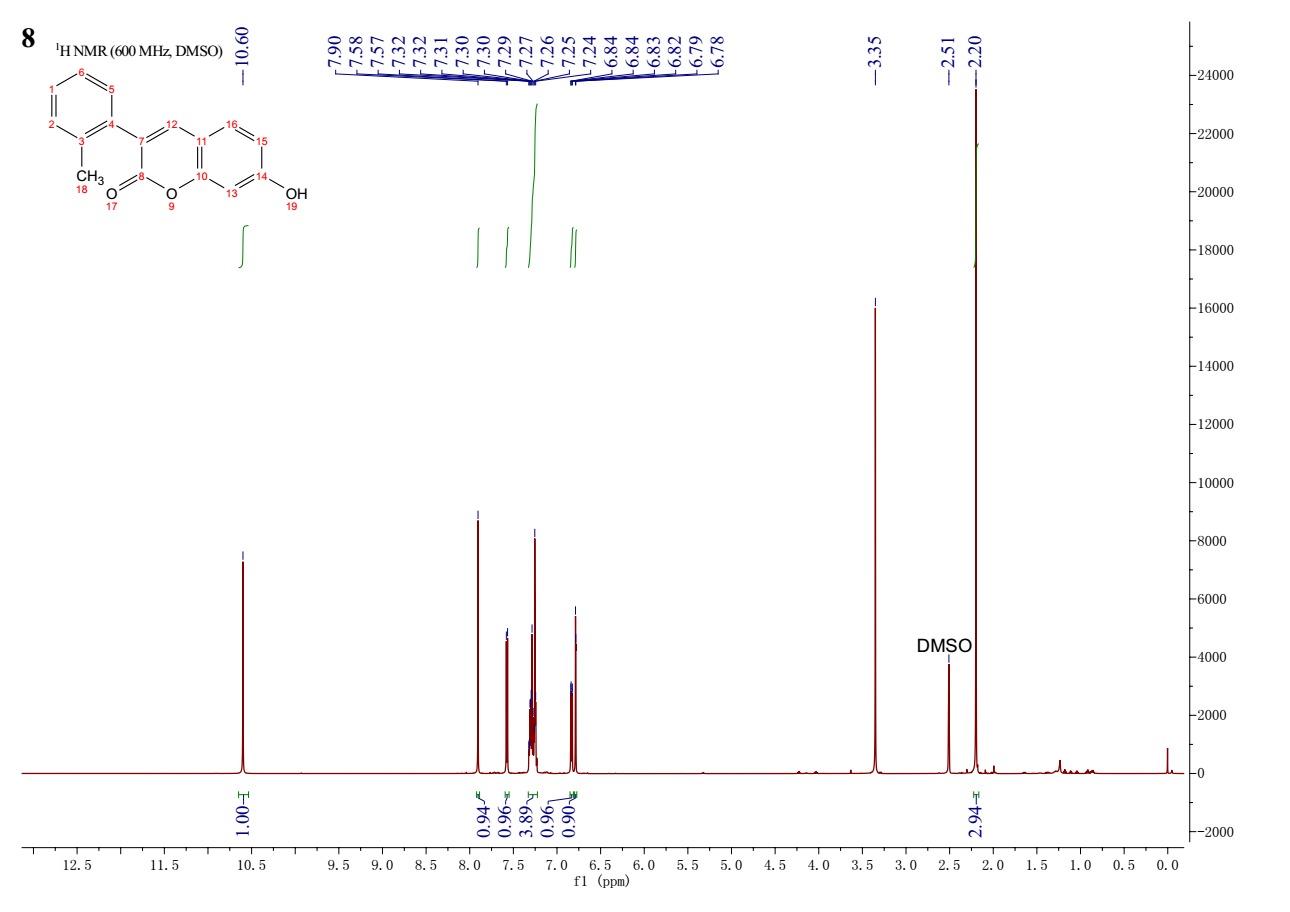


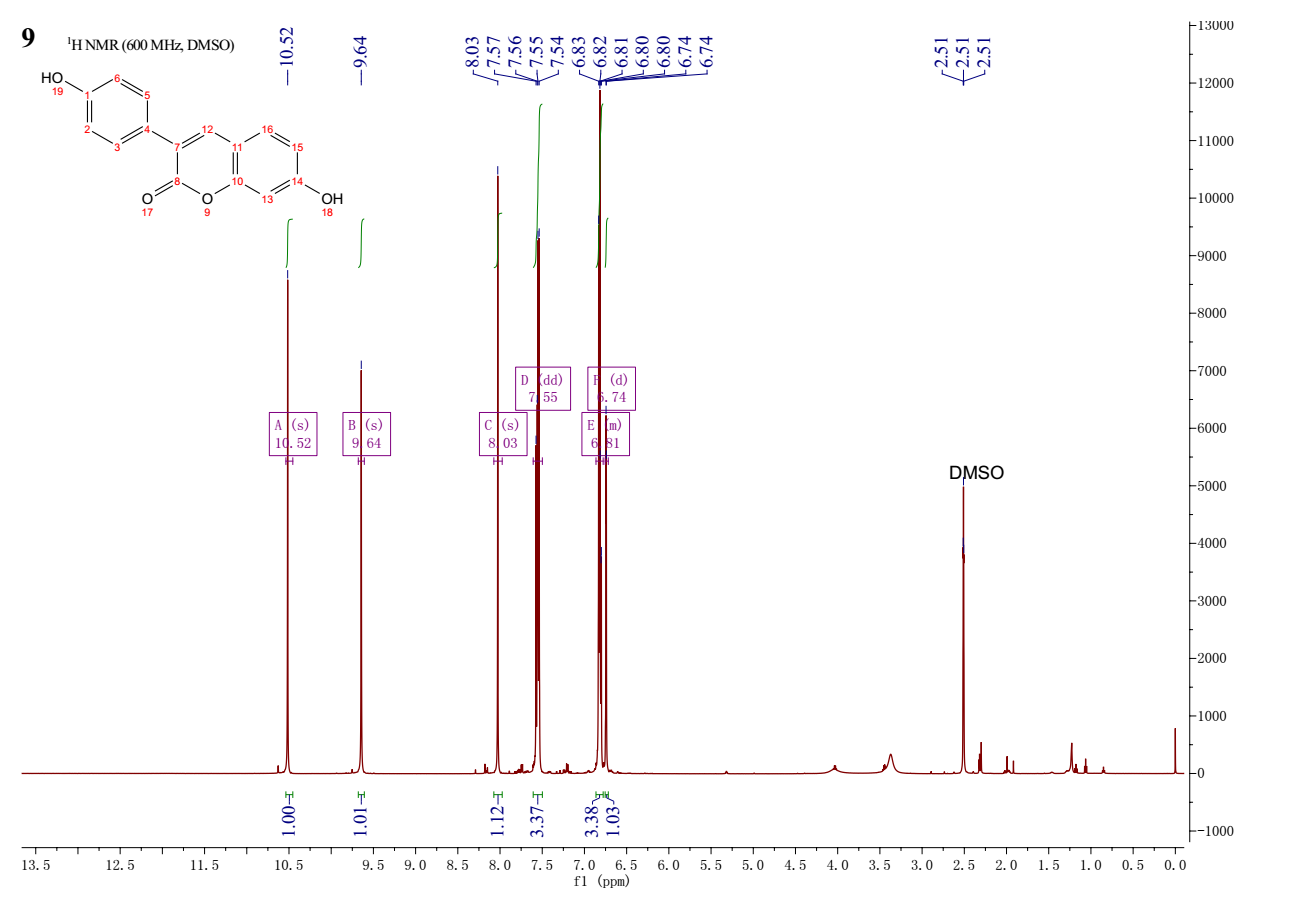


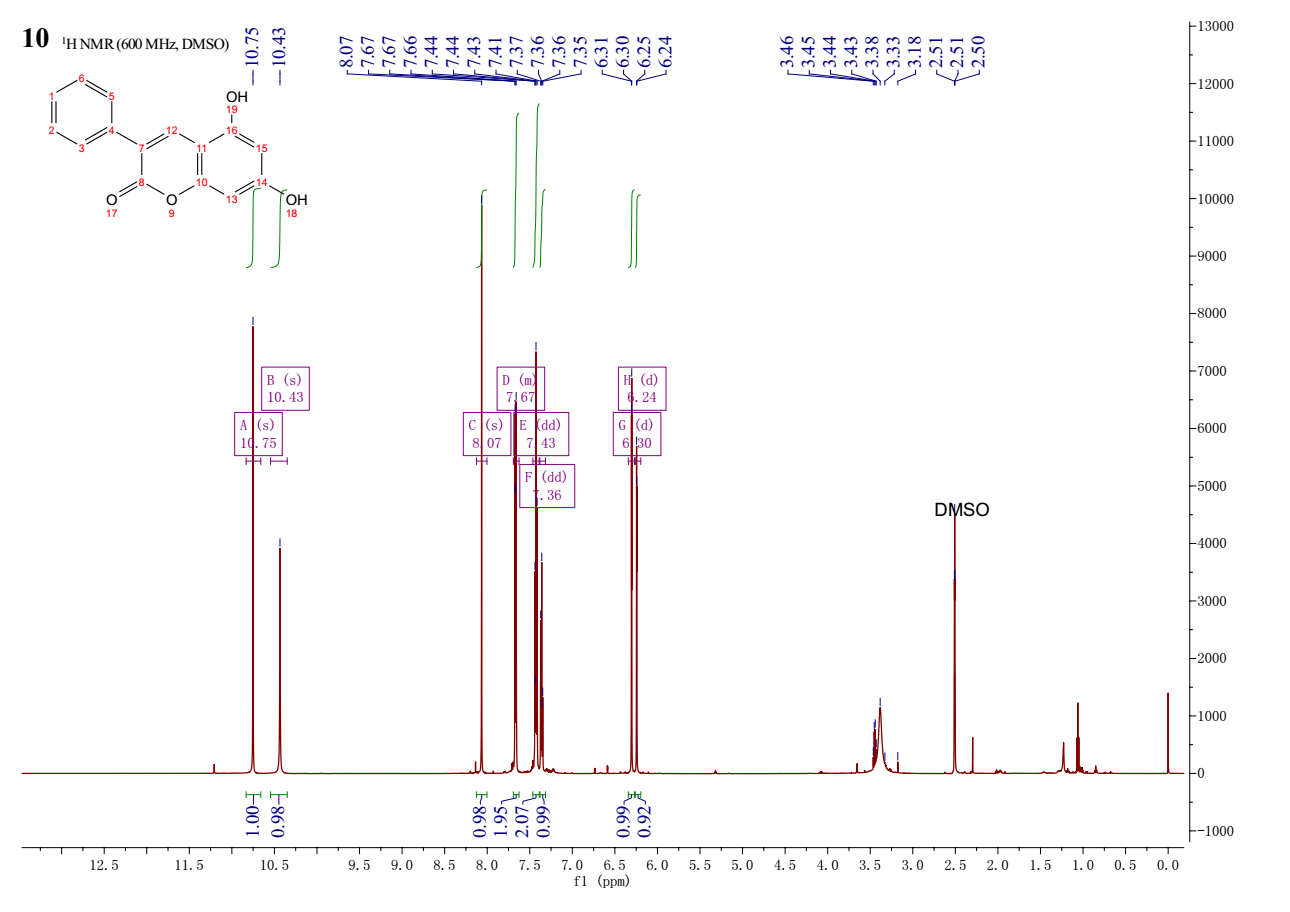


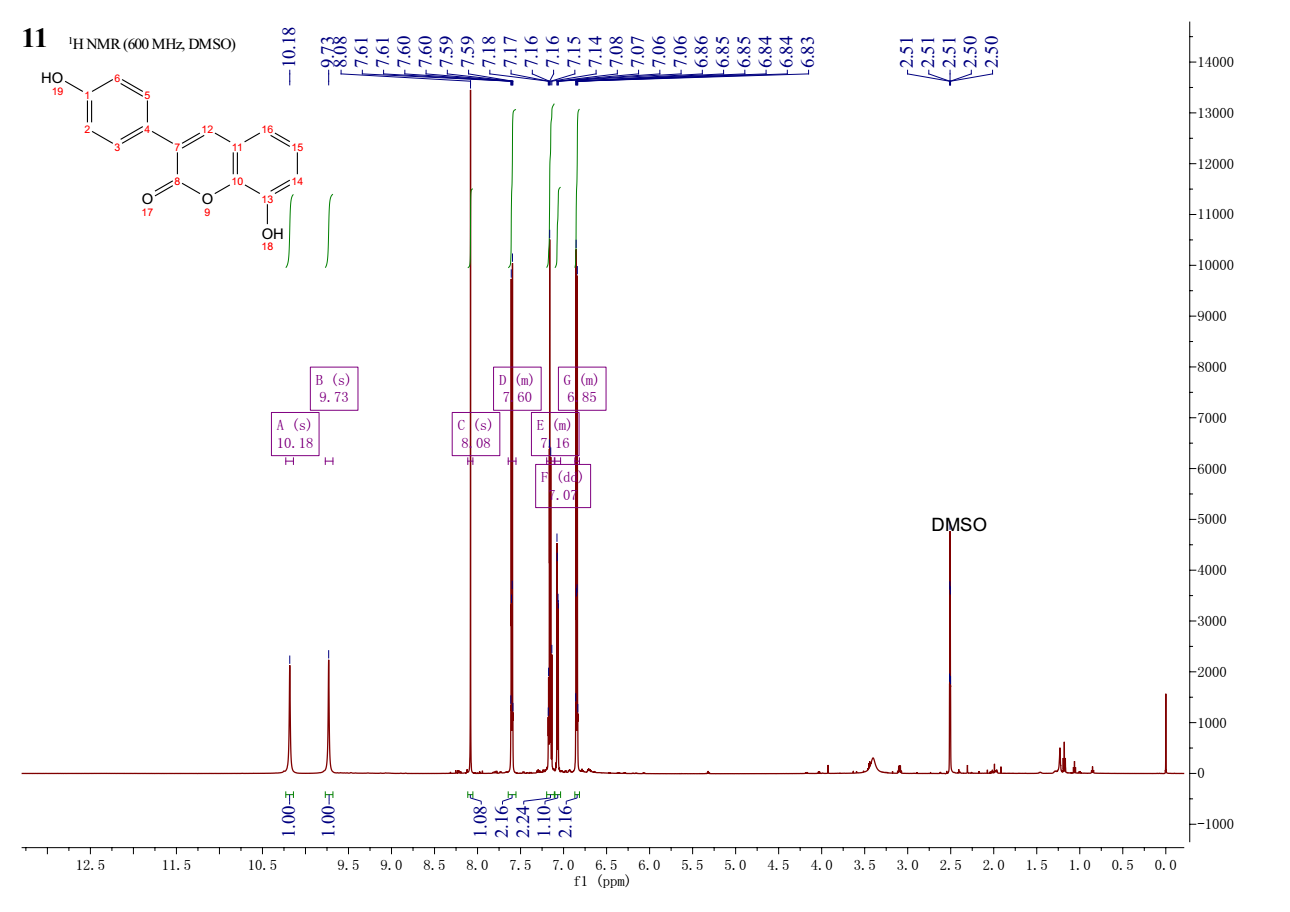


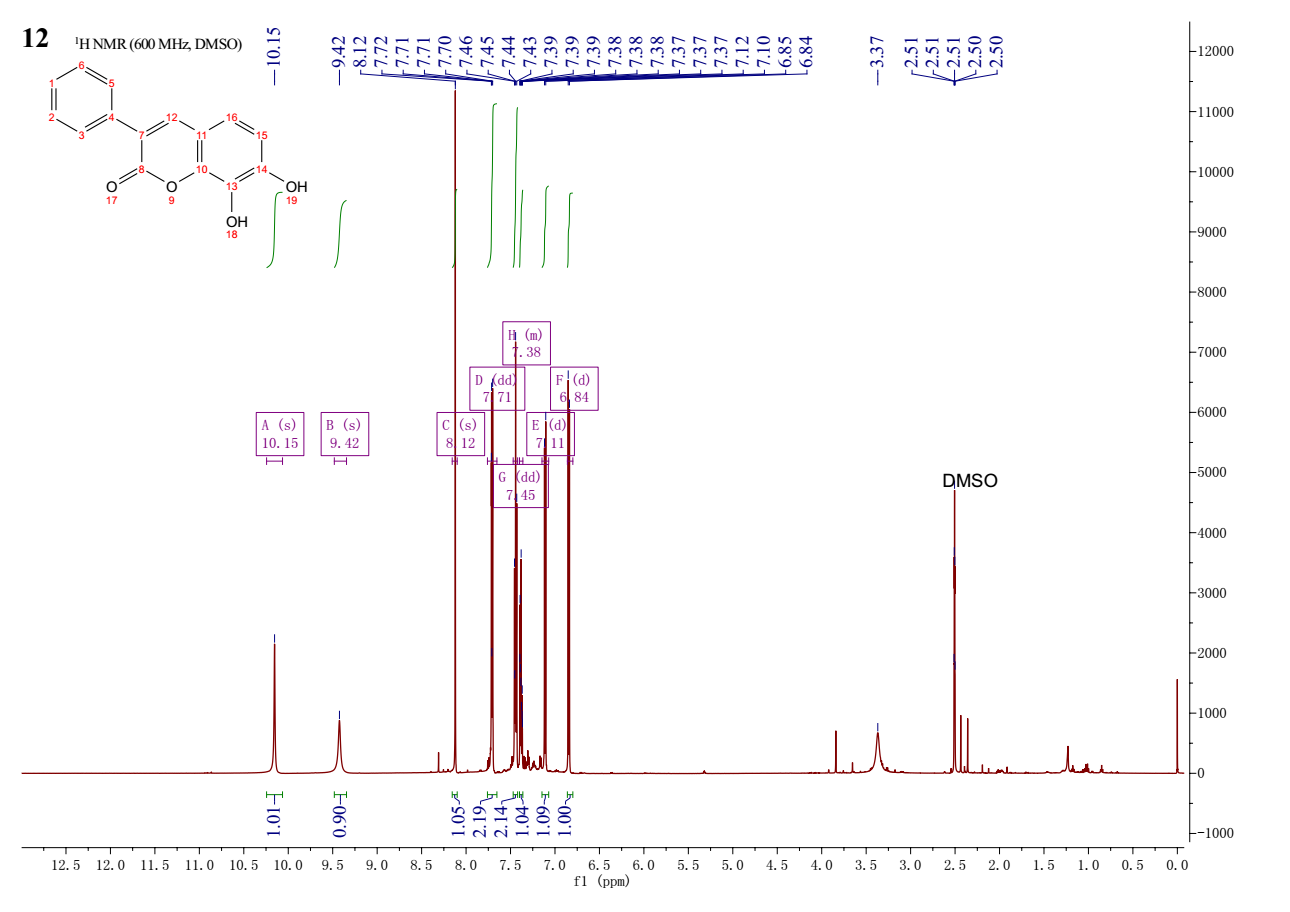


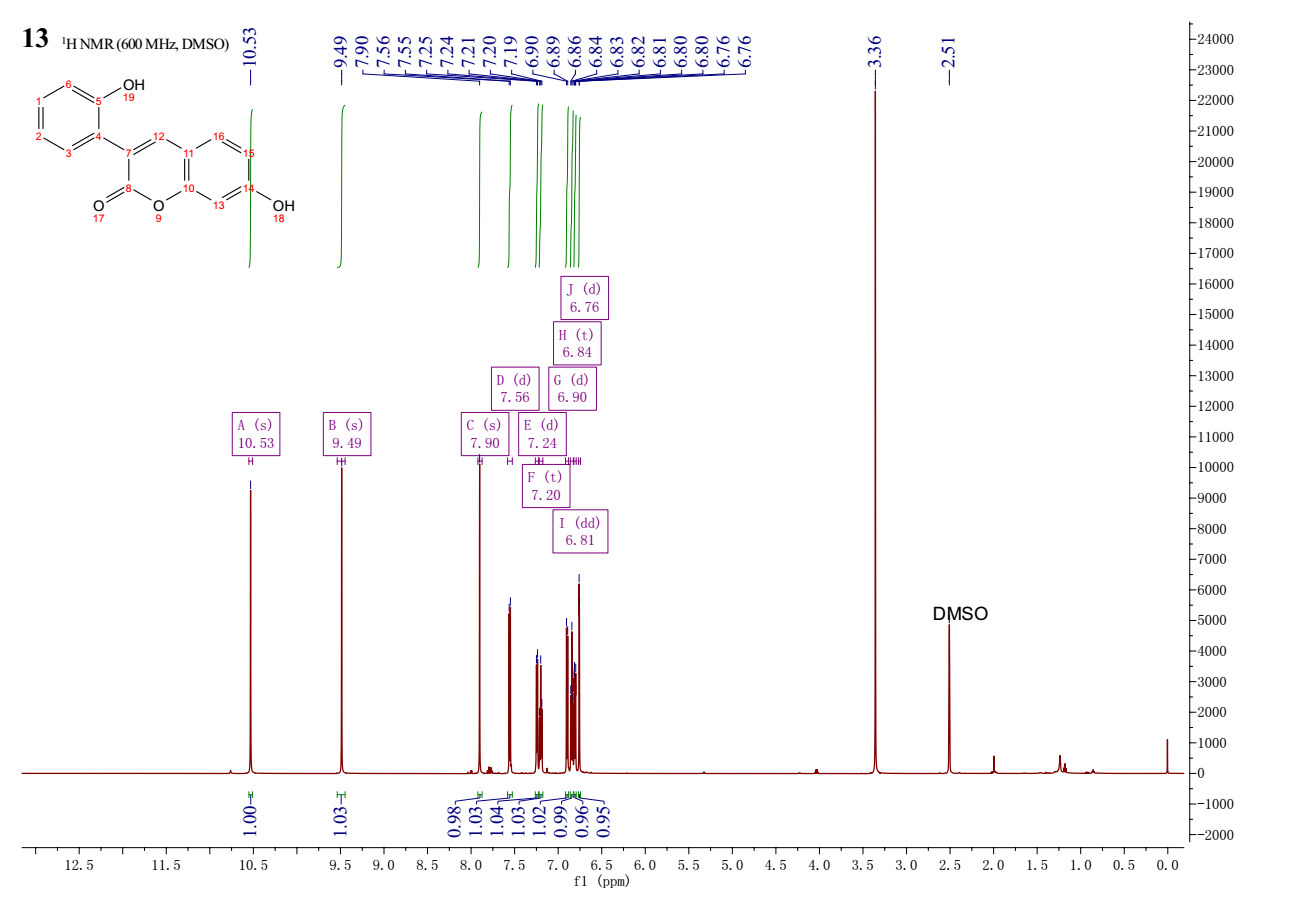


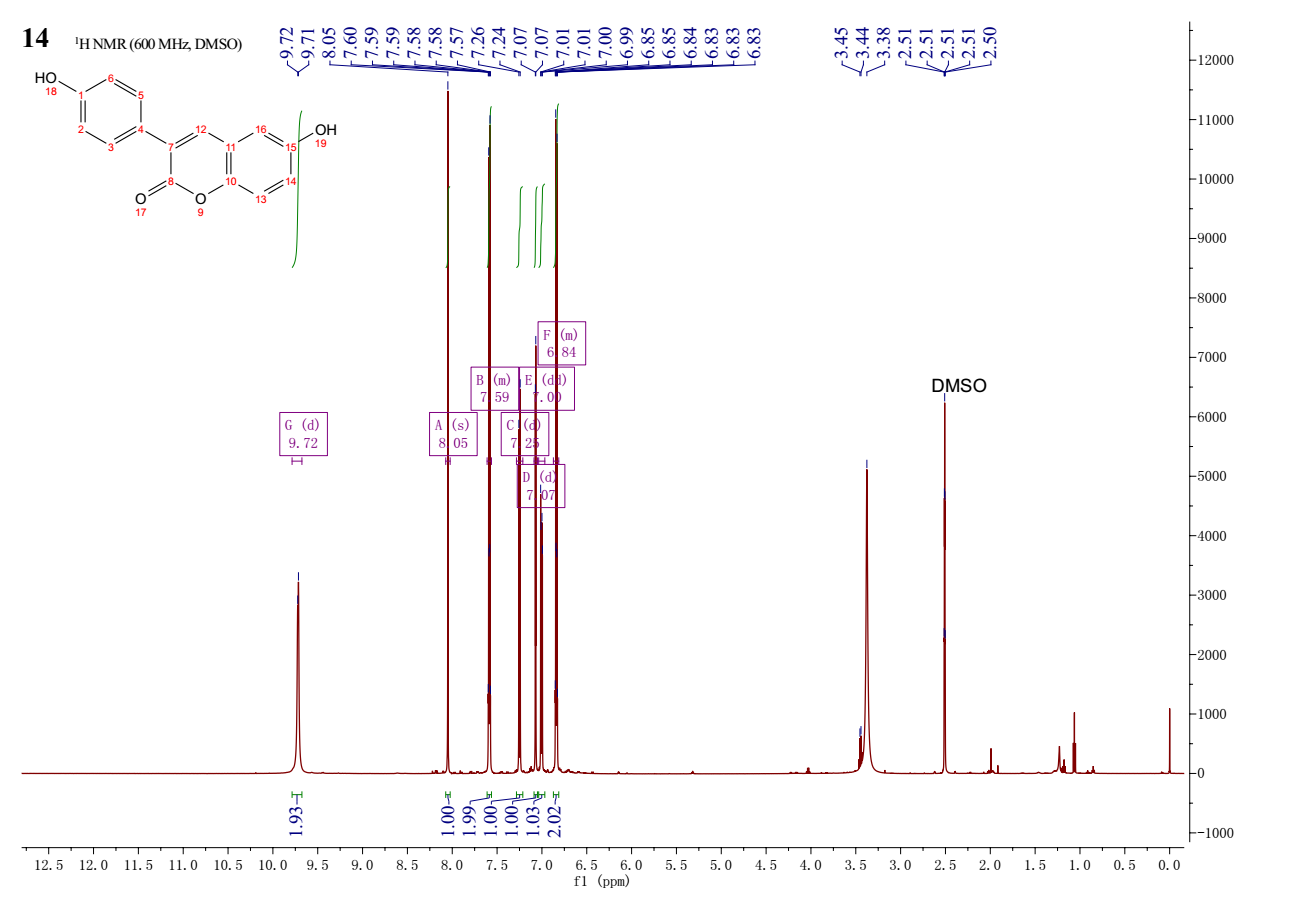


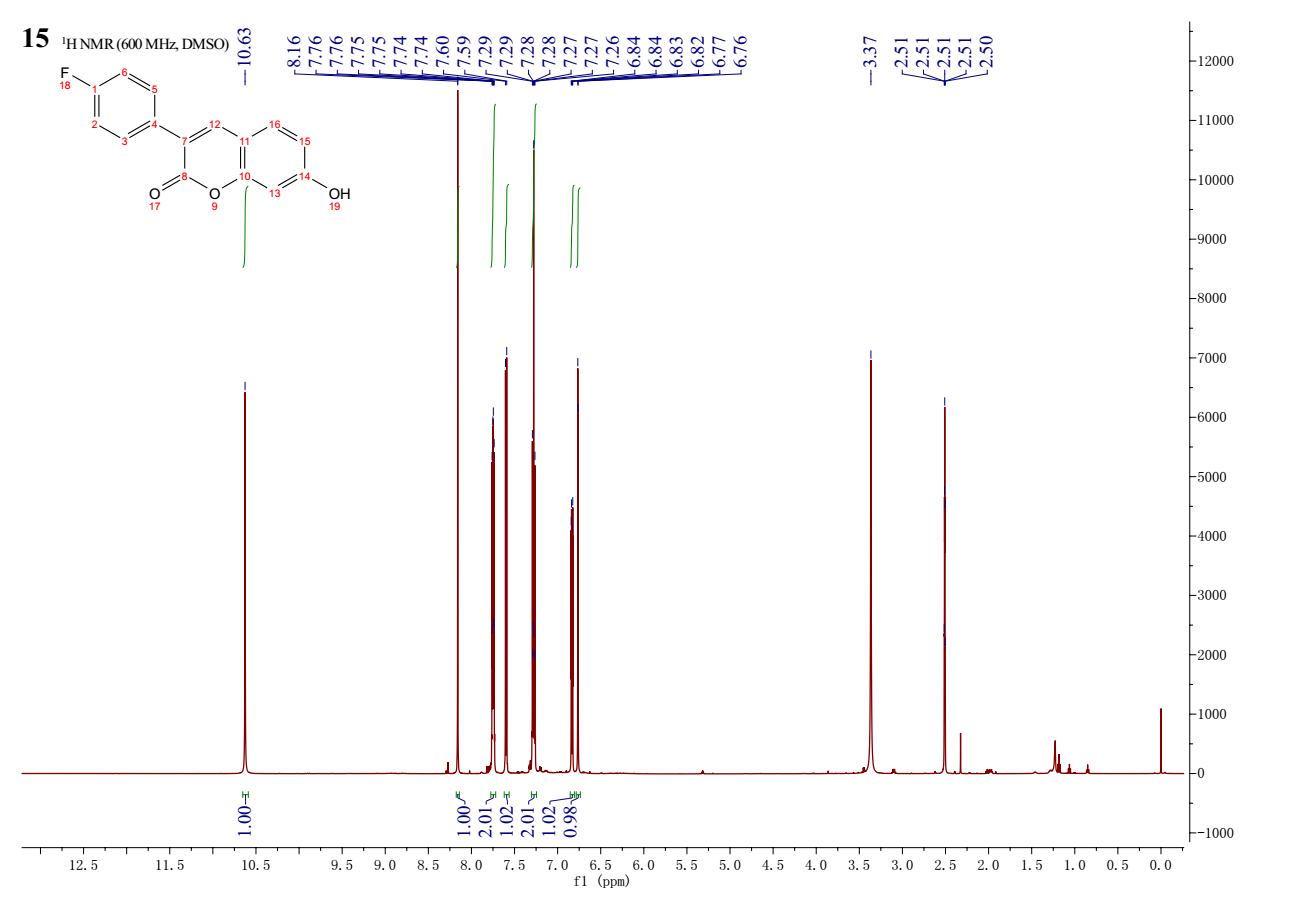


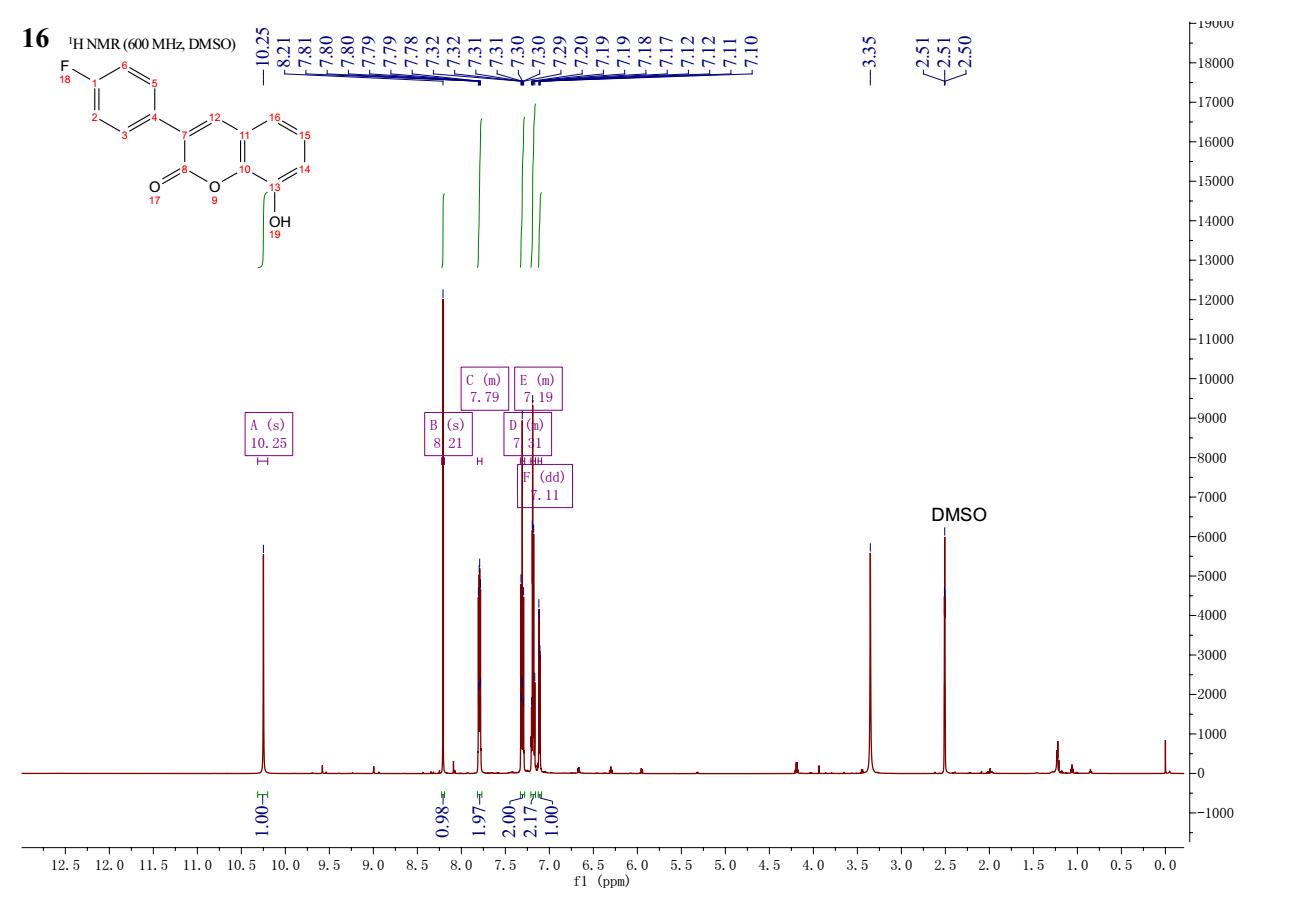


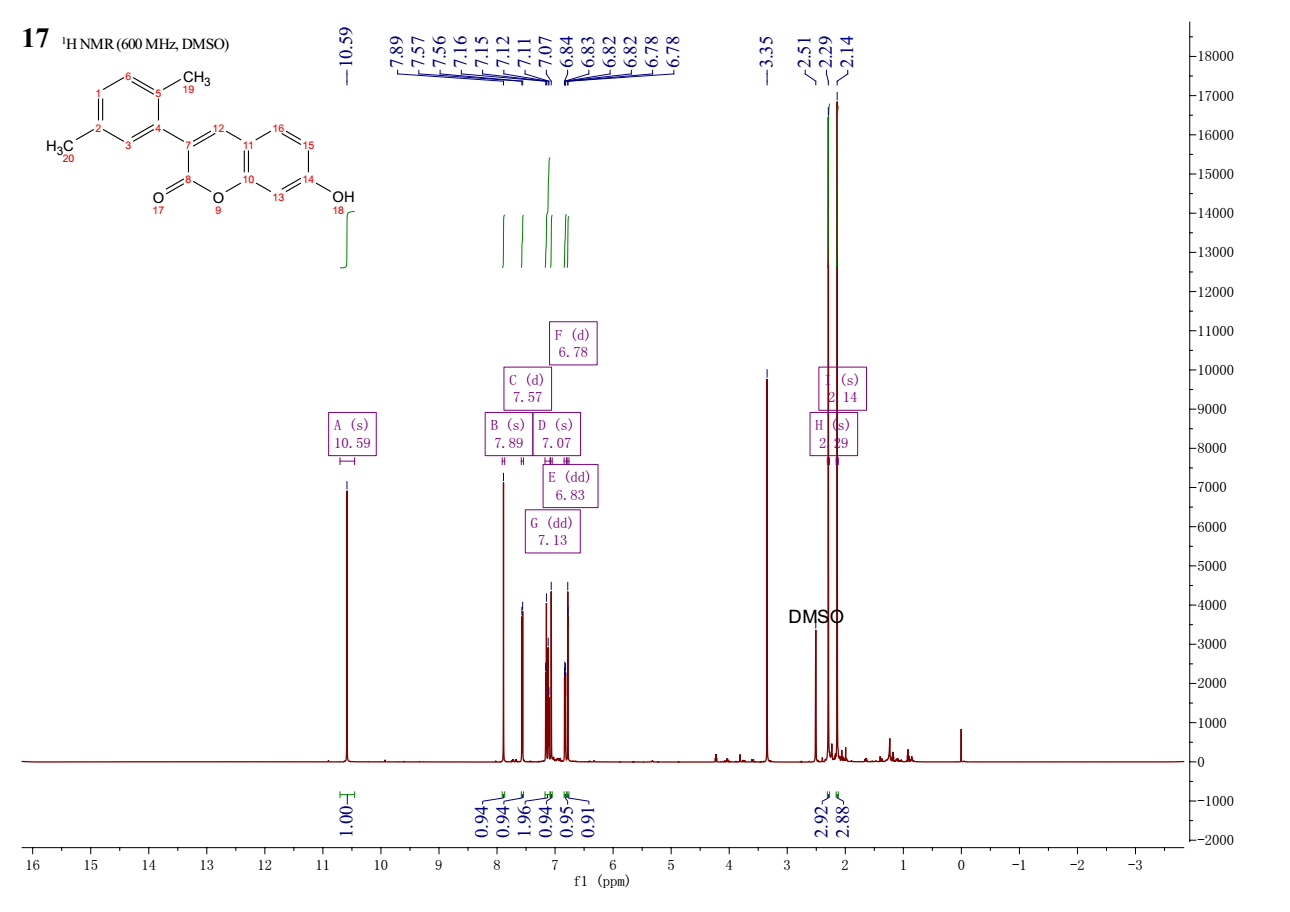


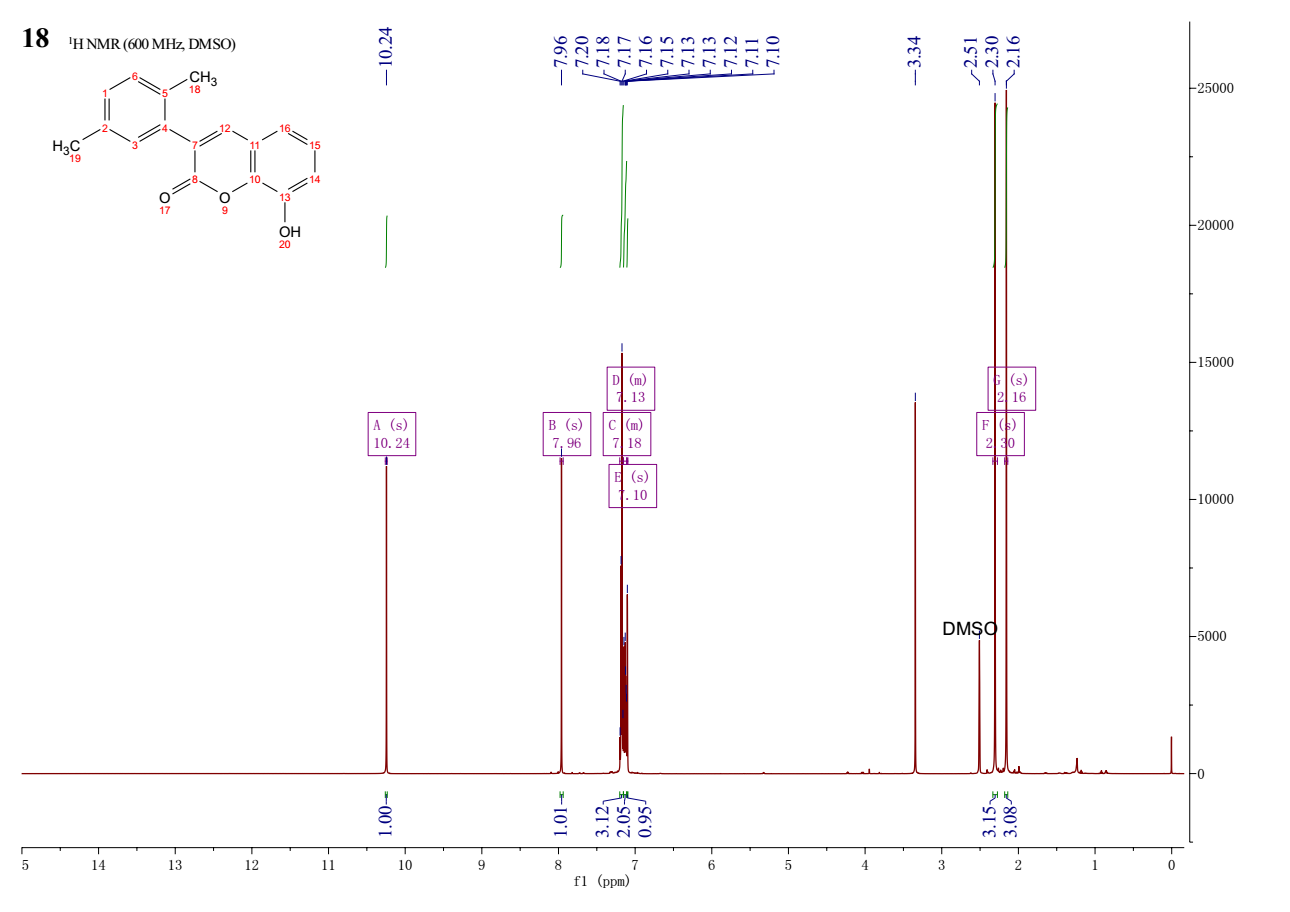


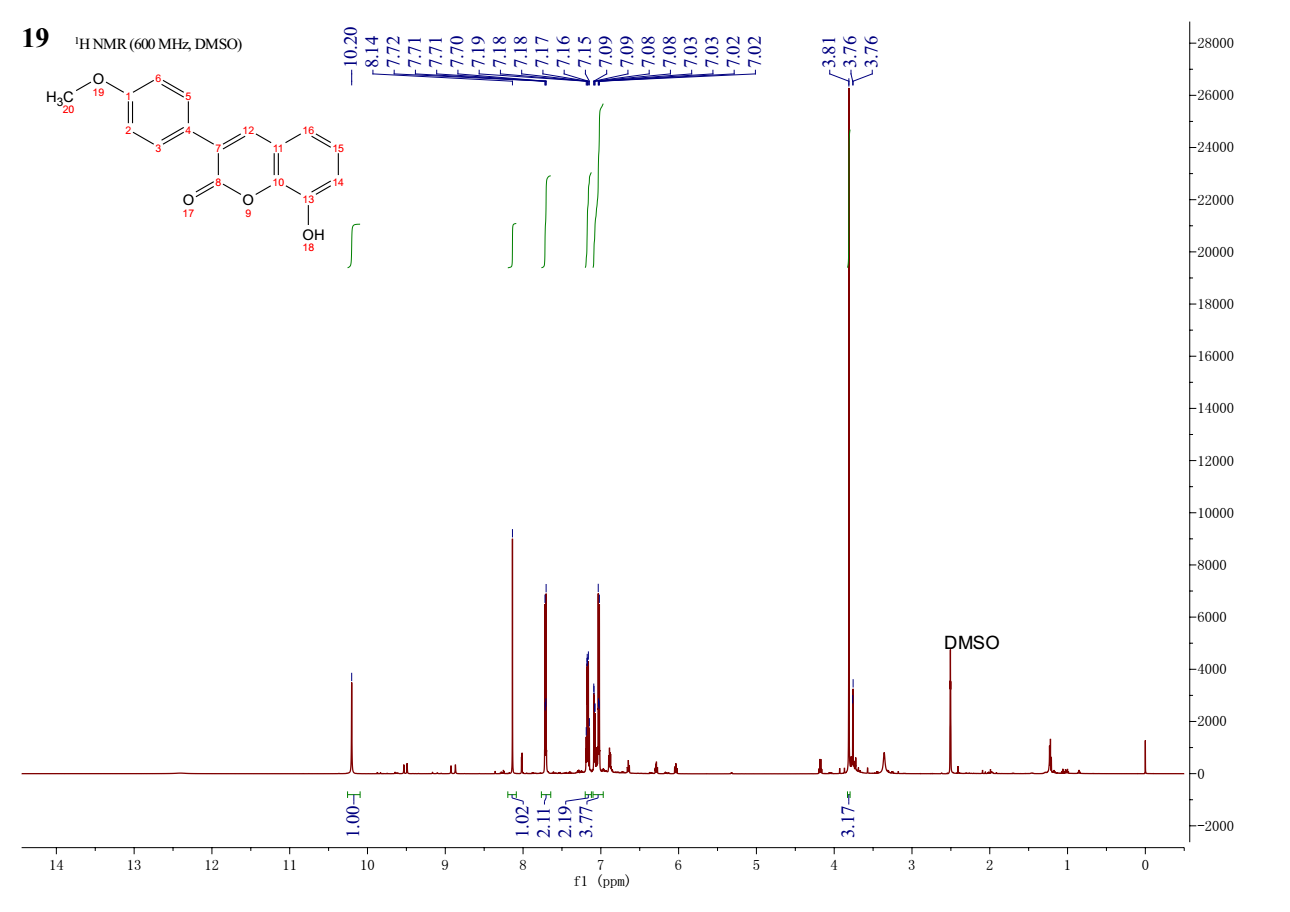


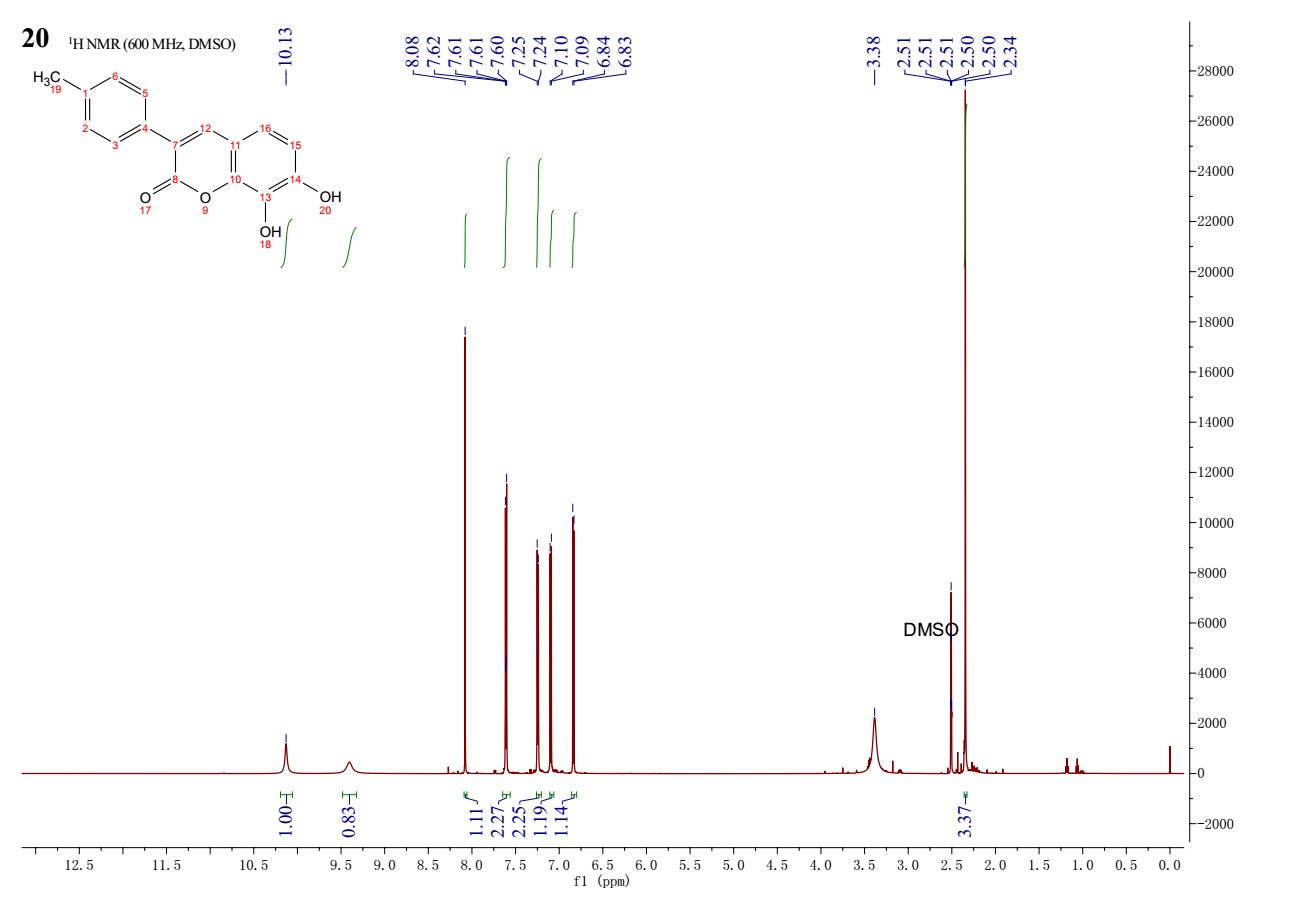


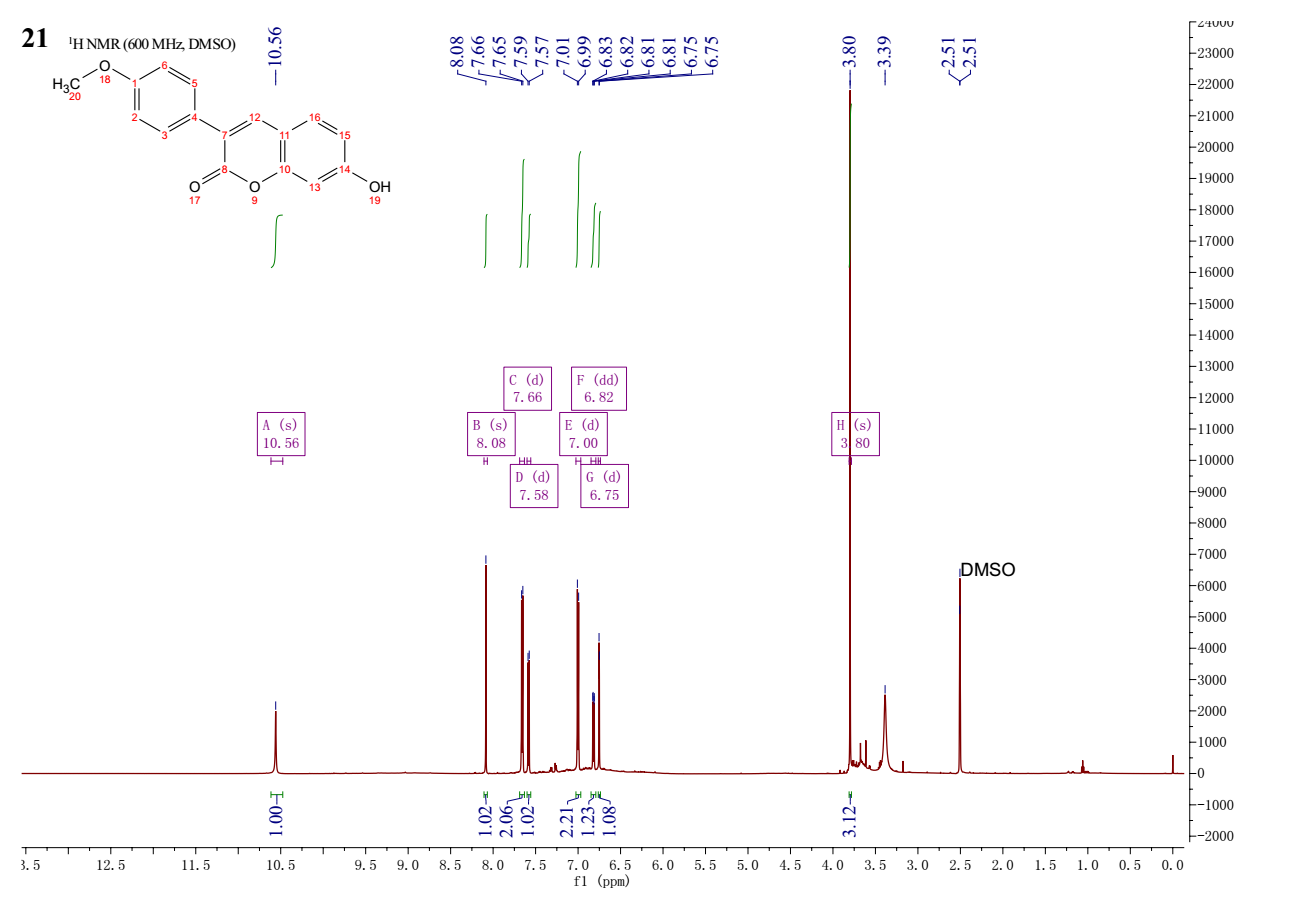


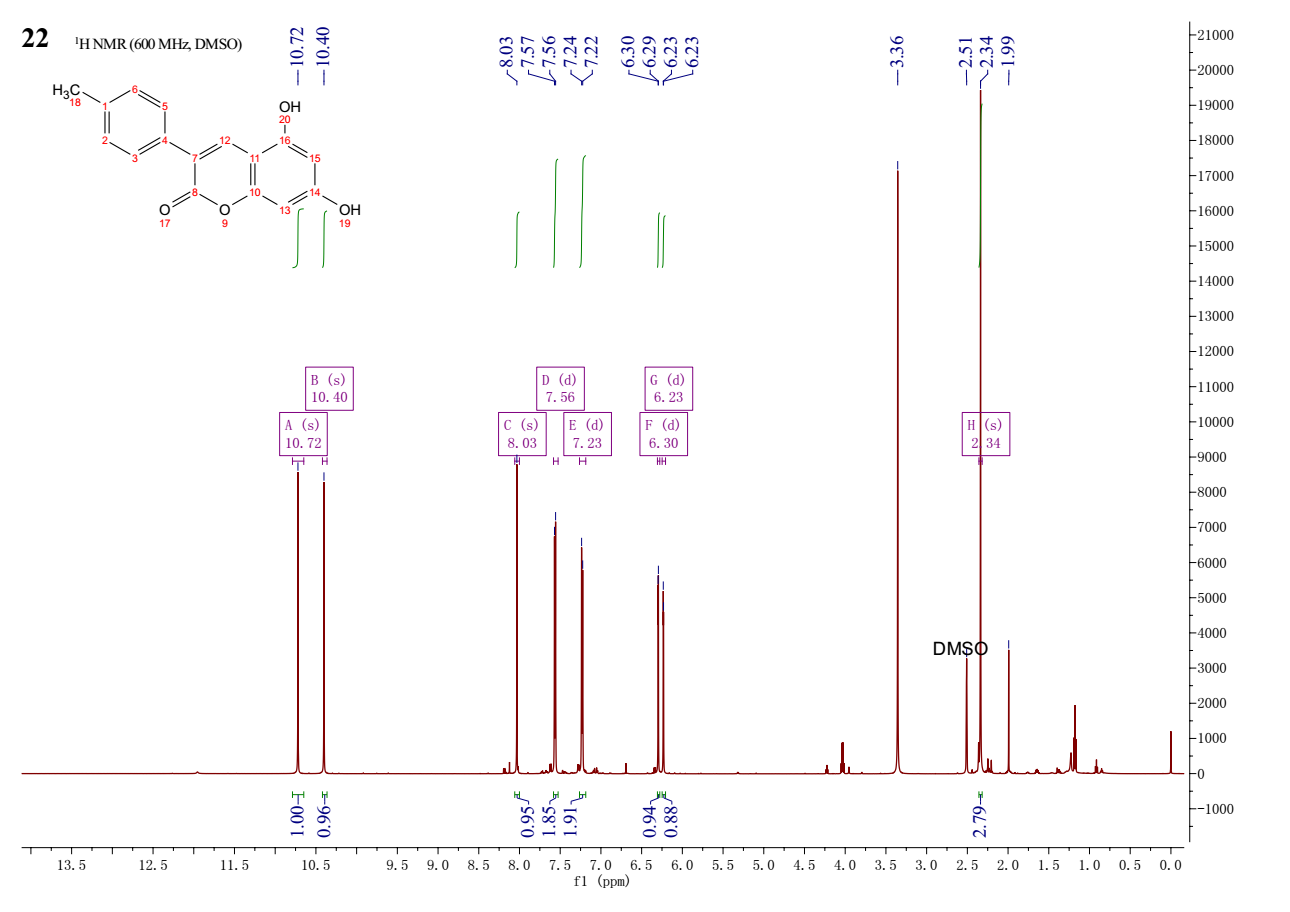


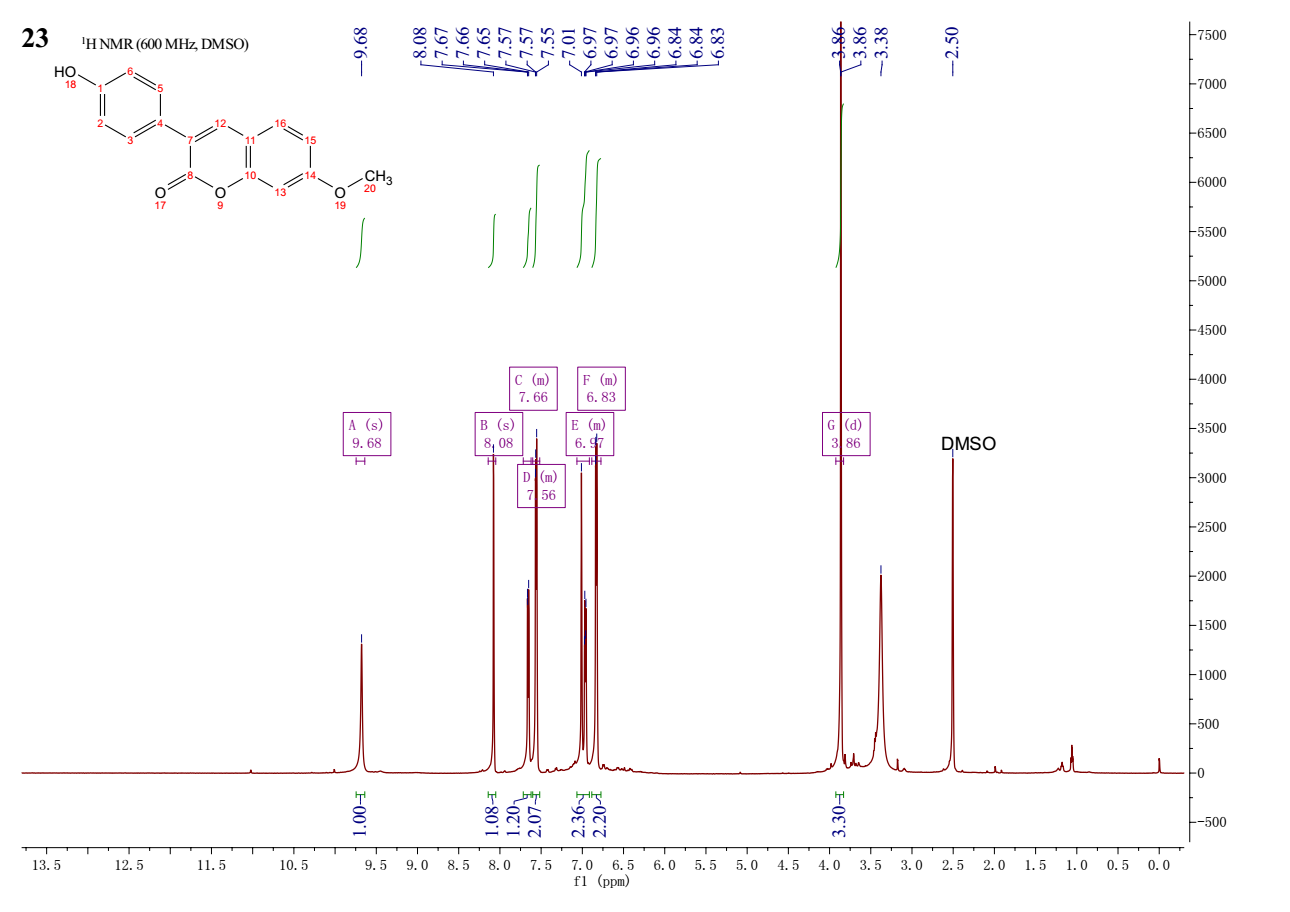


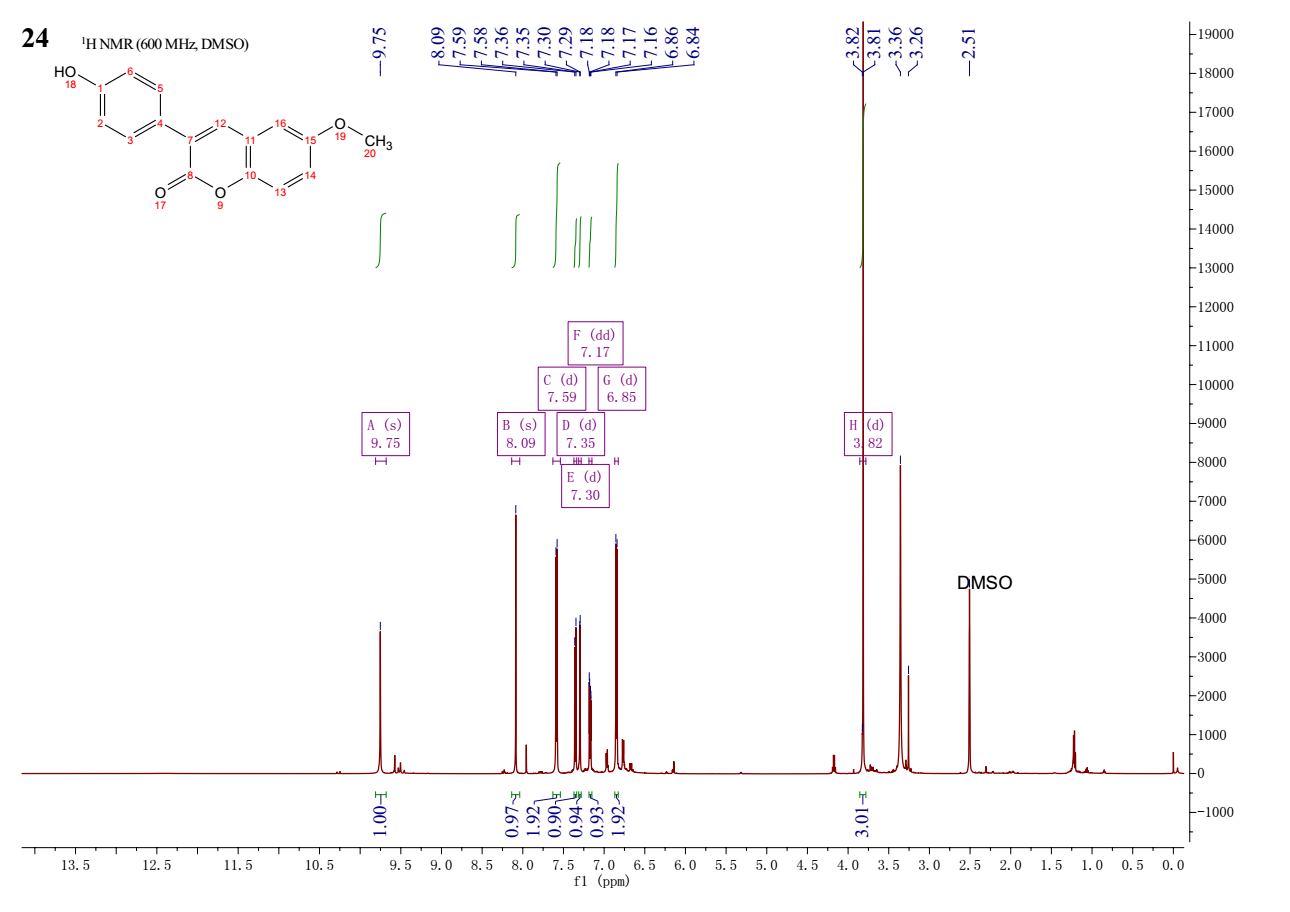


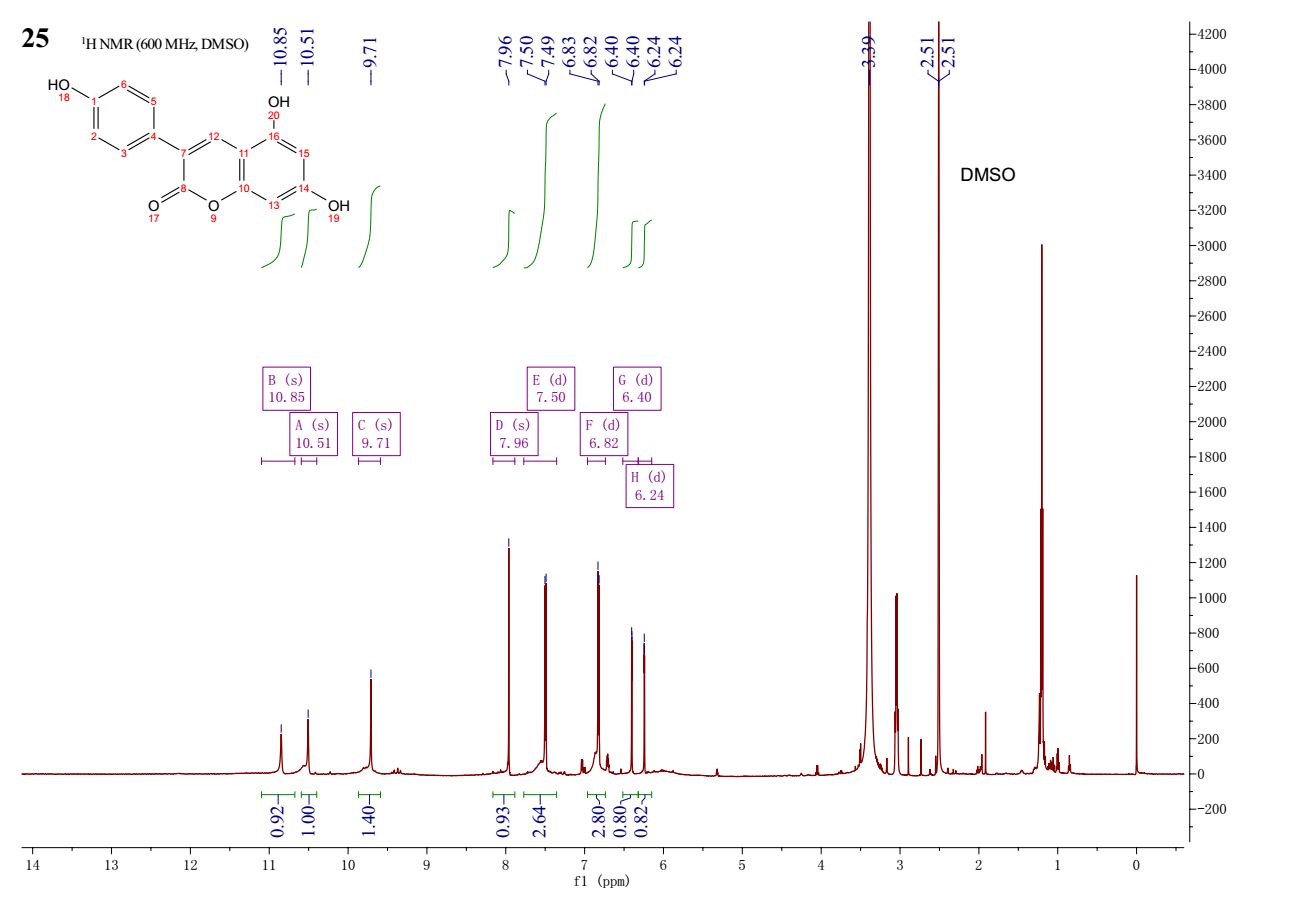


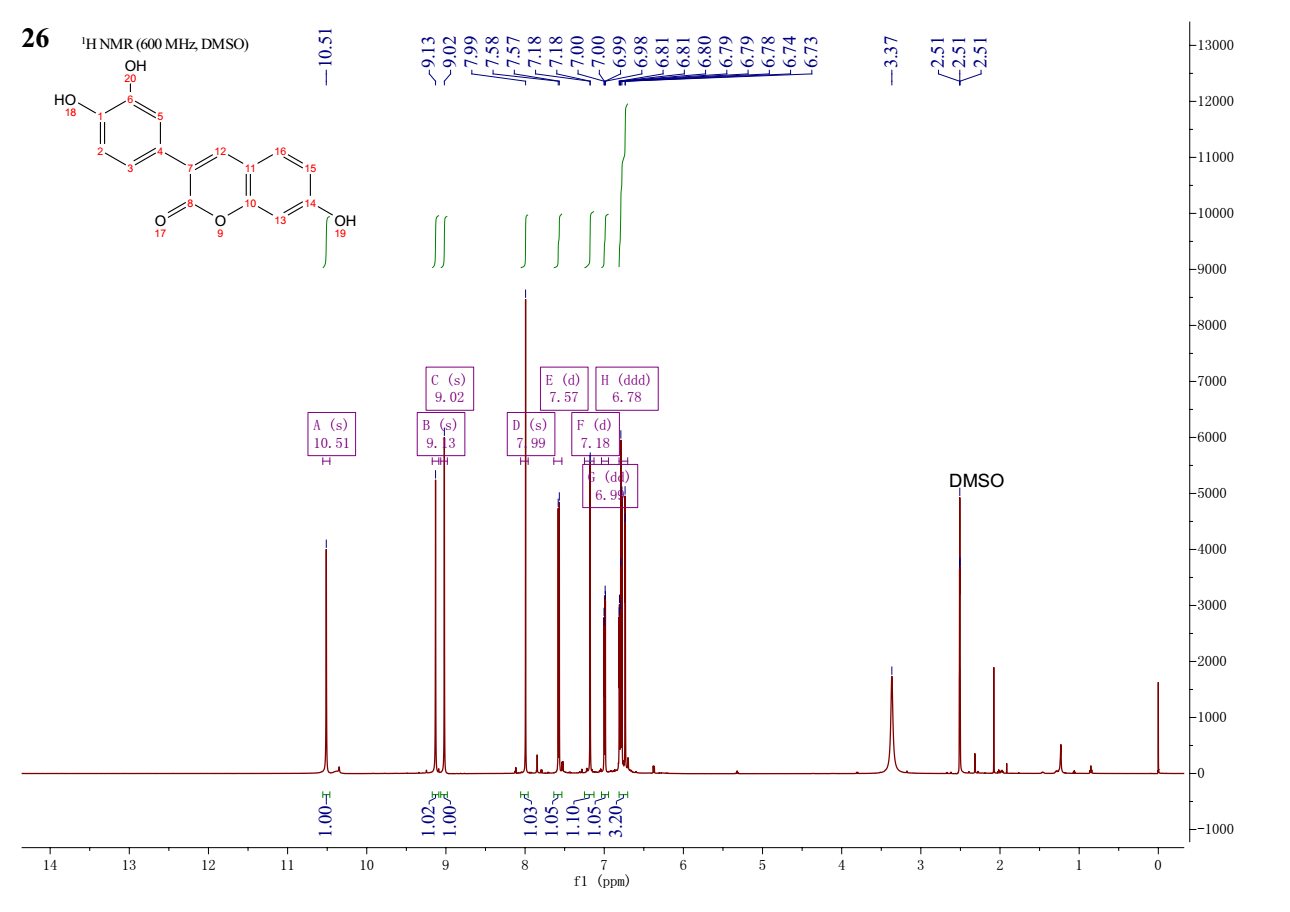


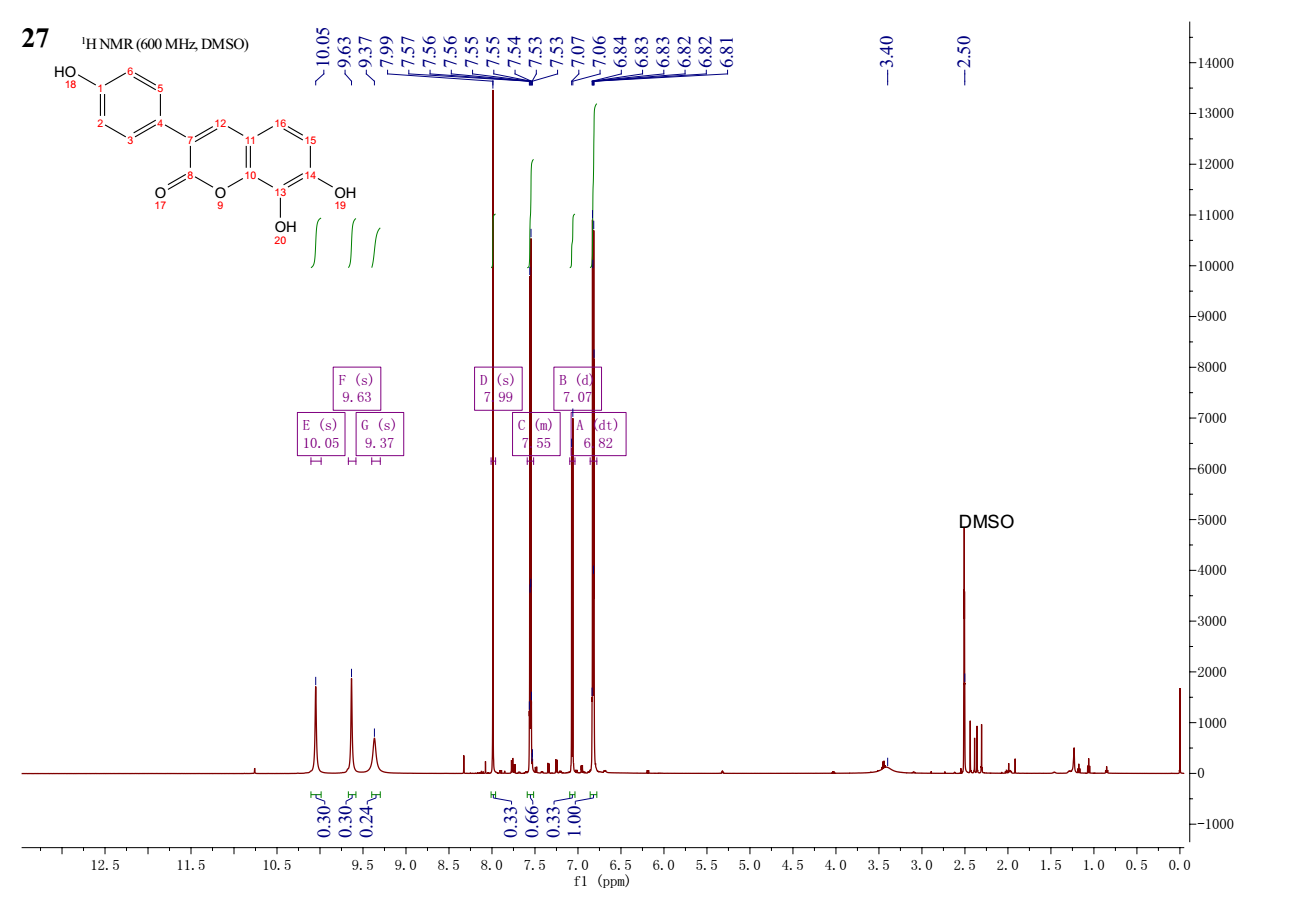


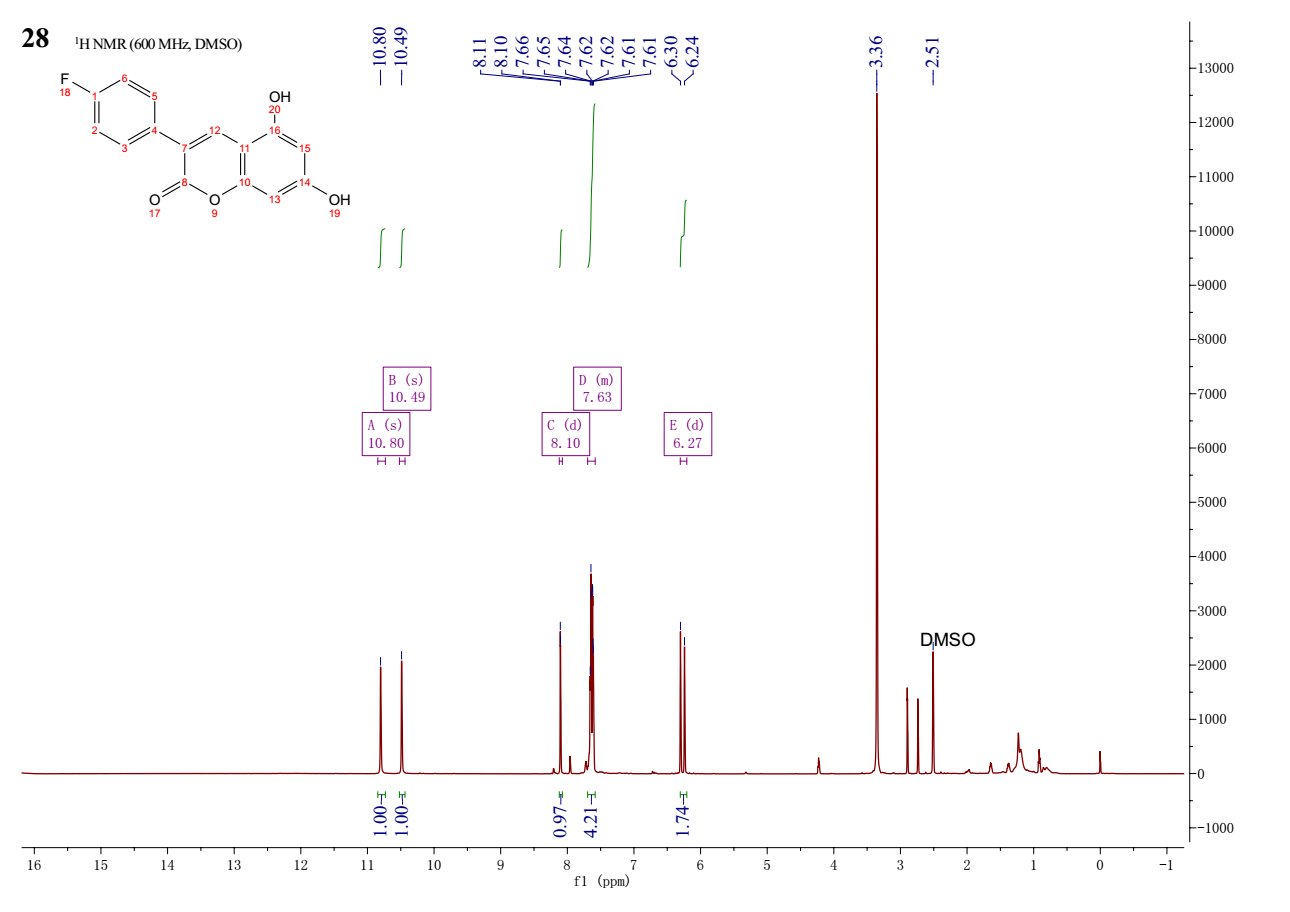


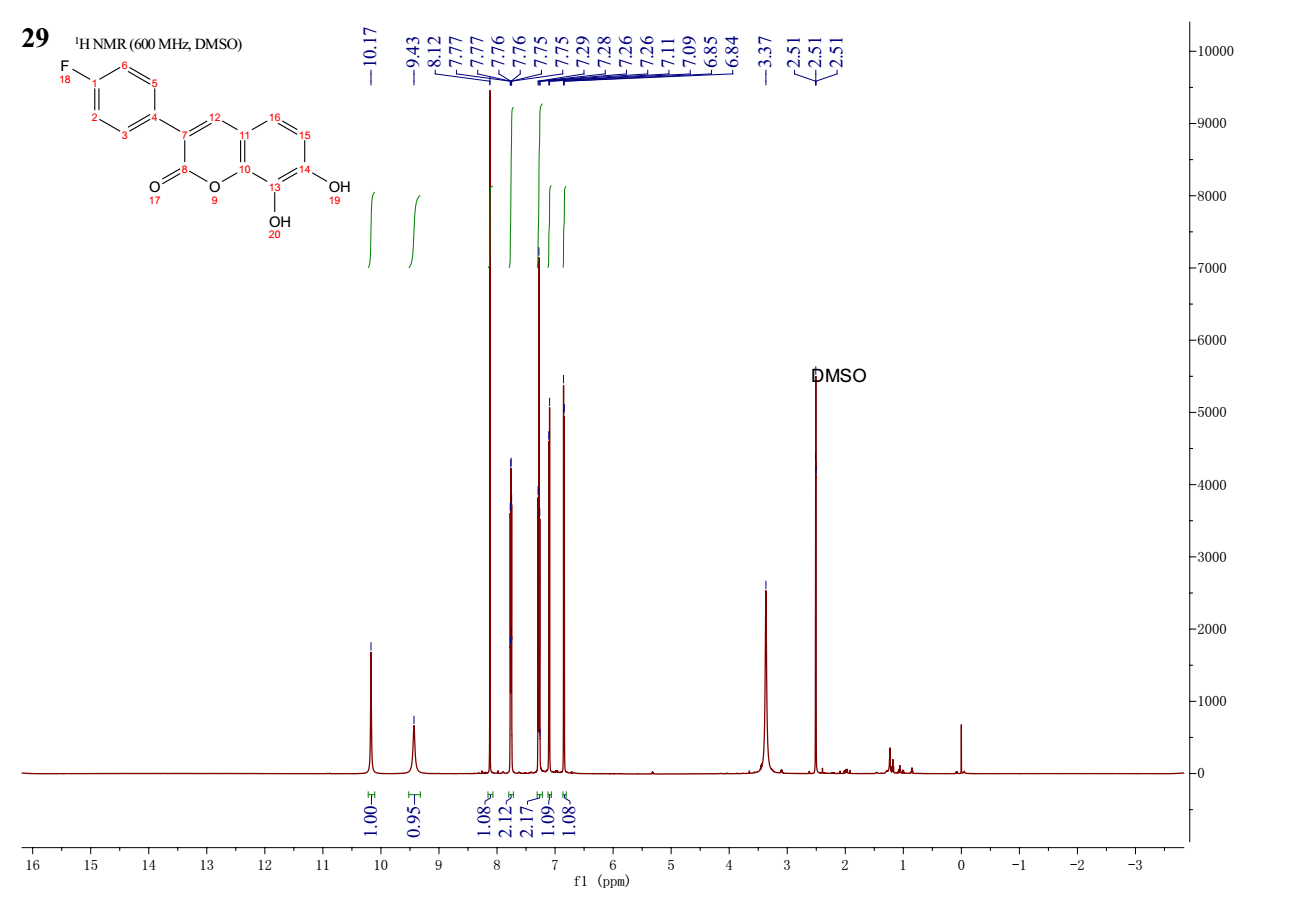


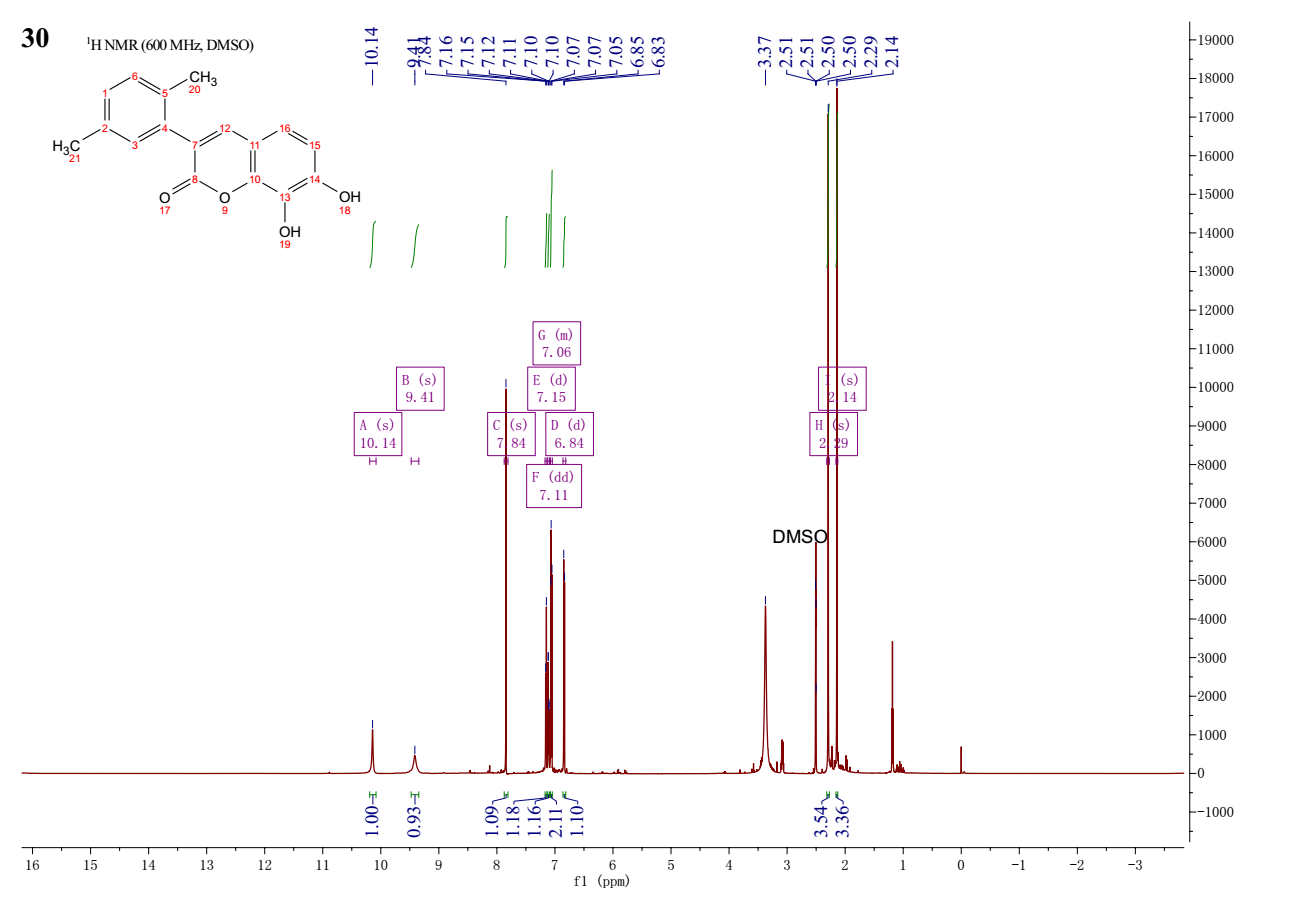


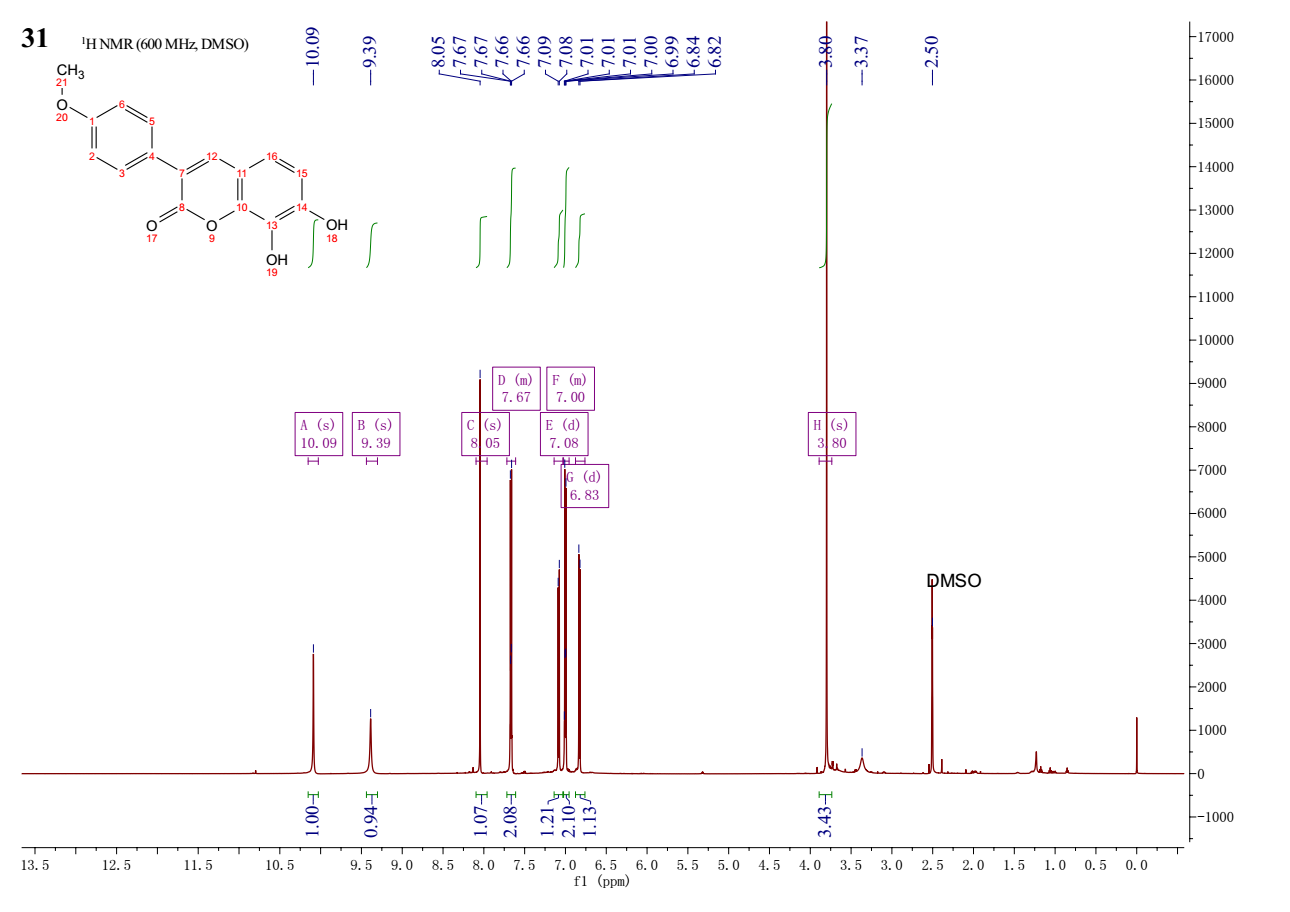


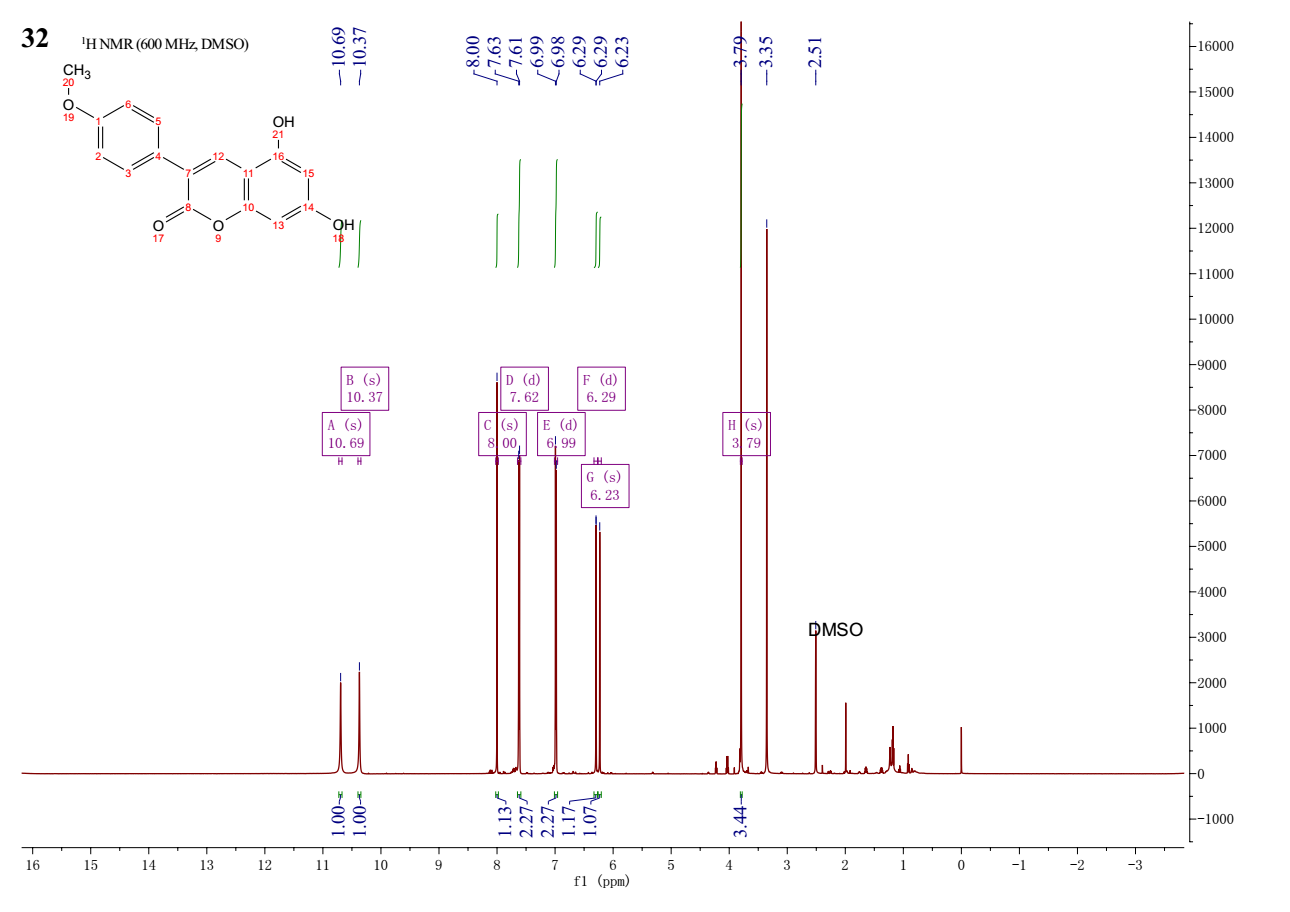


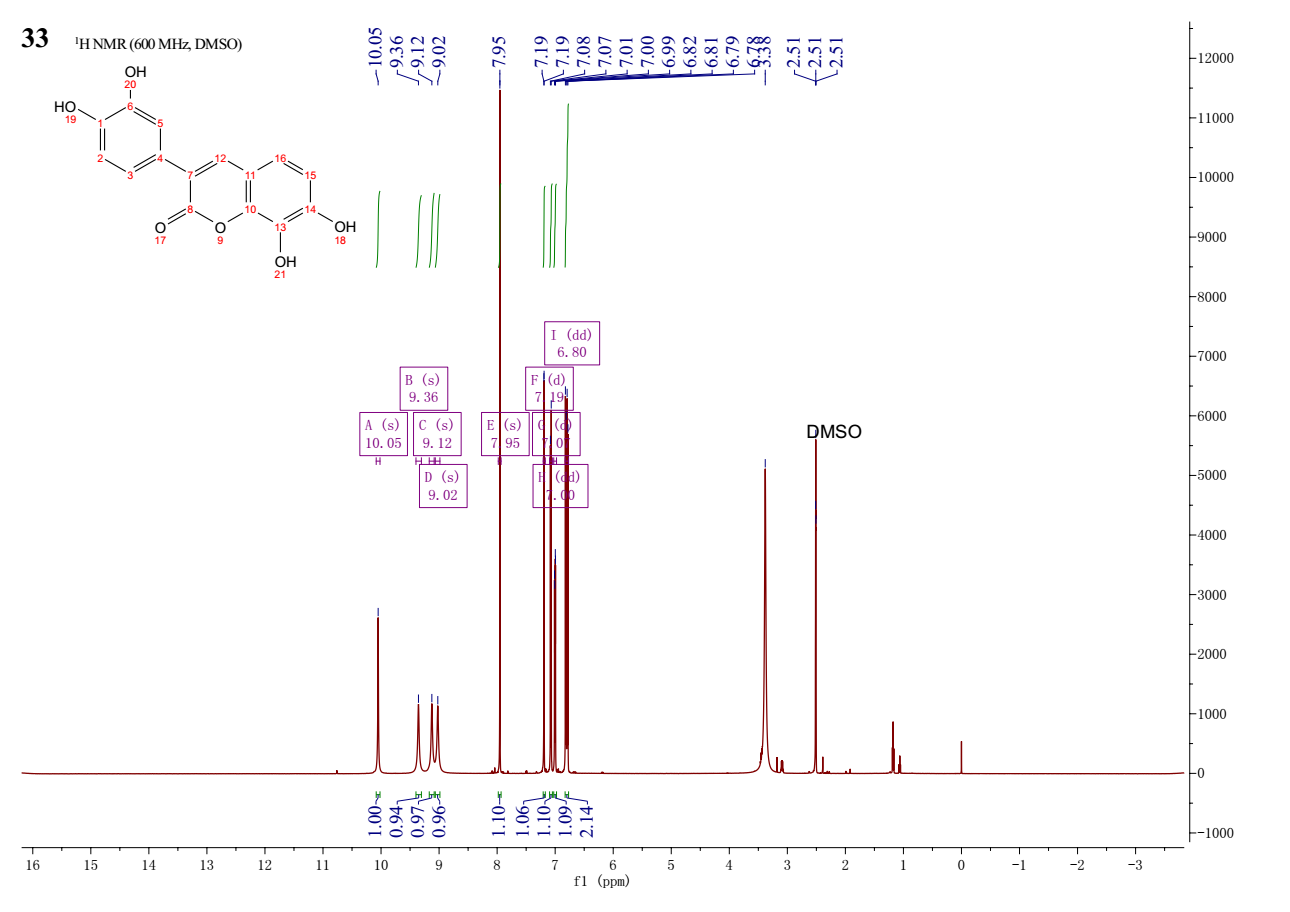


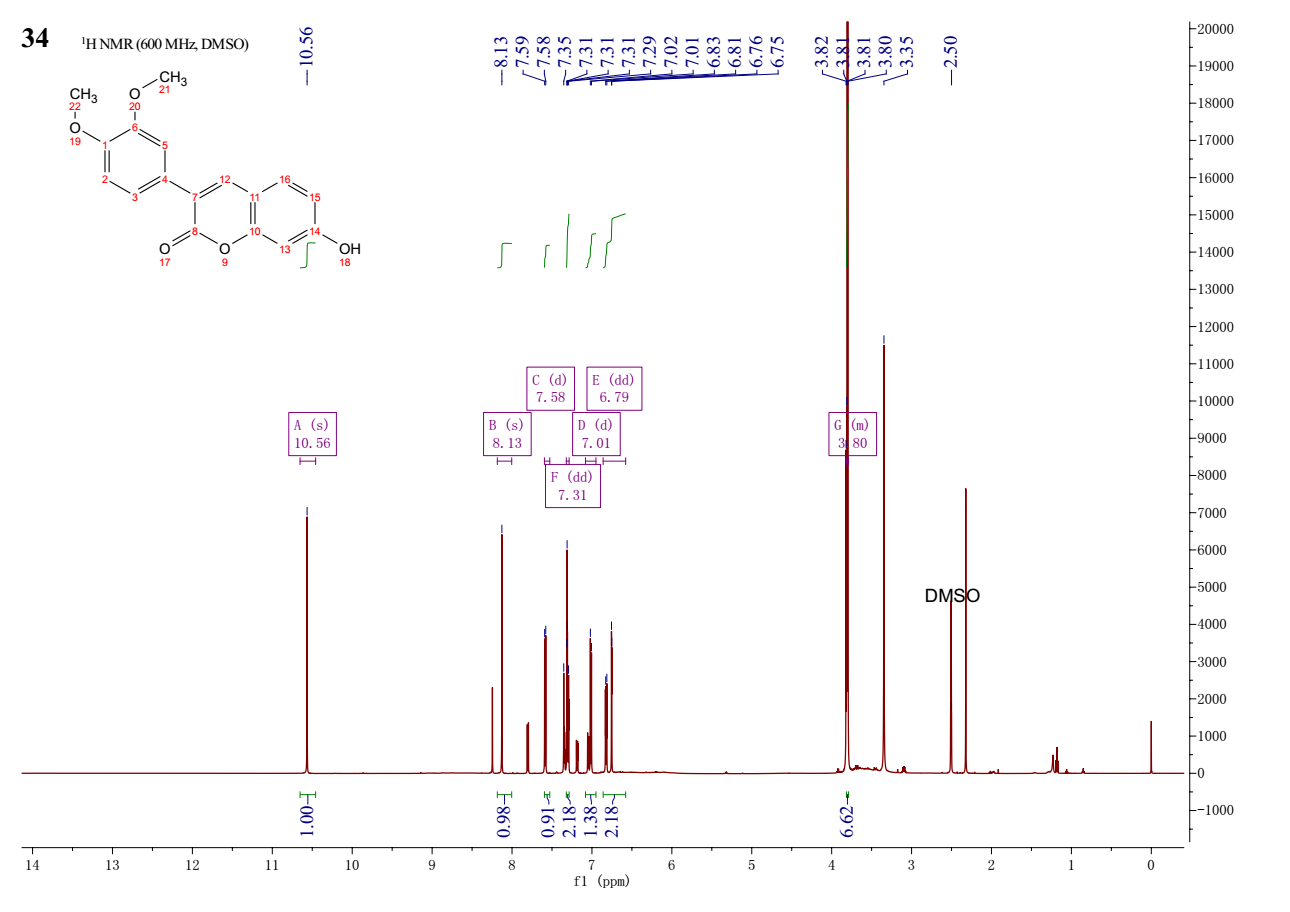


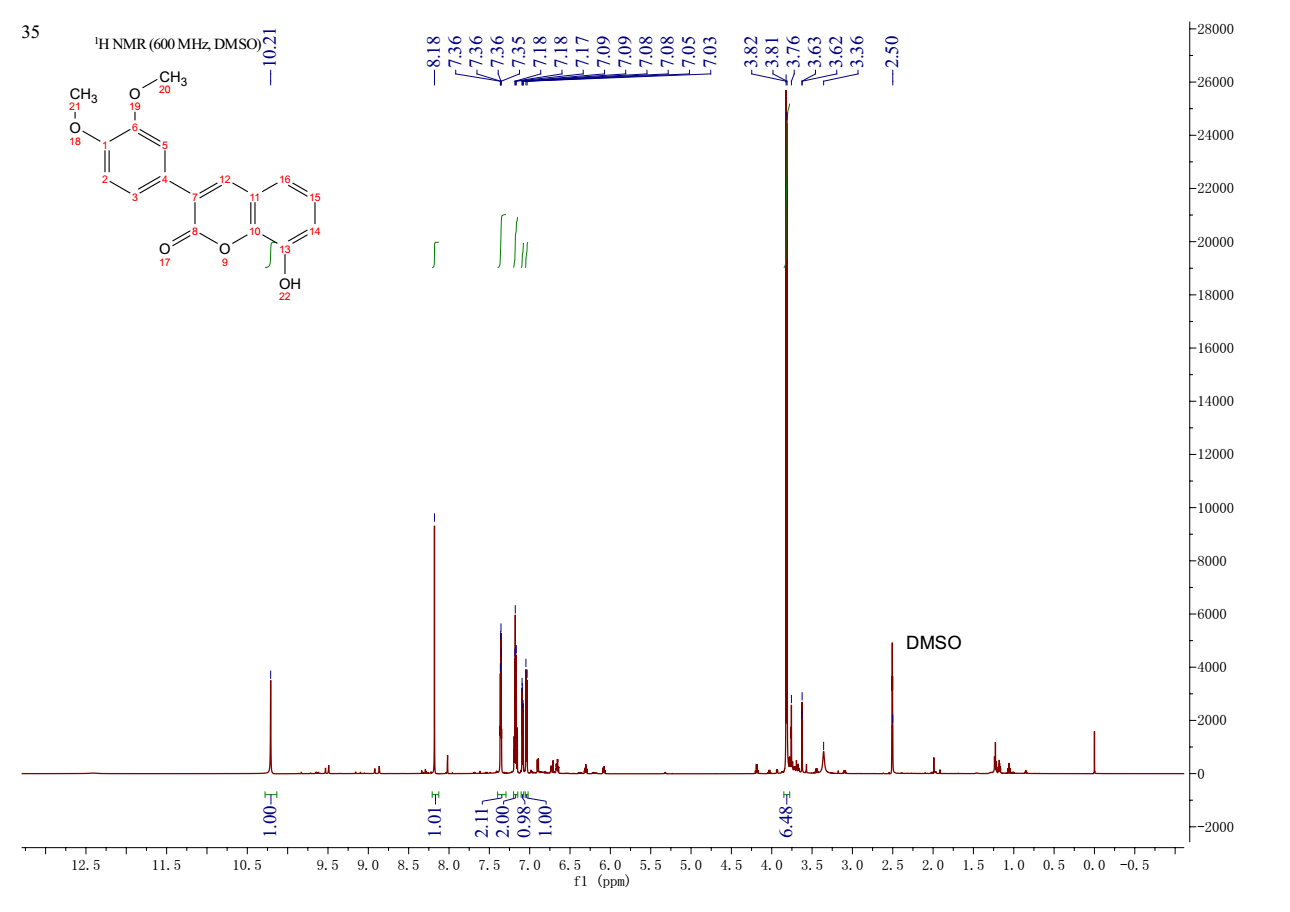


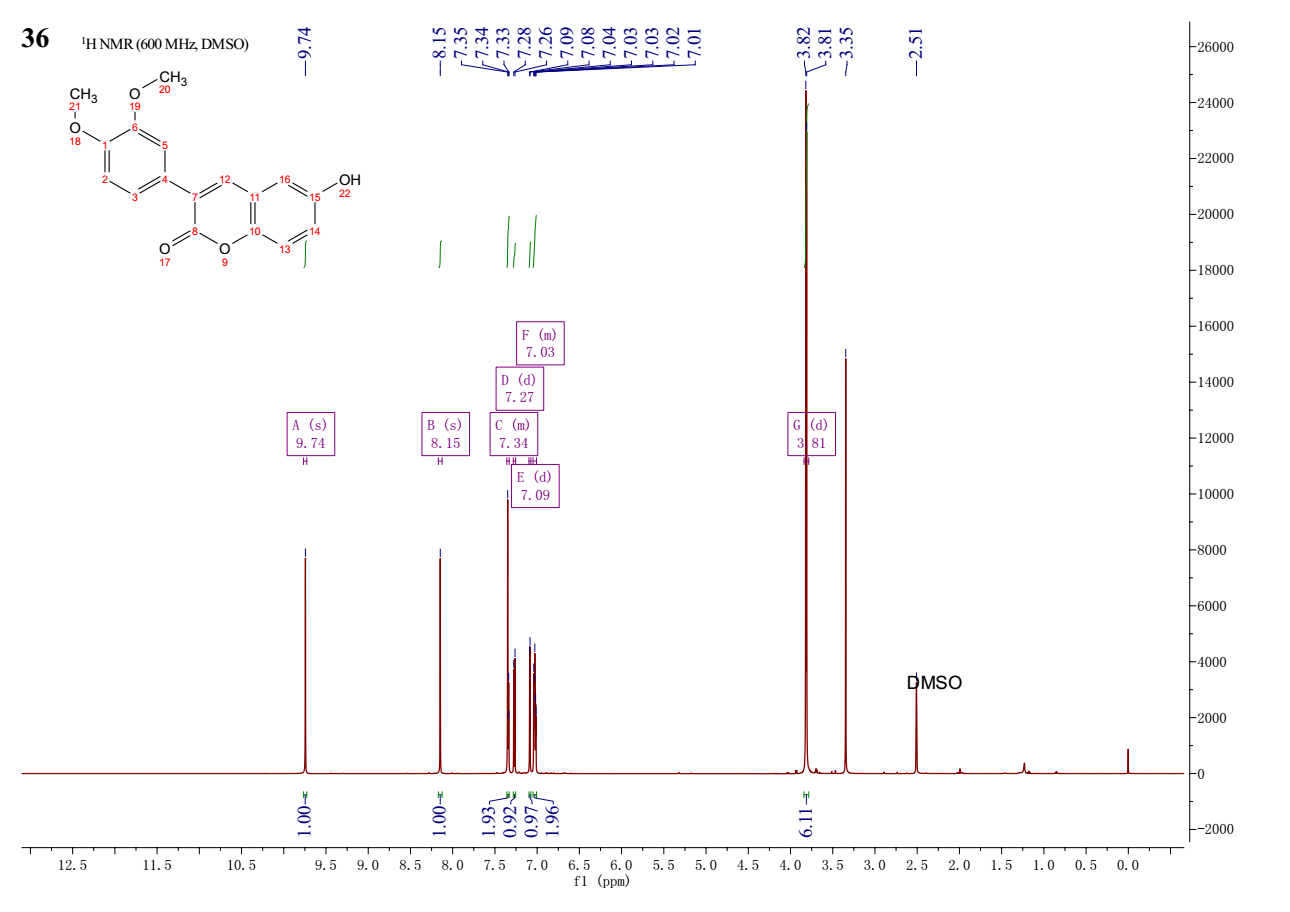


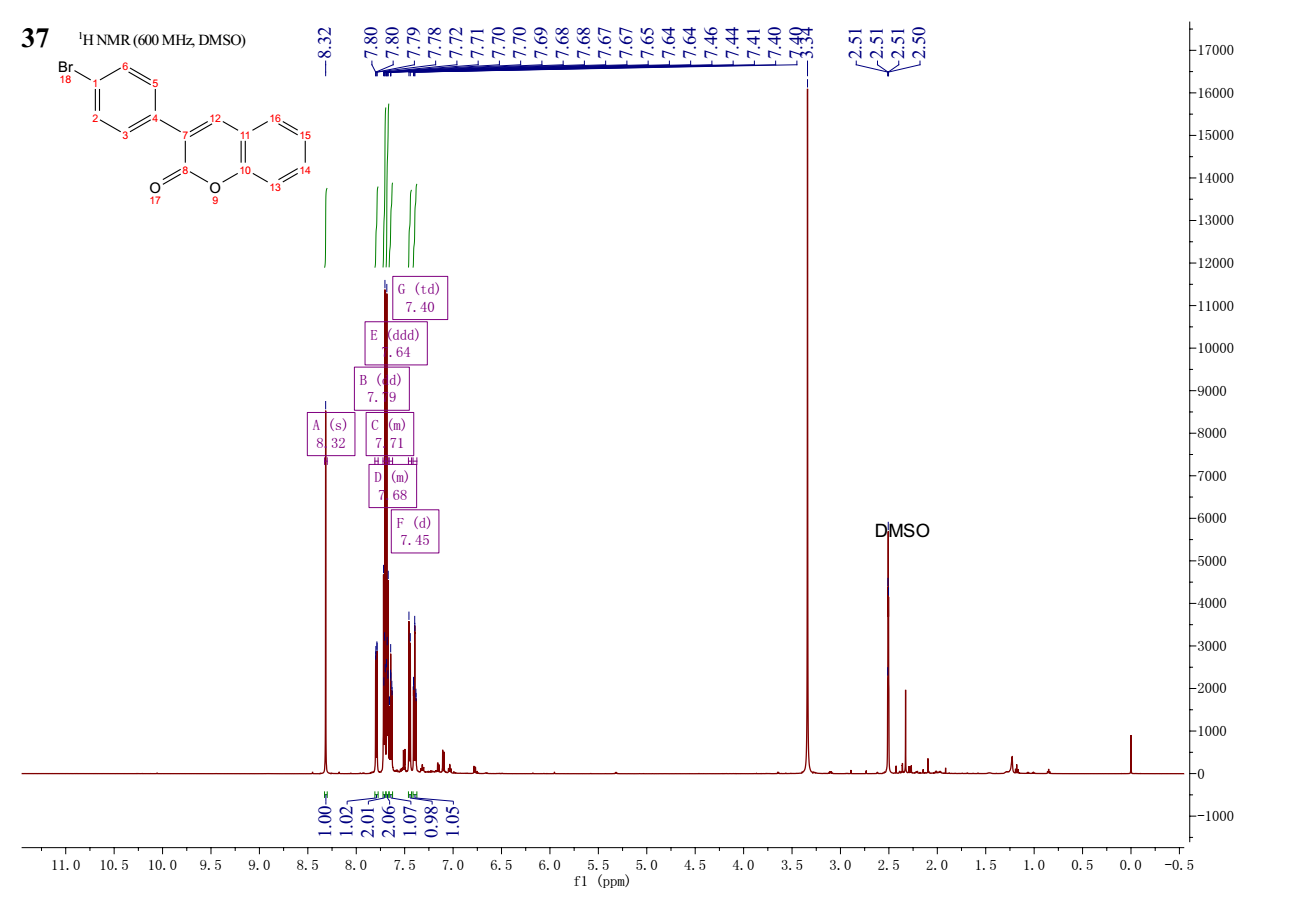


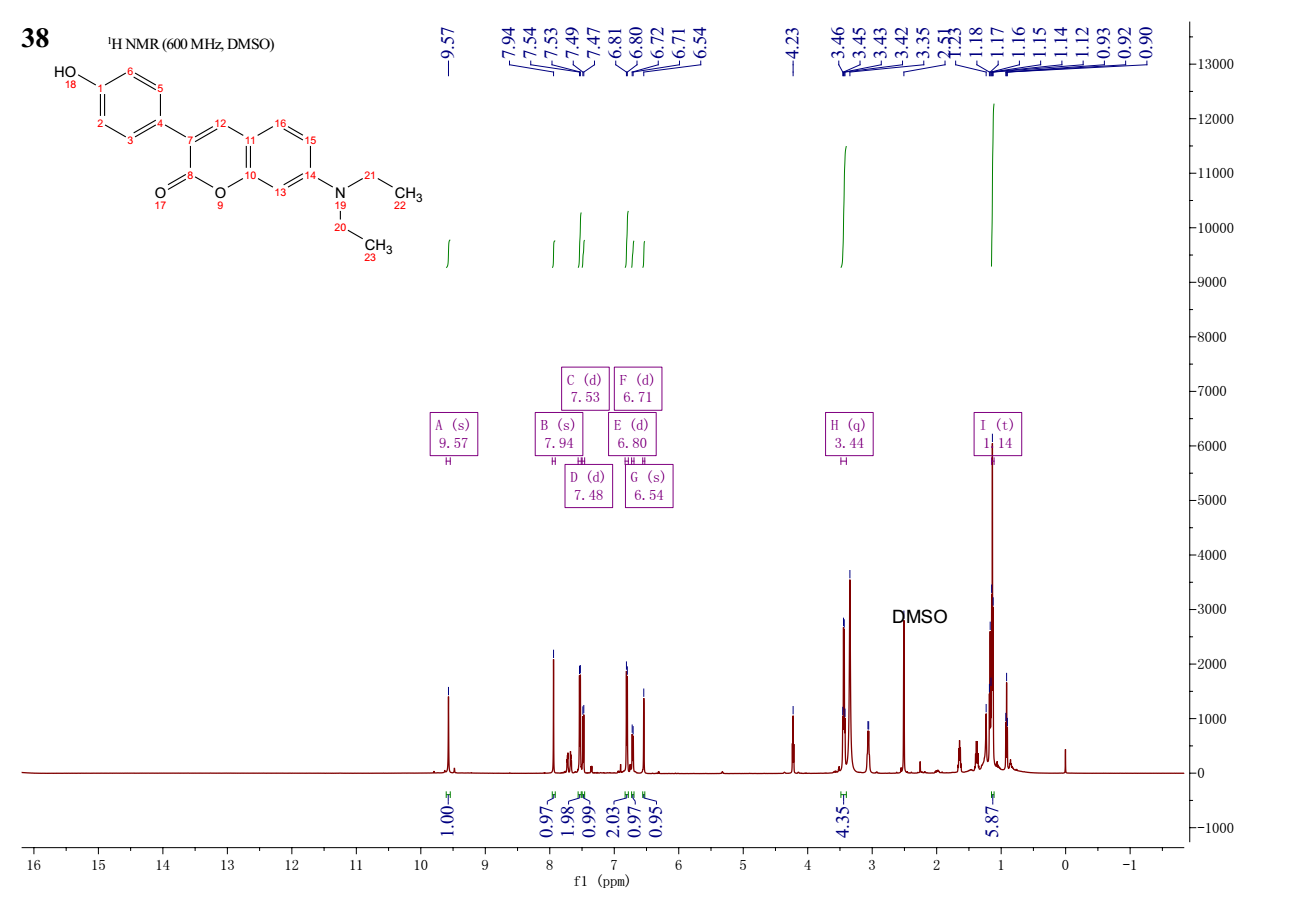


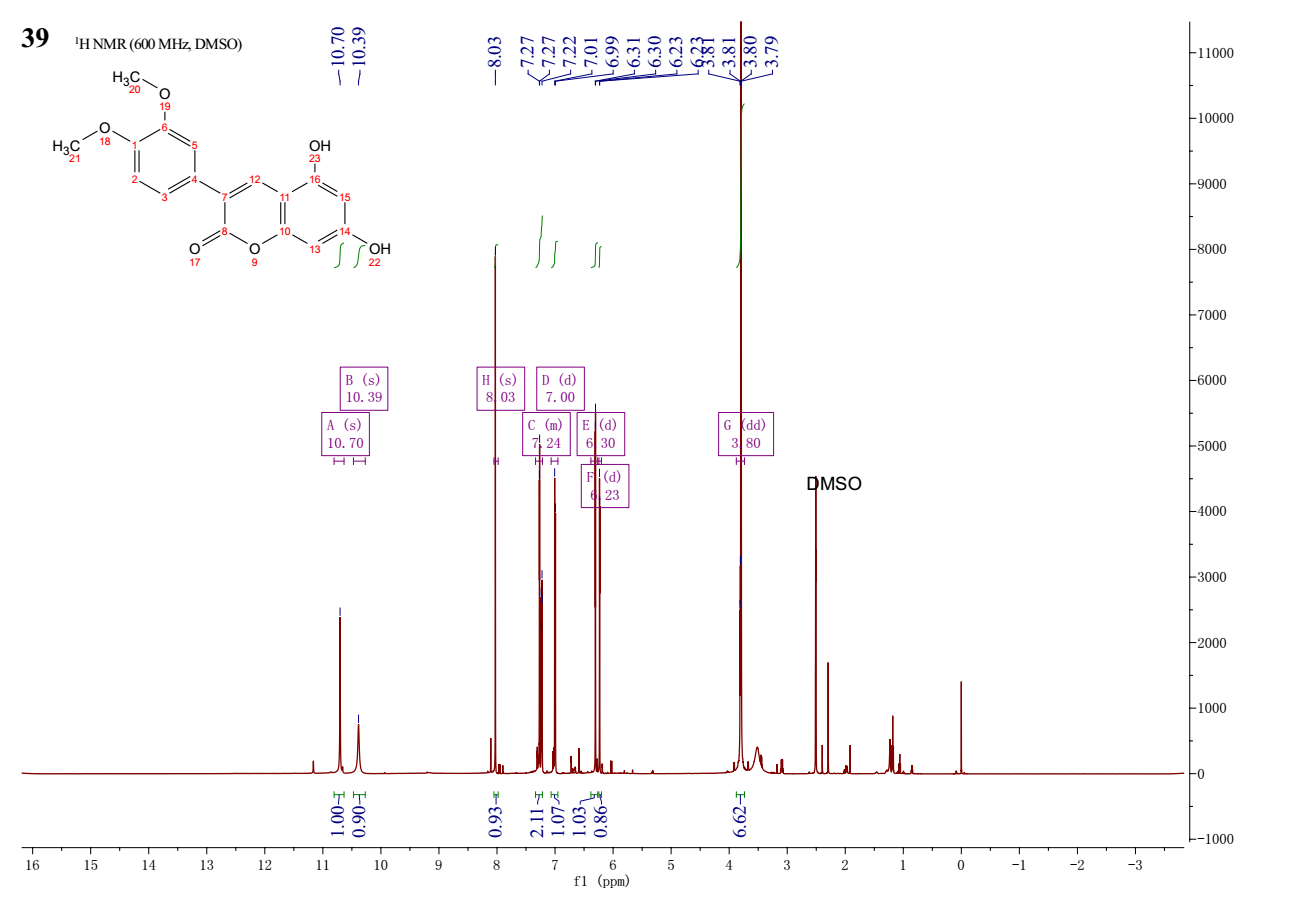


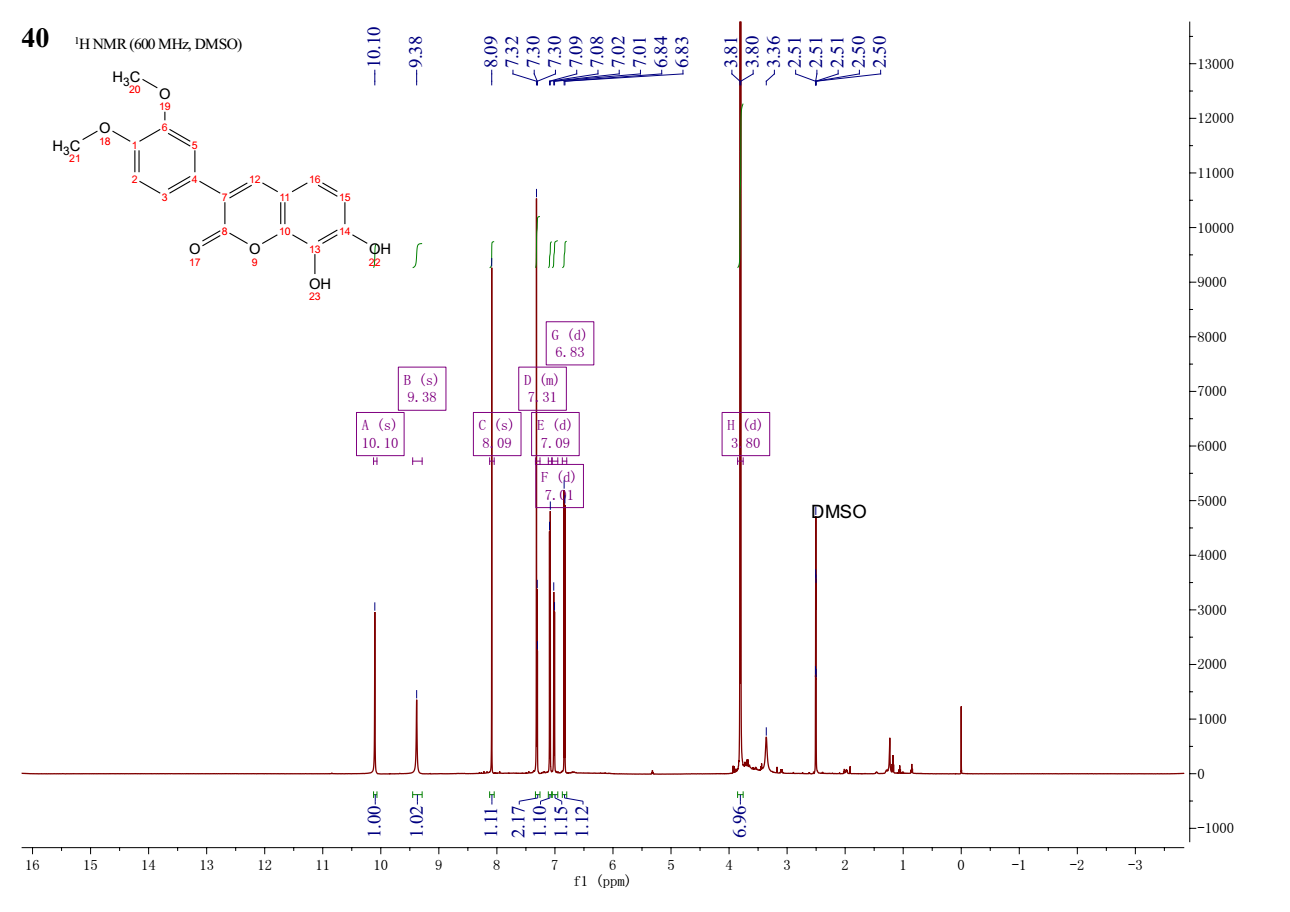


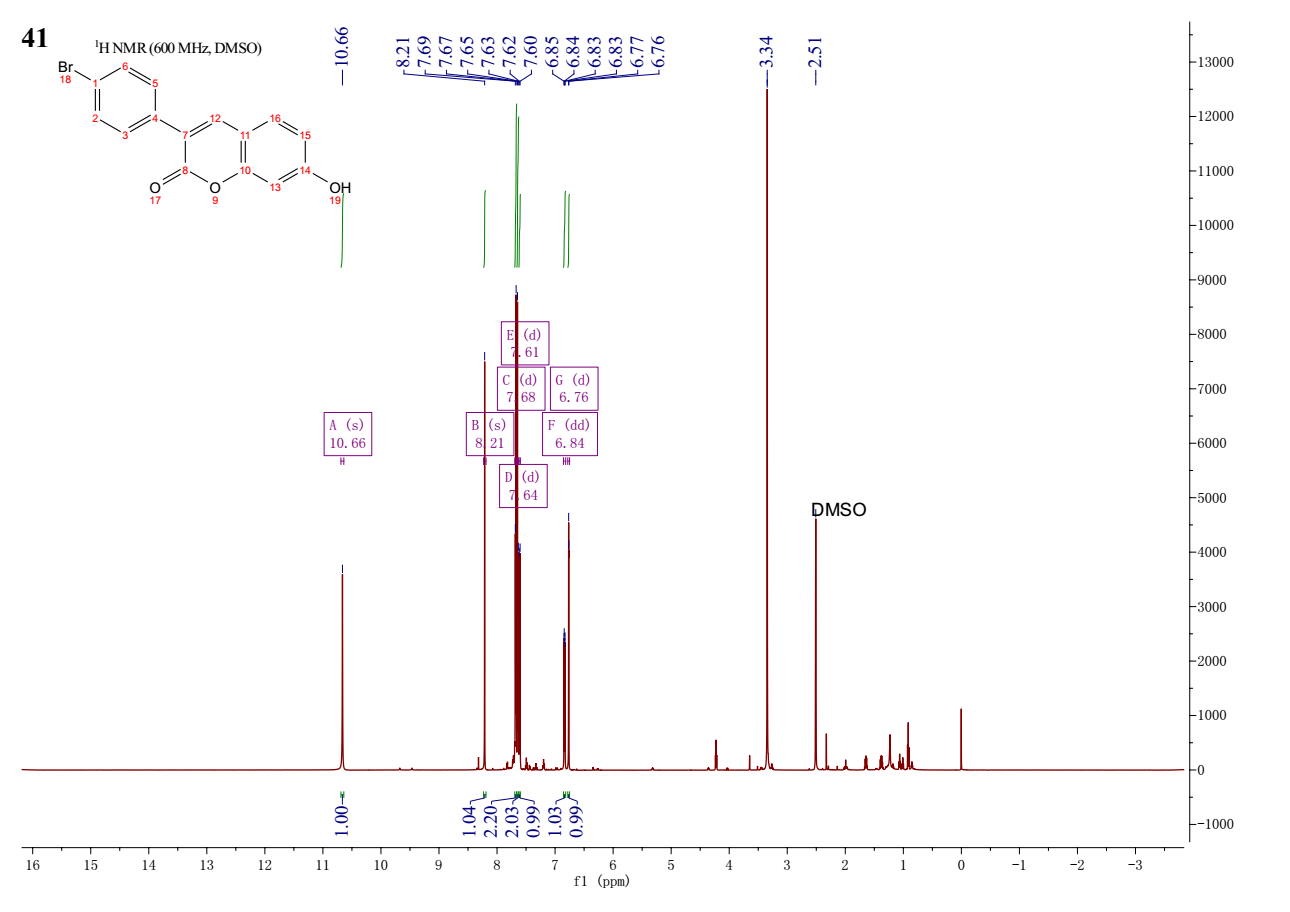


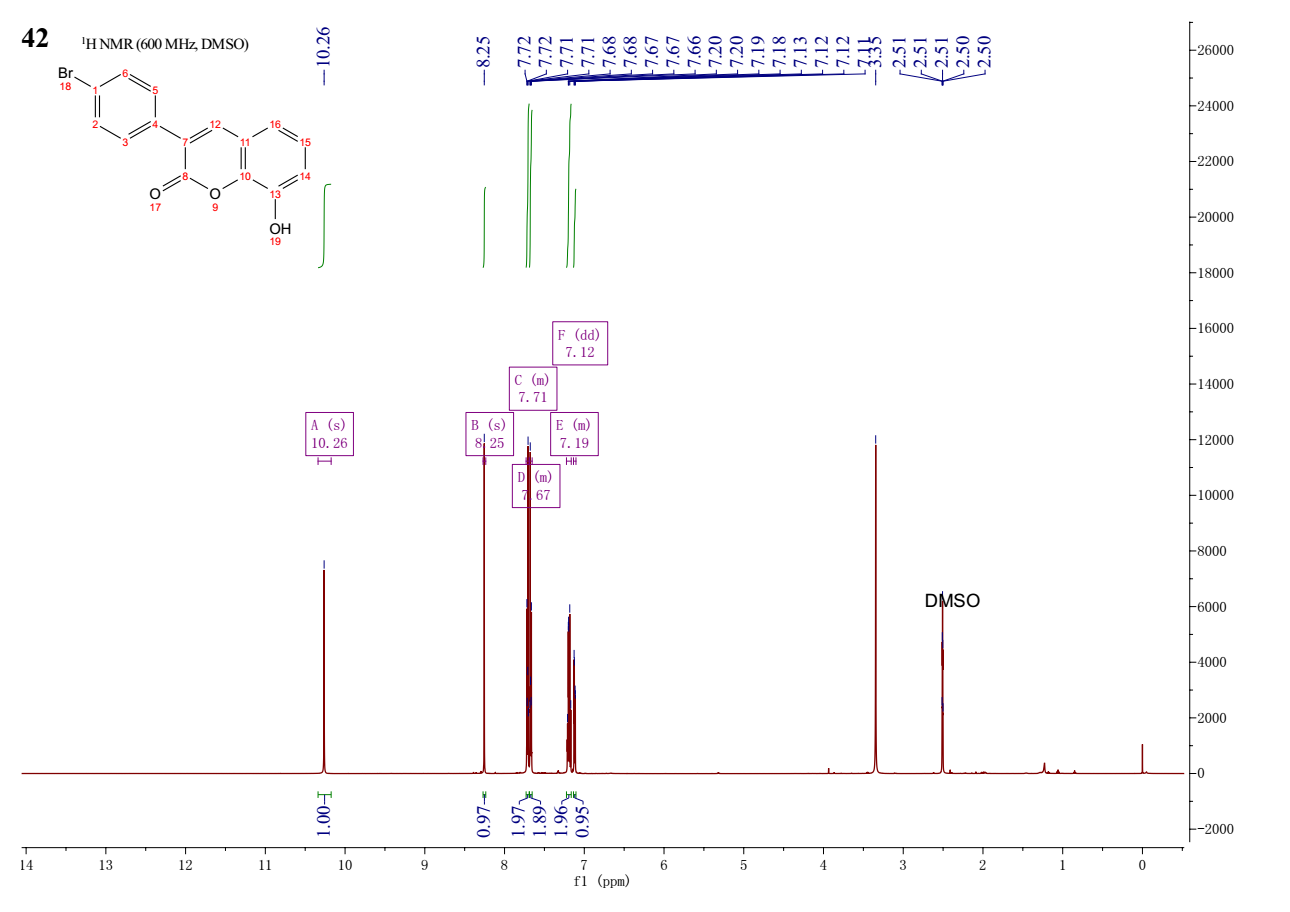


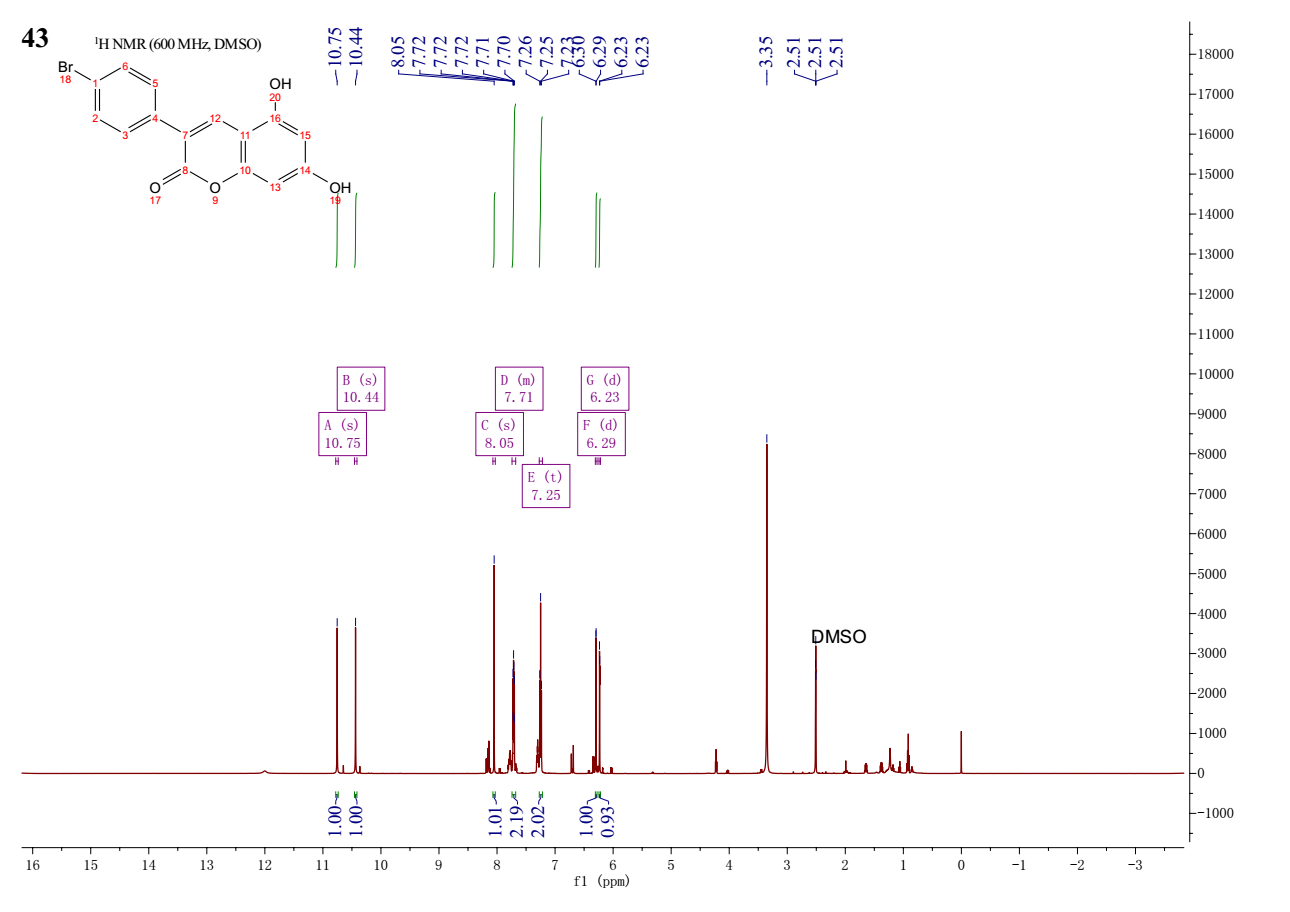


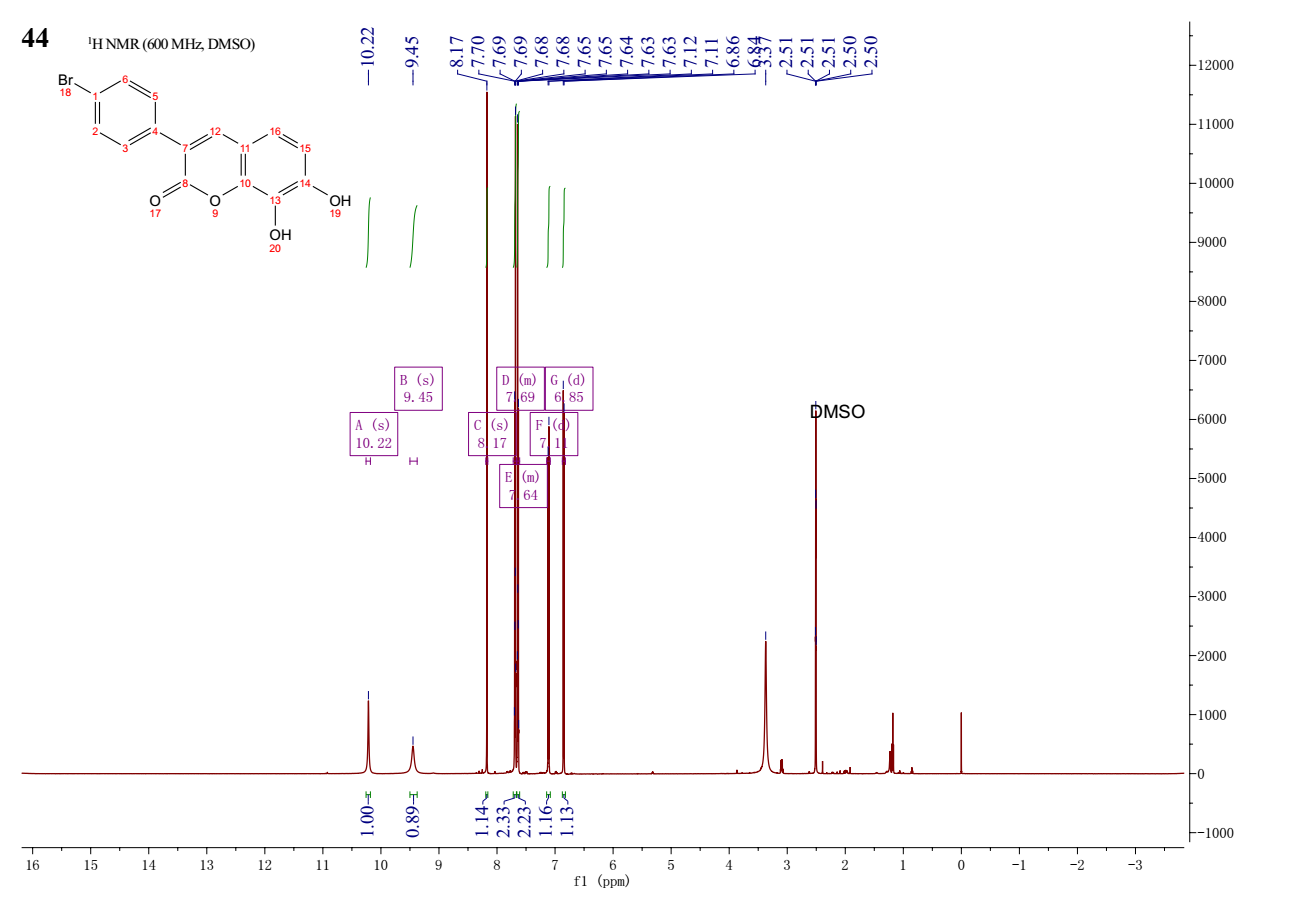


**^13^C NMR(600 MHz, DMSO-d_6_) of compounds 1-44**


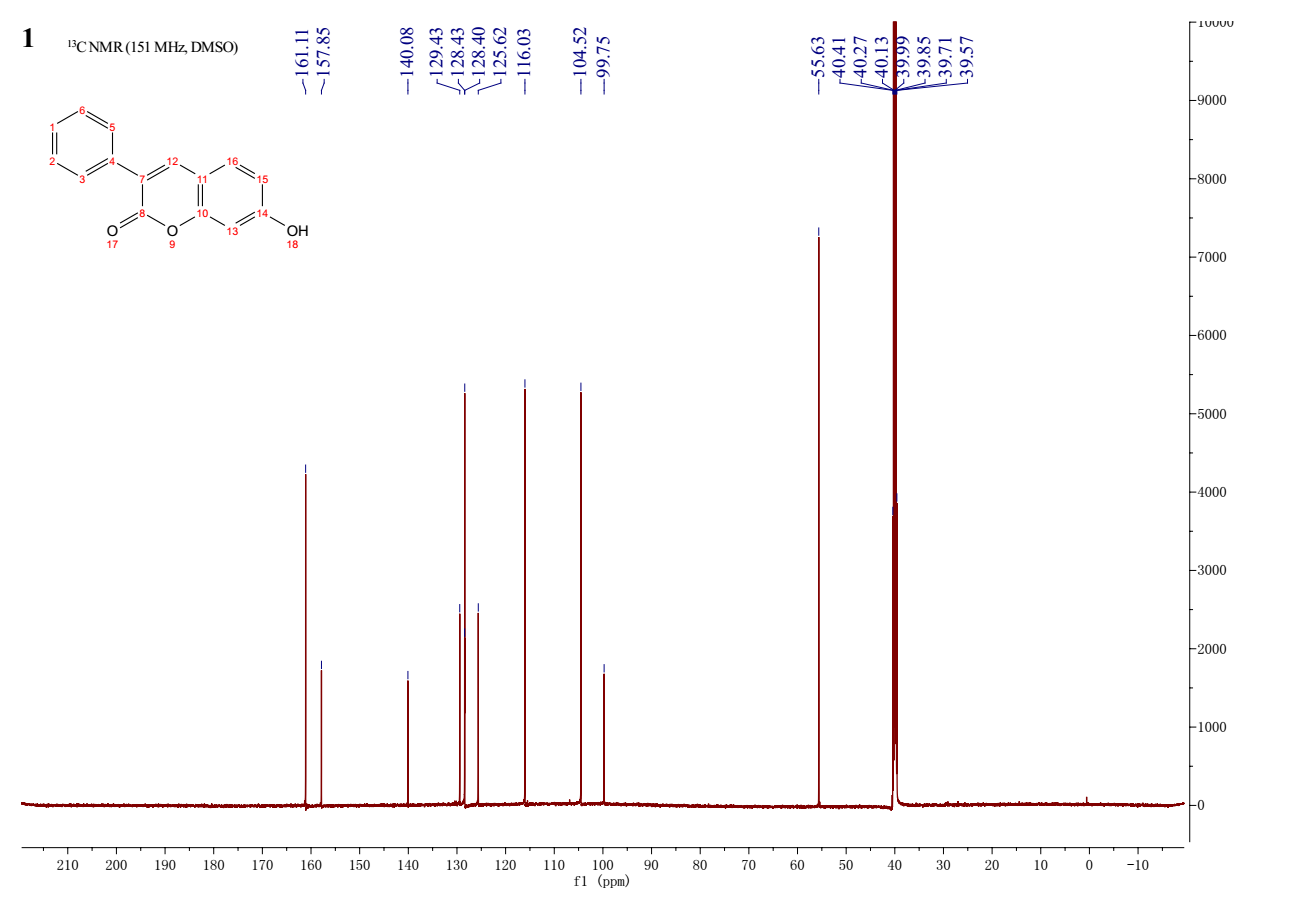


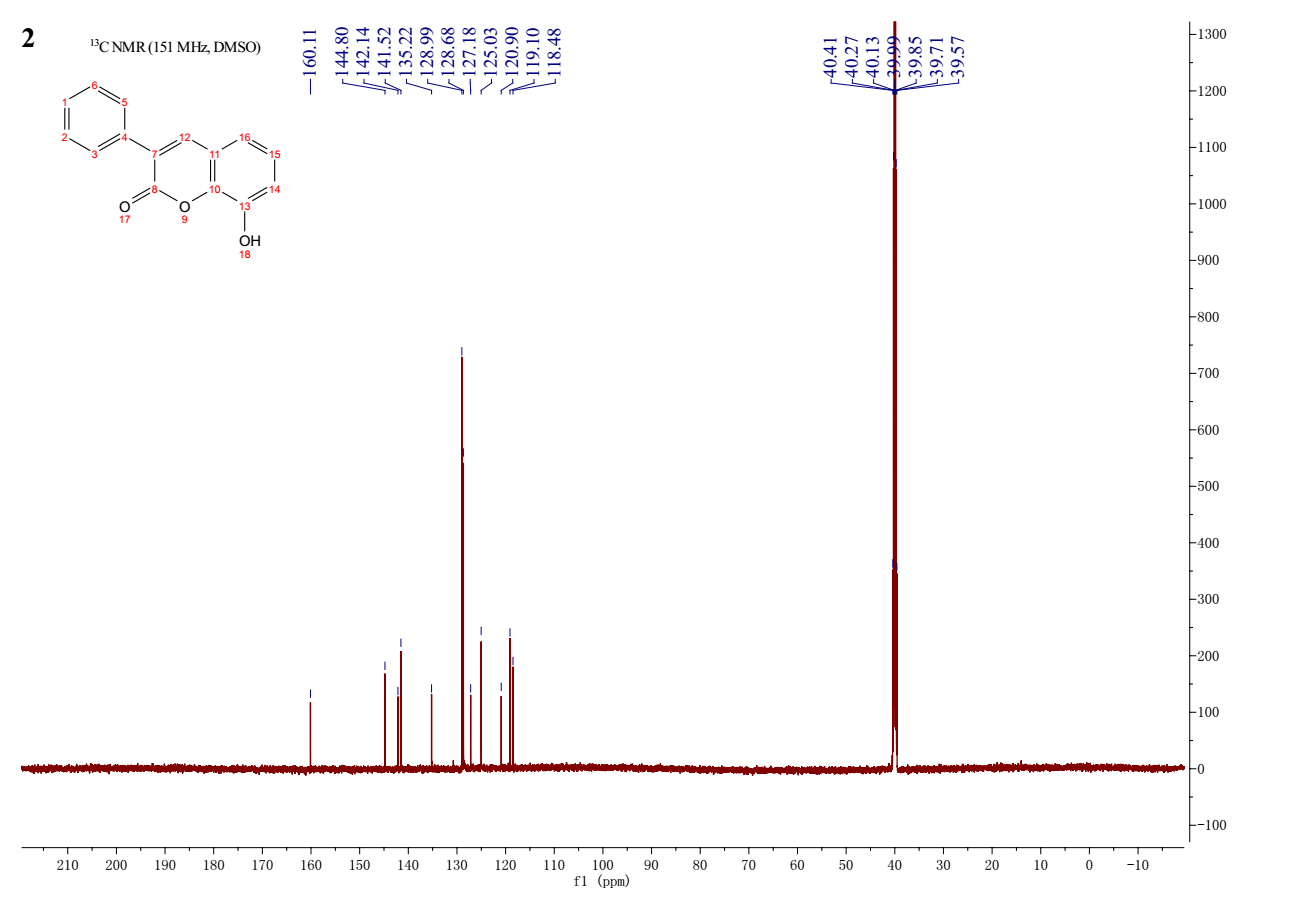


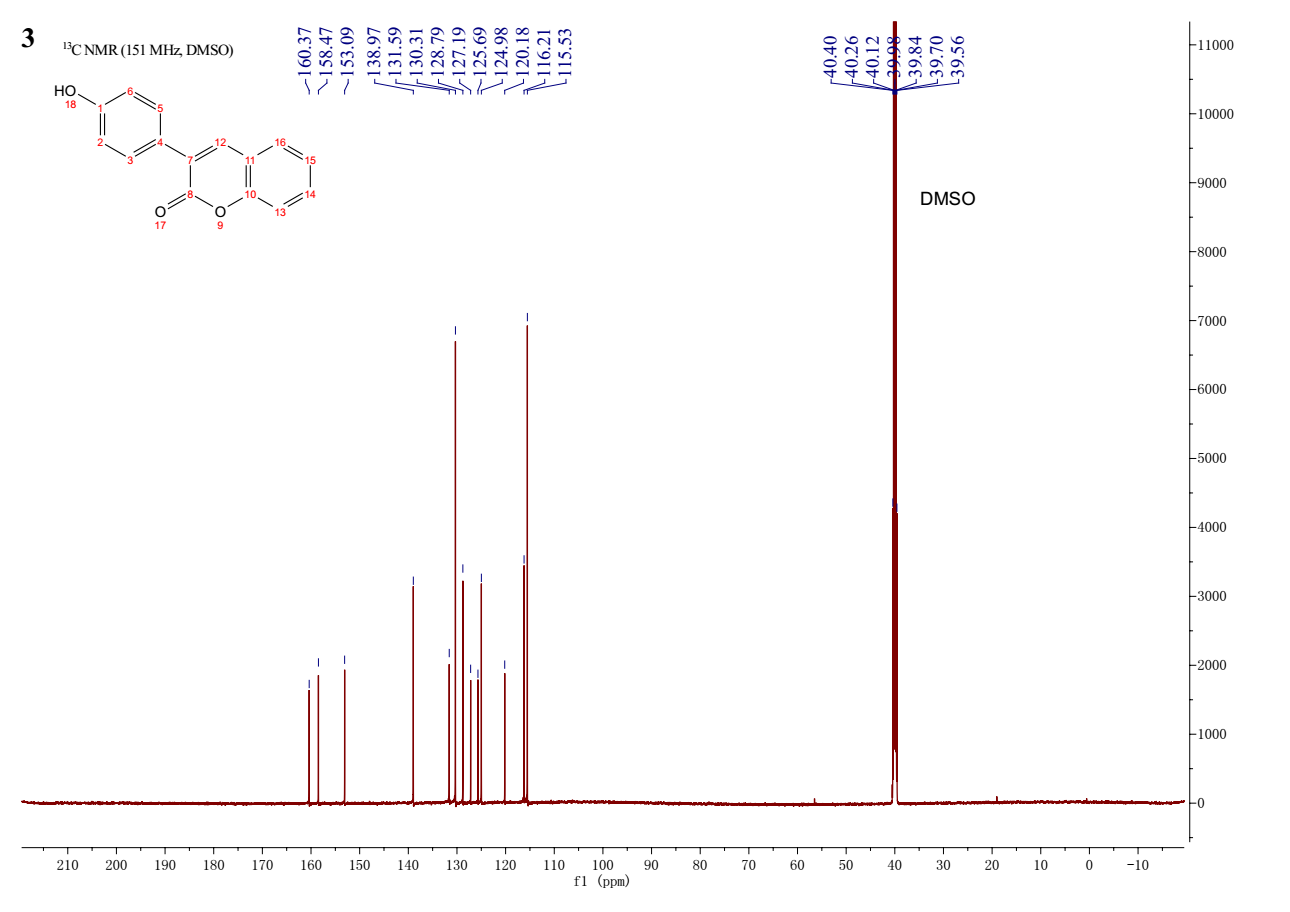


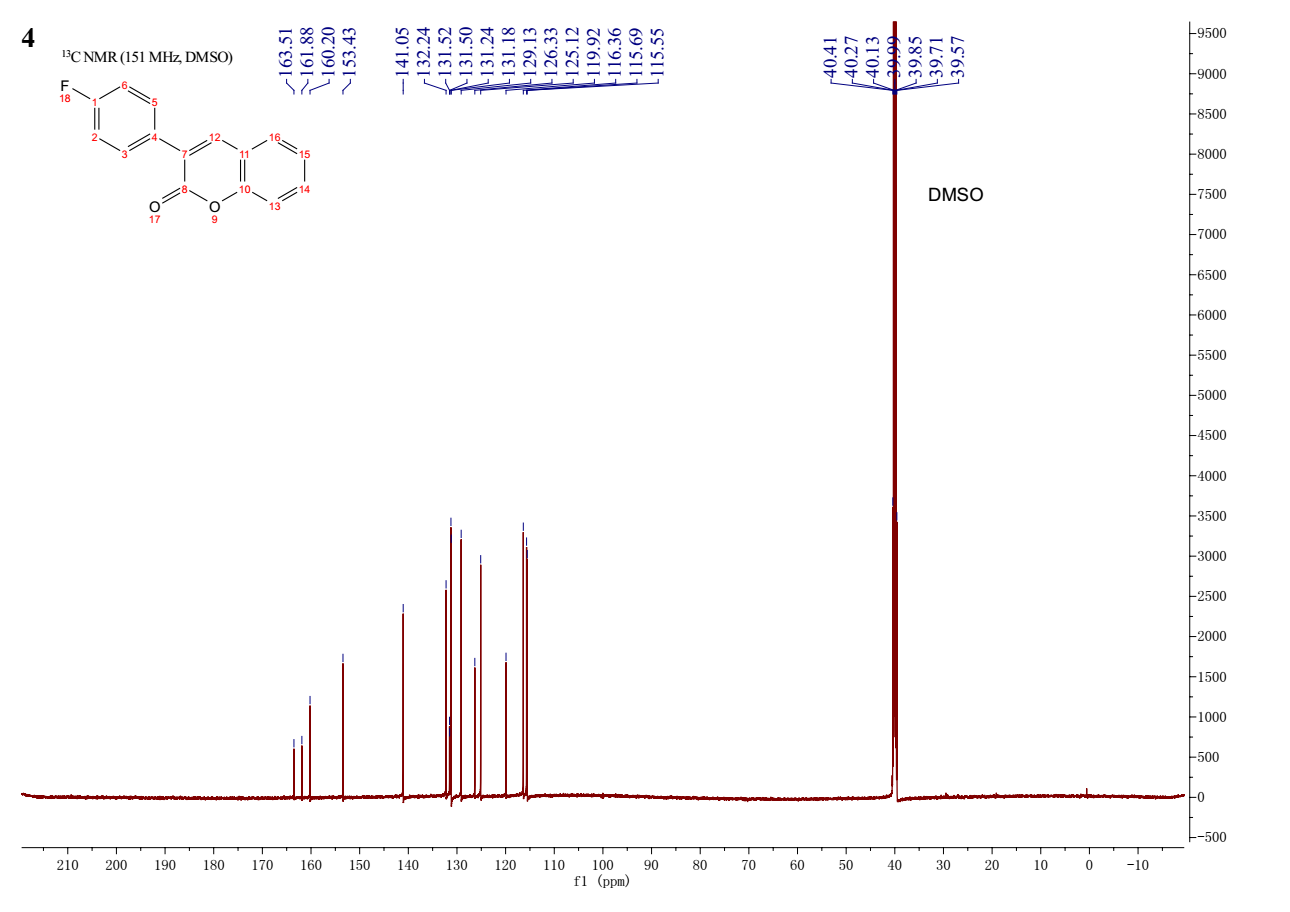


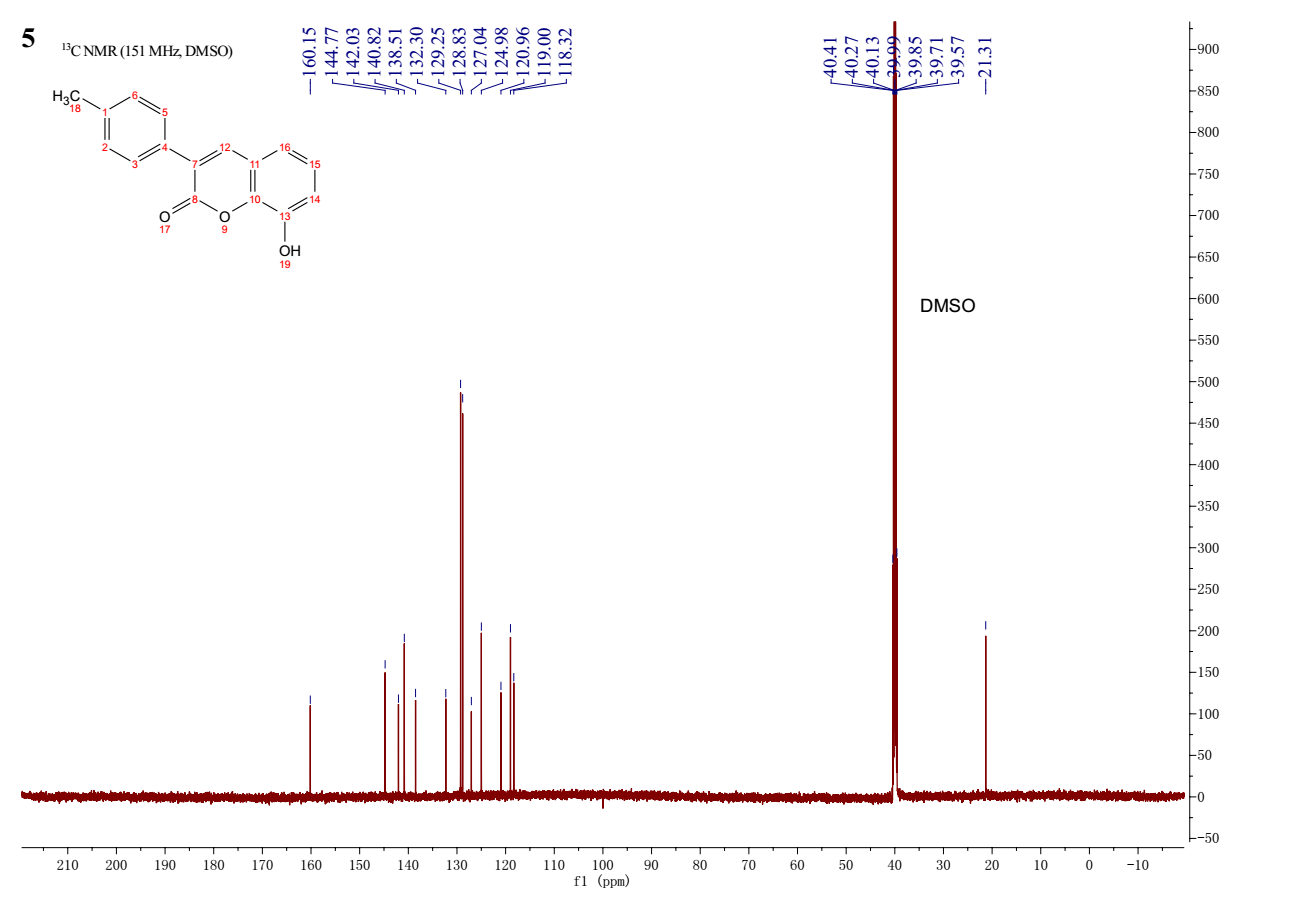


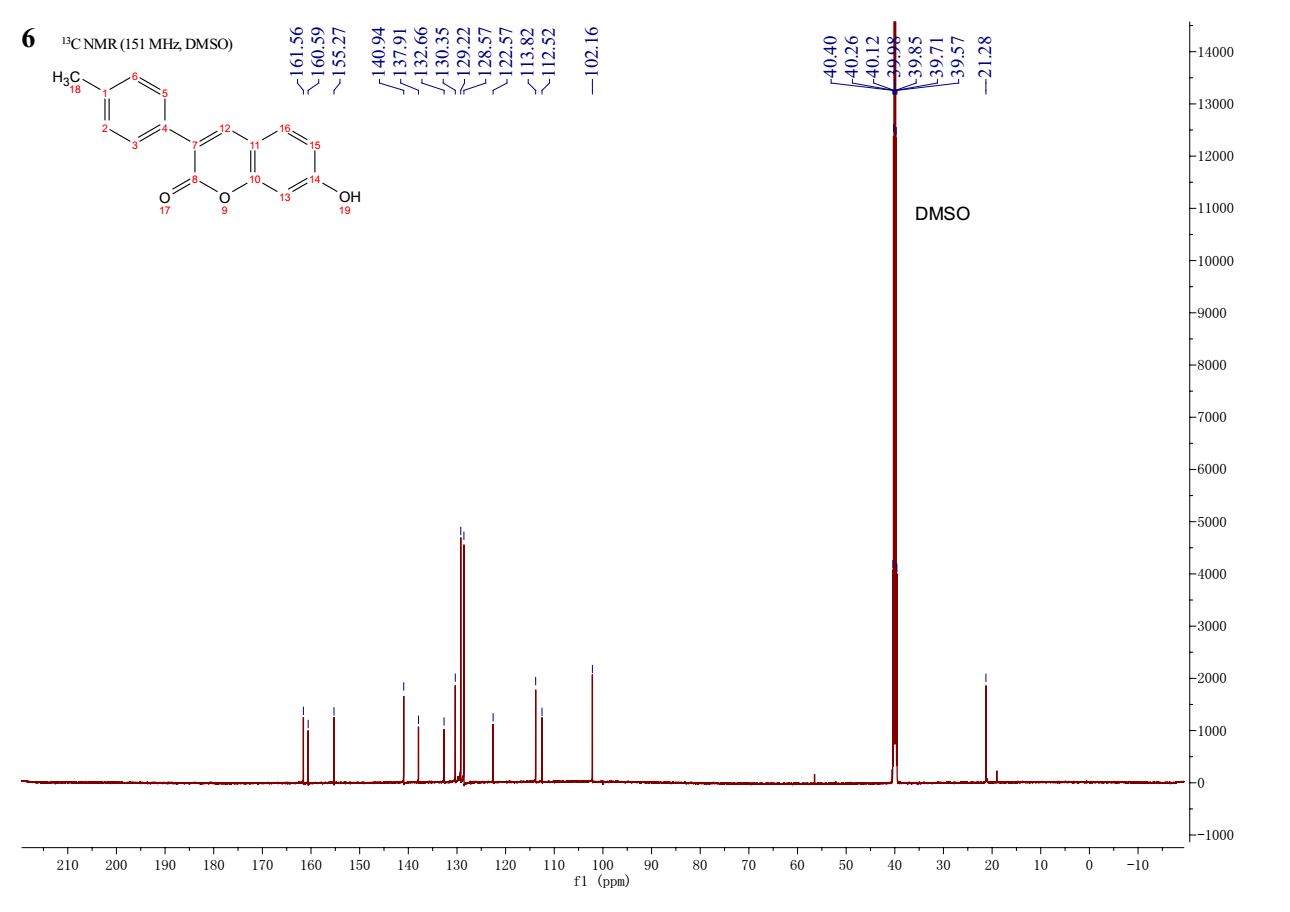


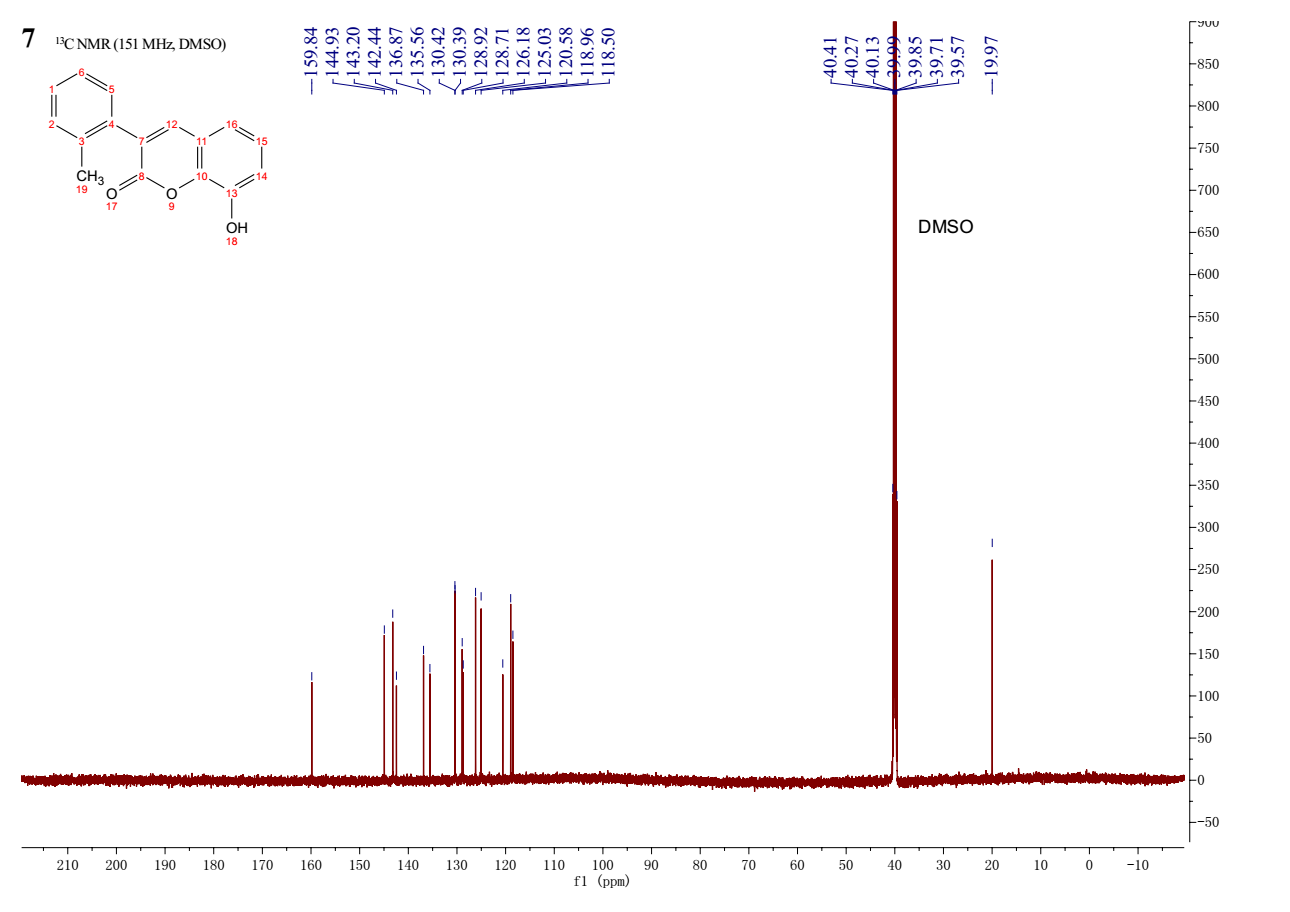


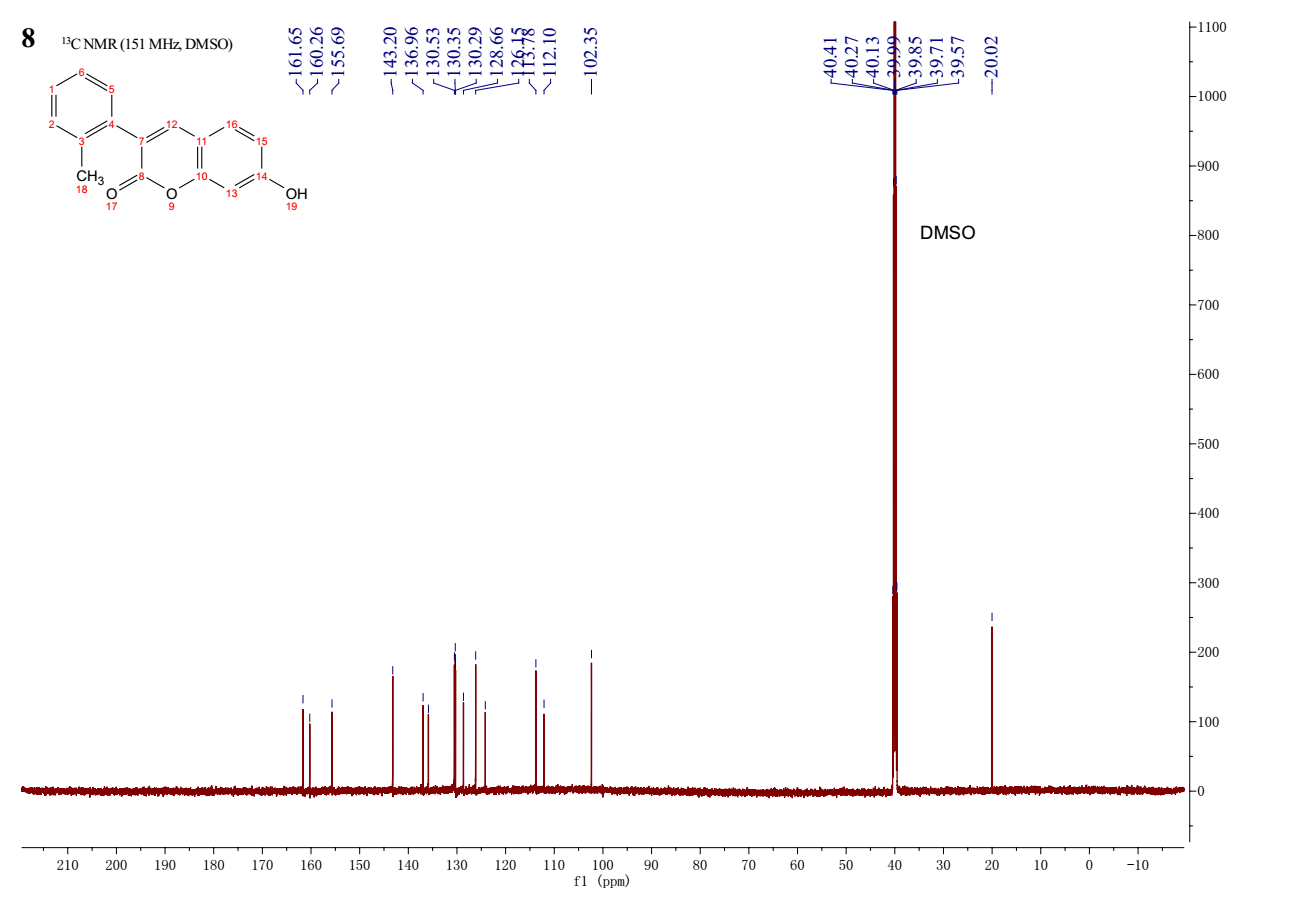


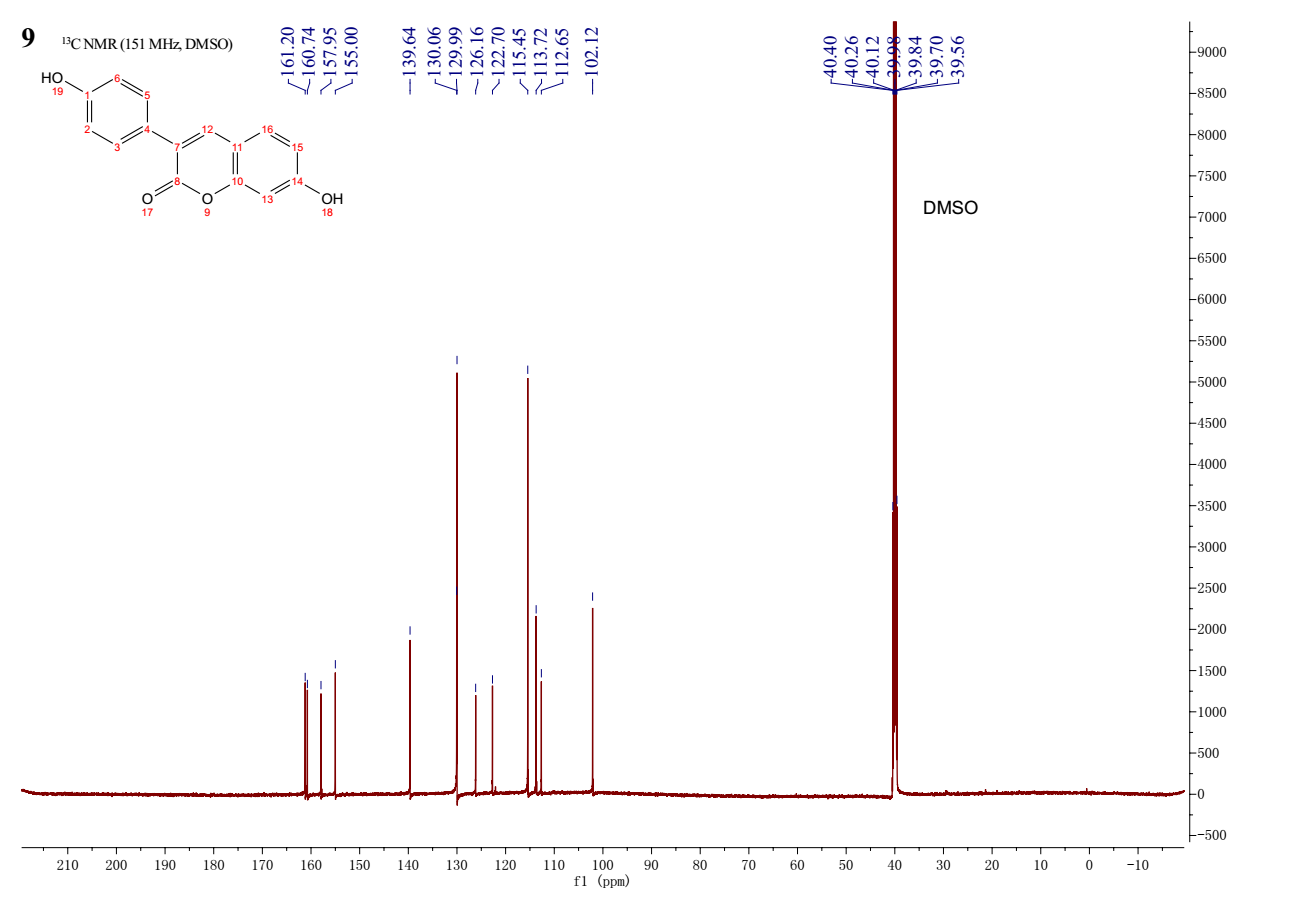


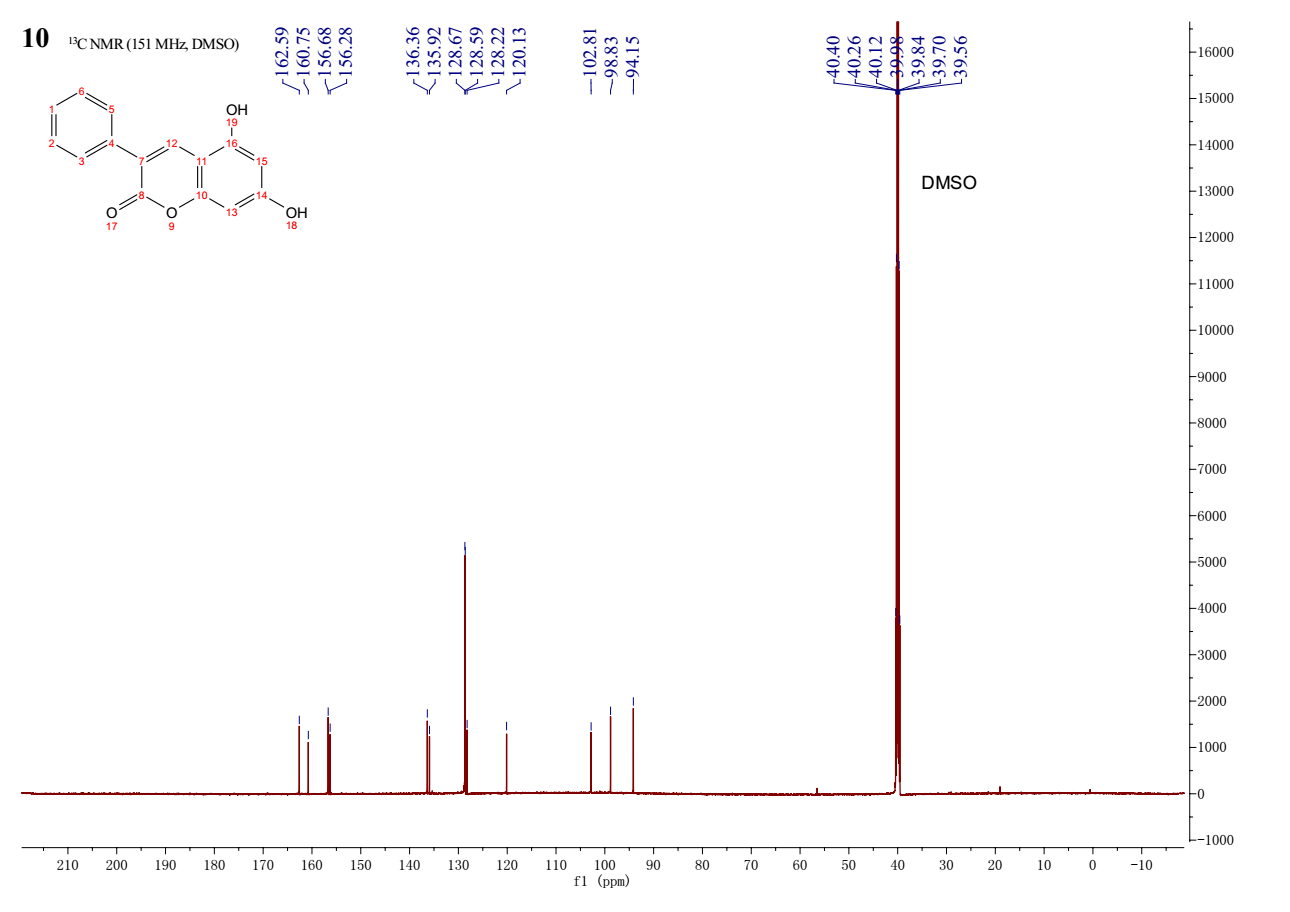


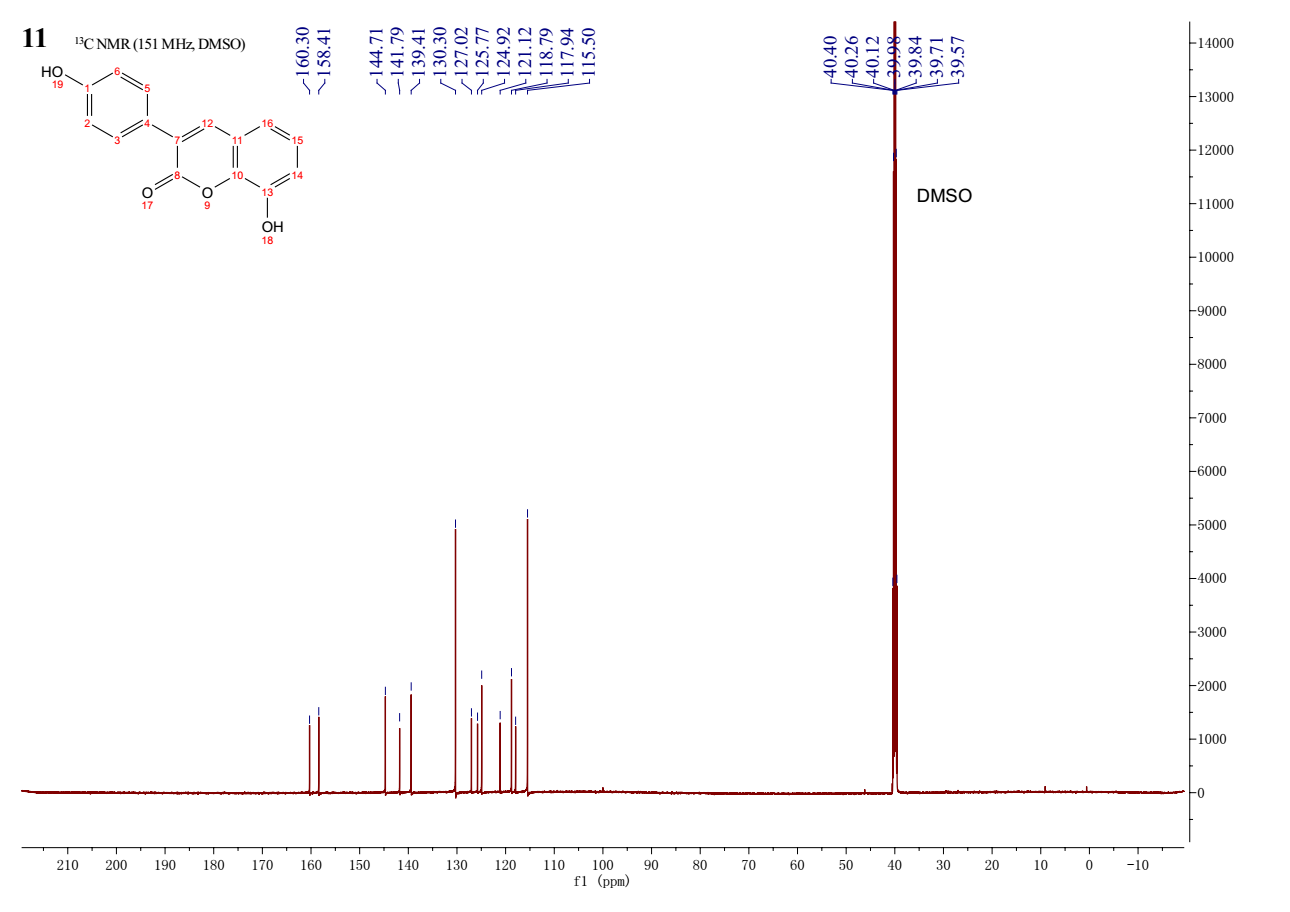


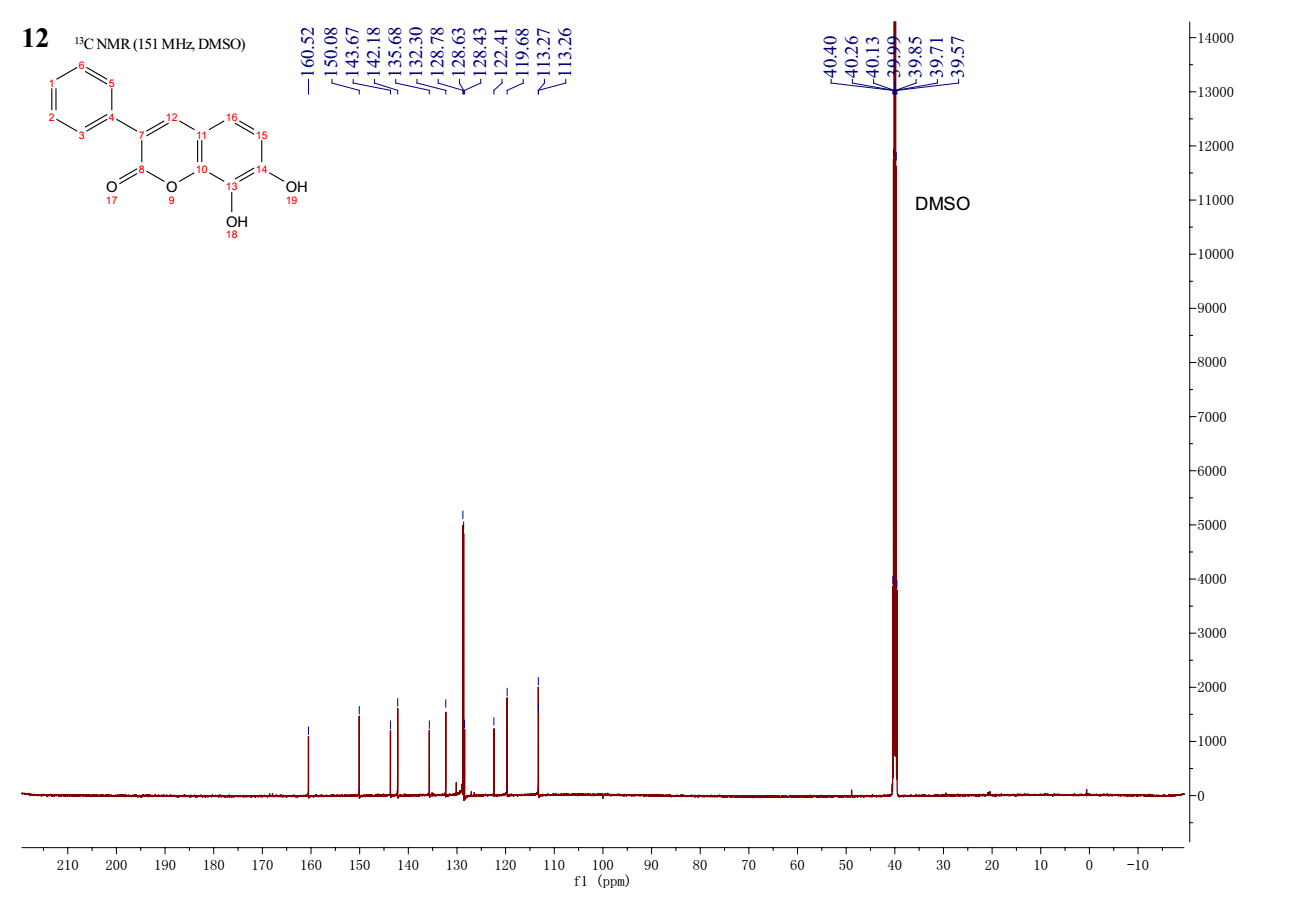


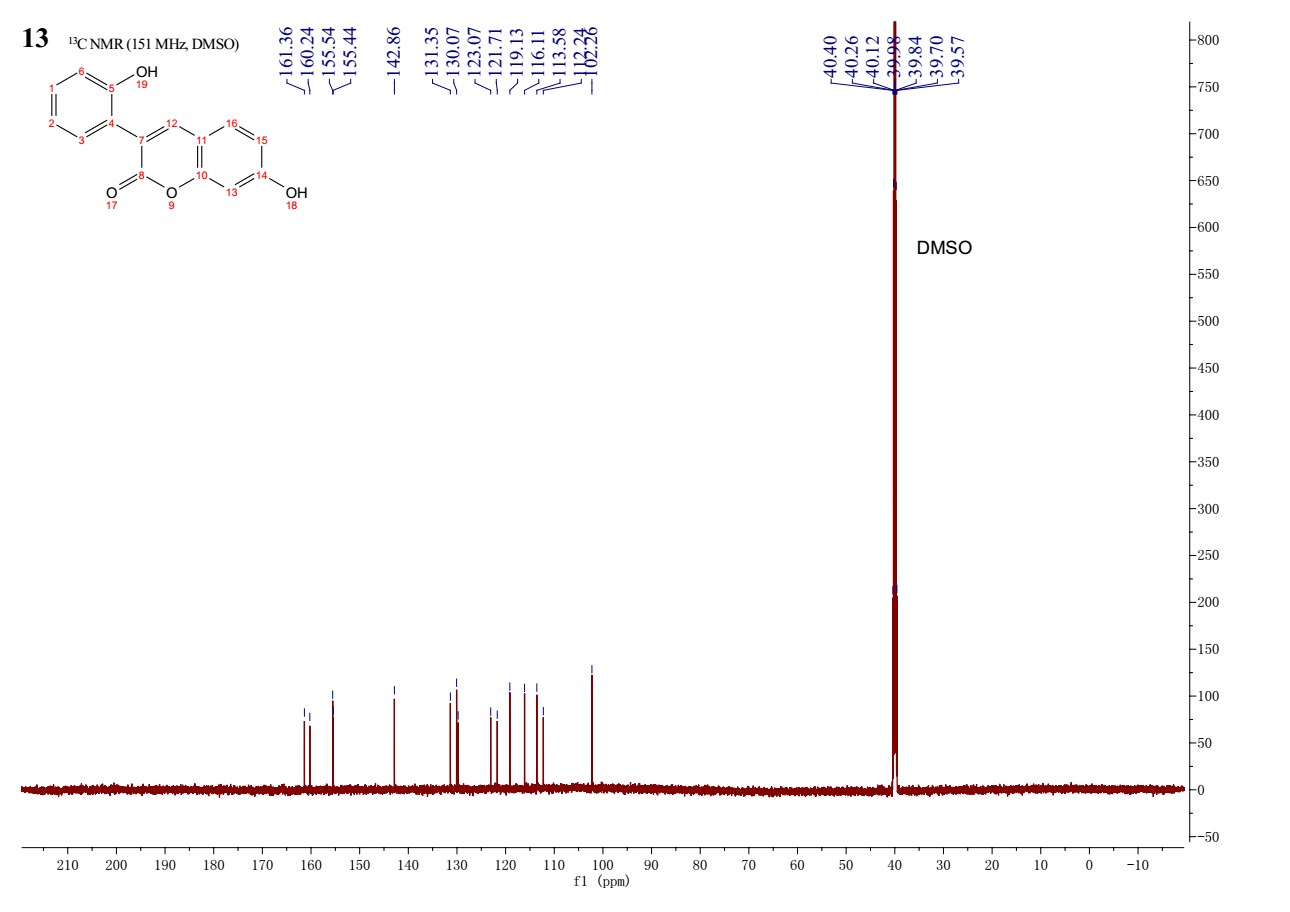


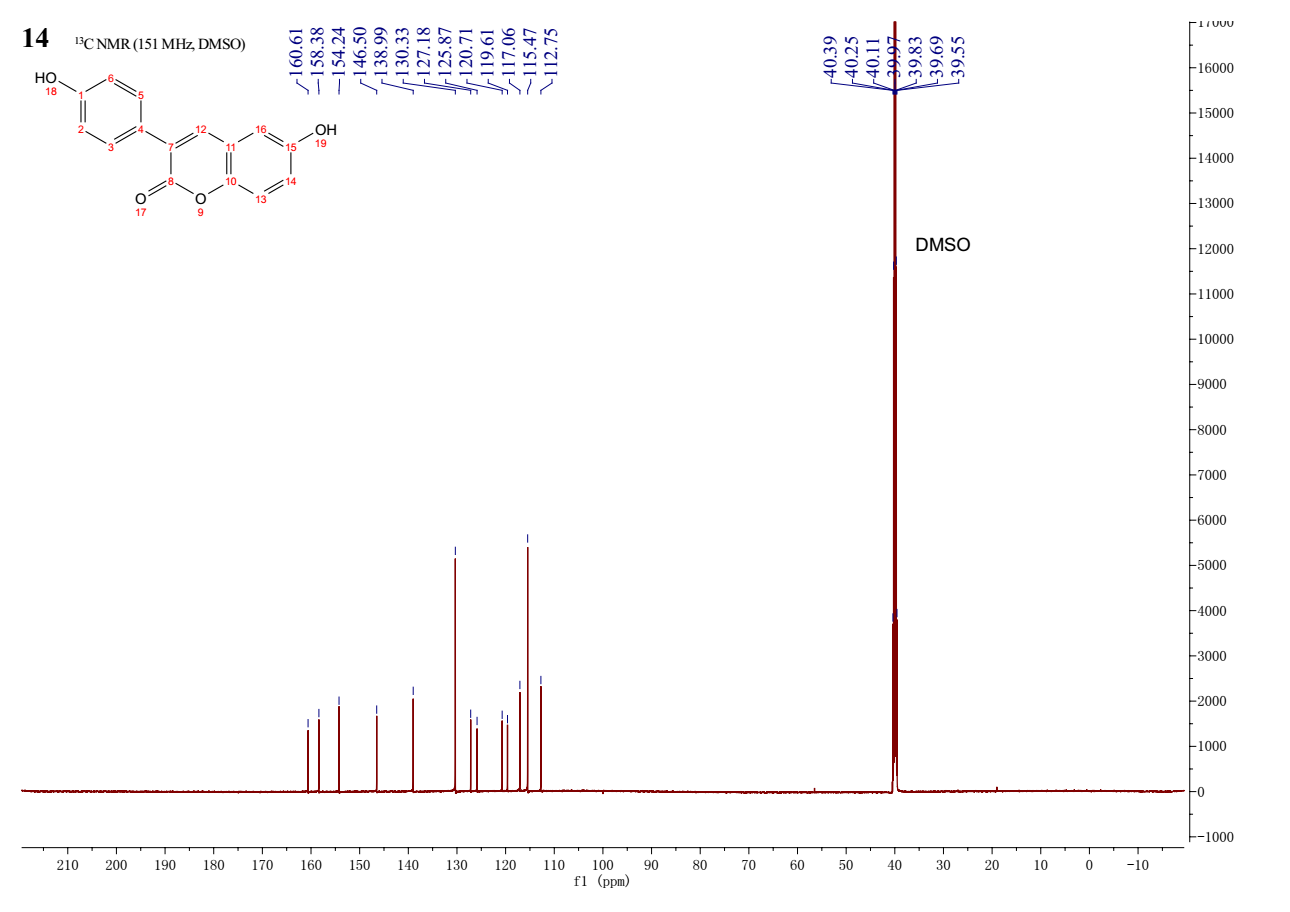


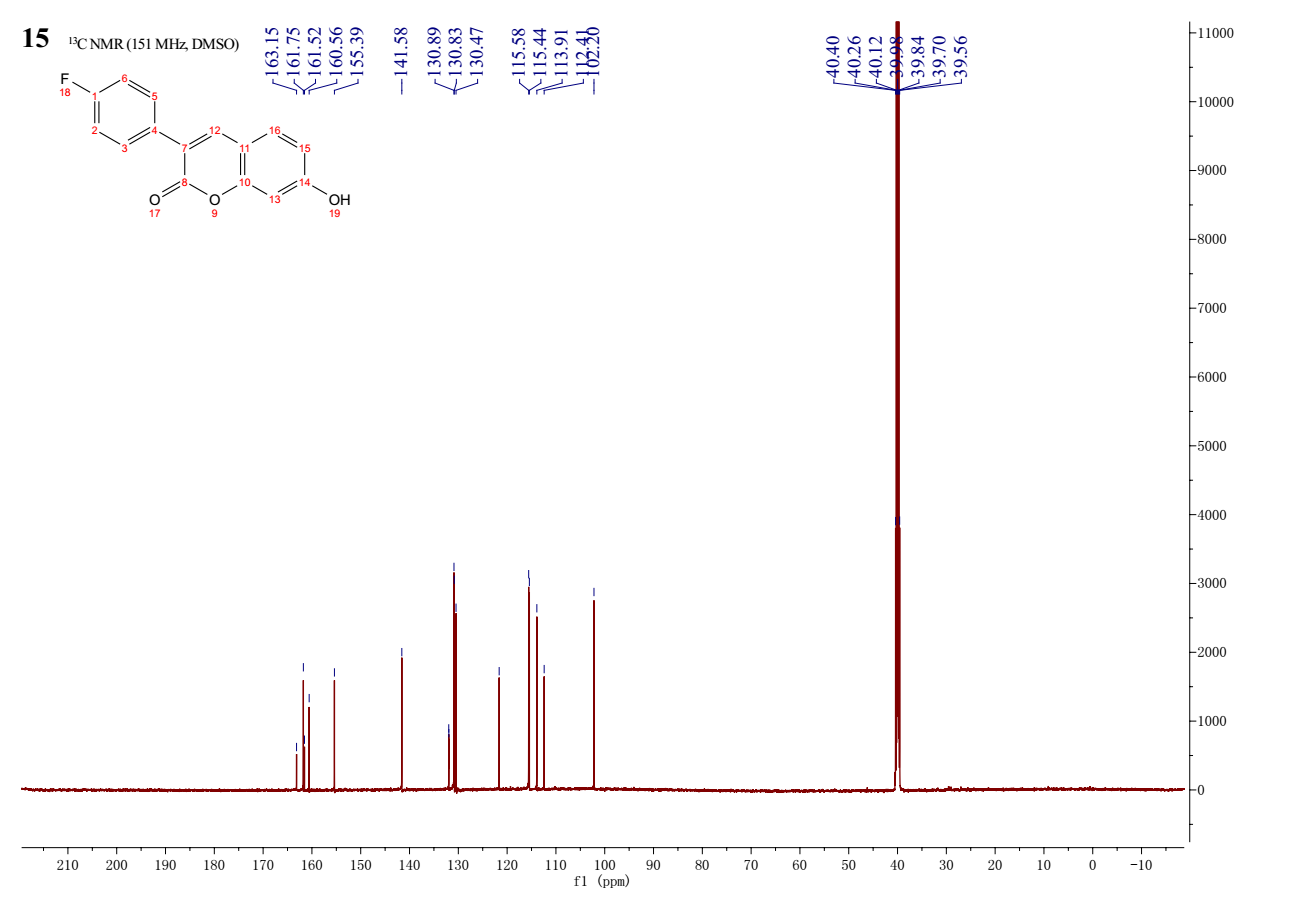


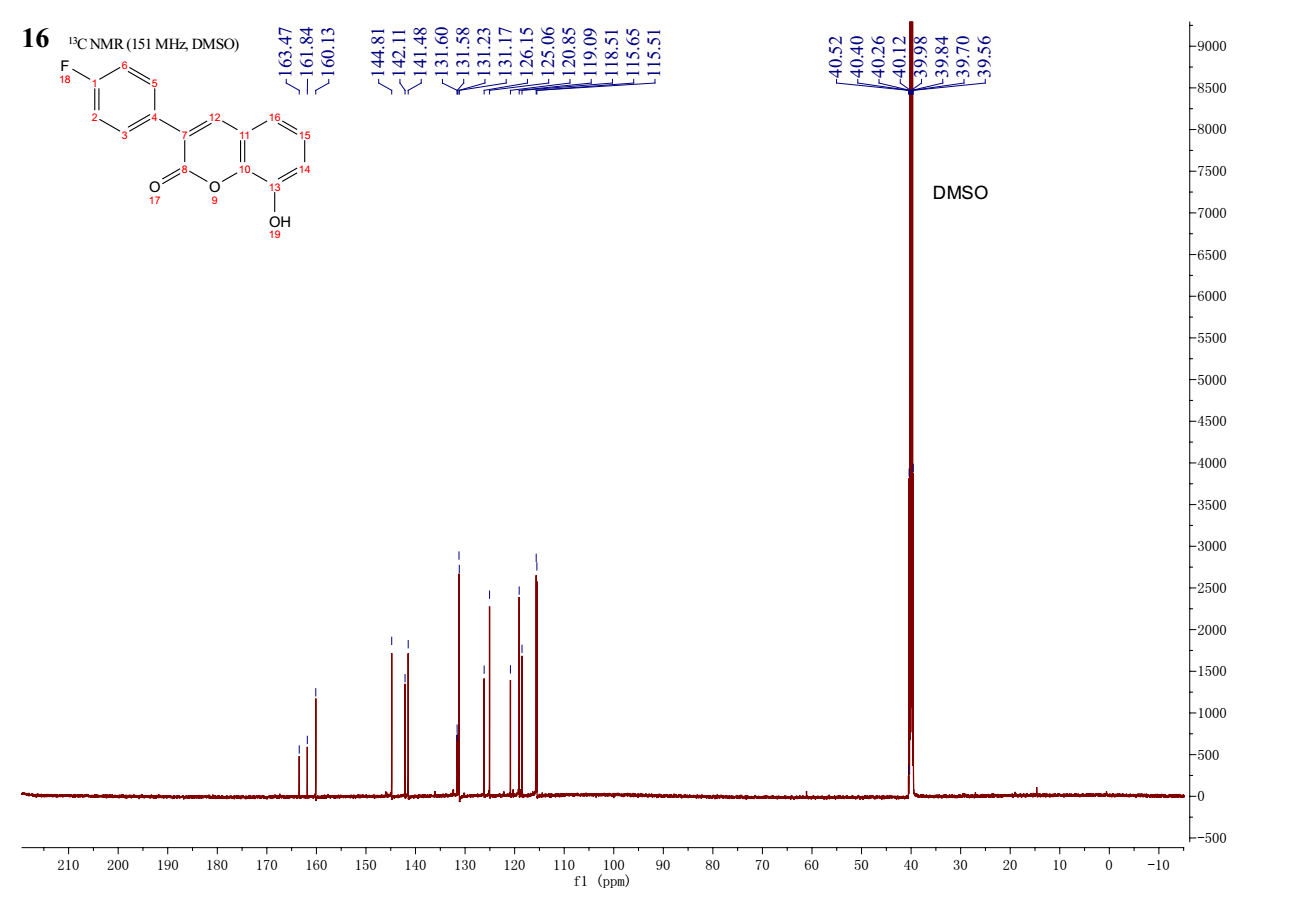


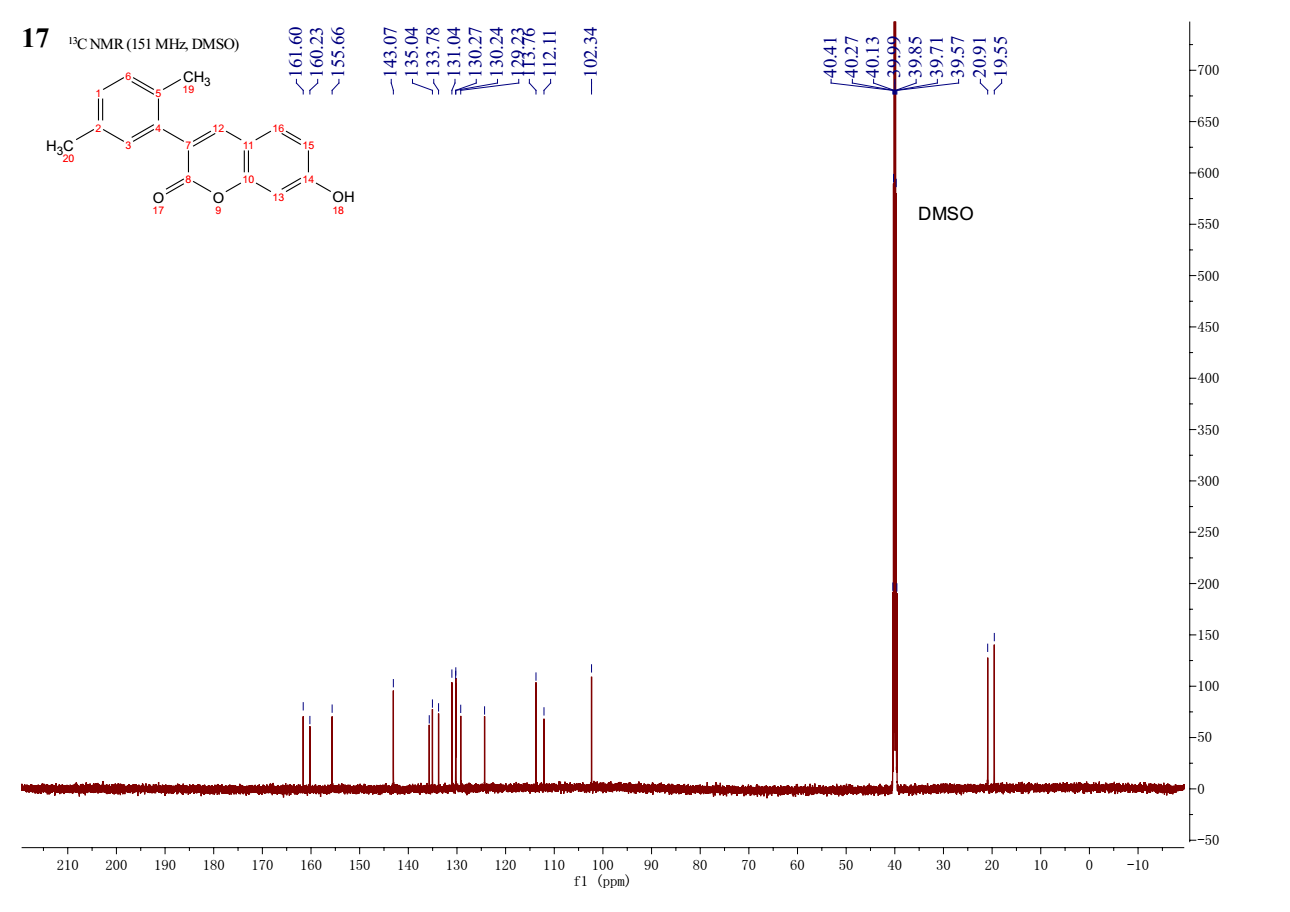


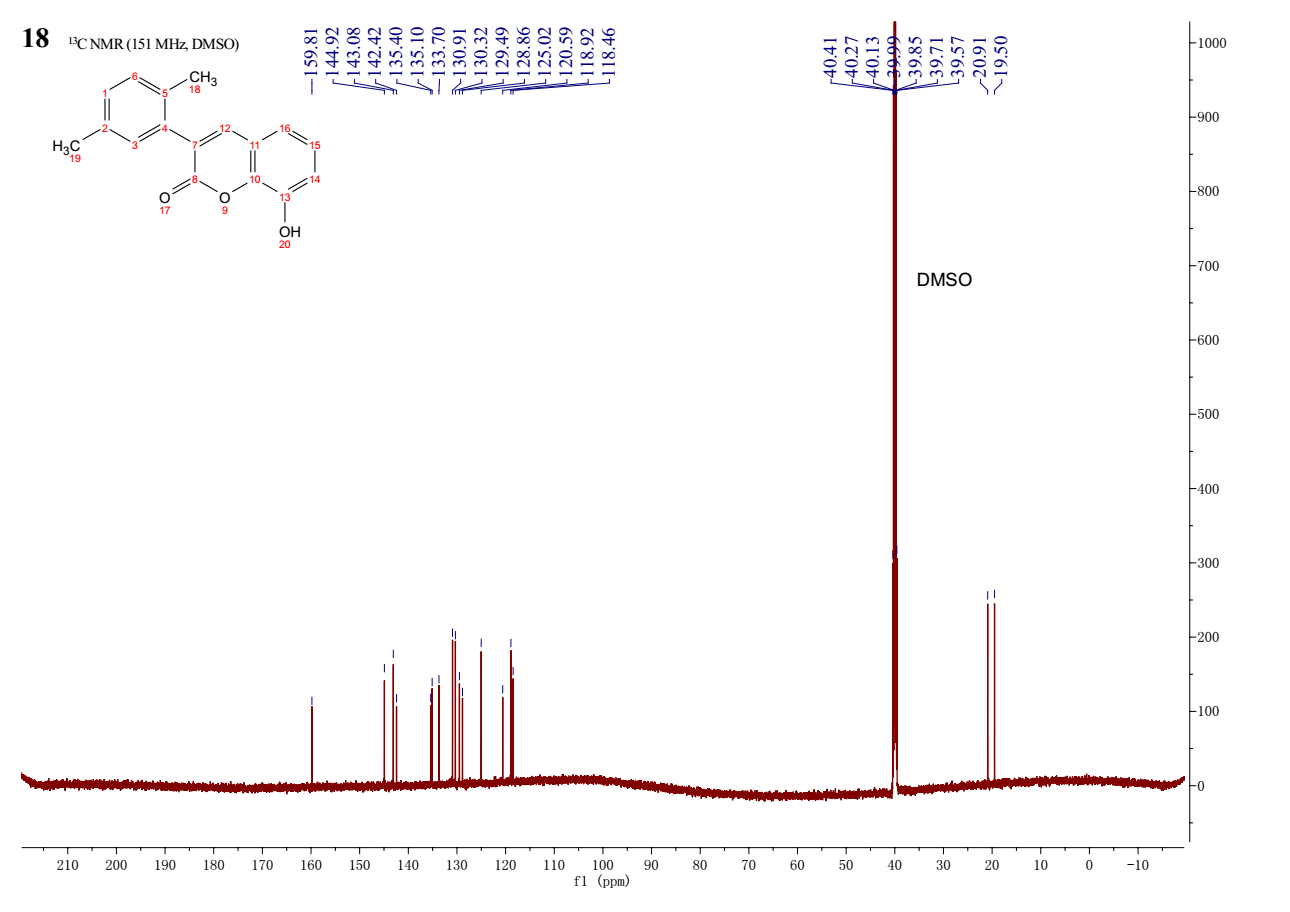


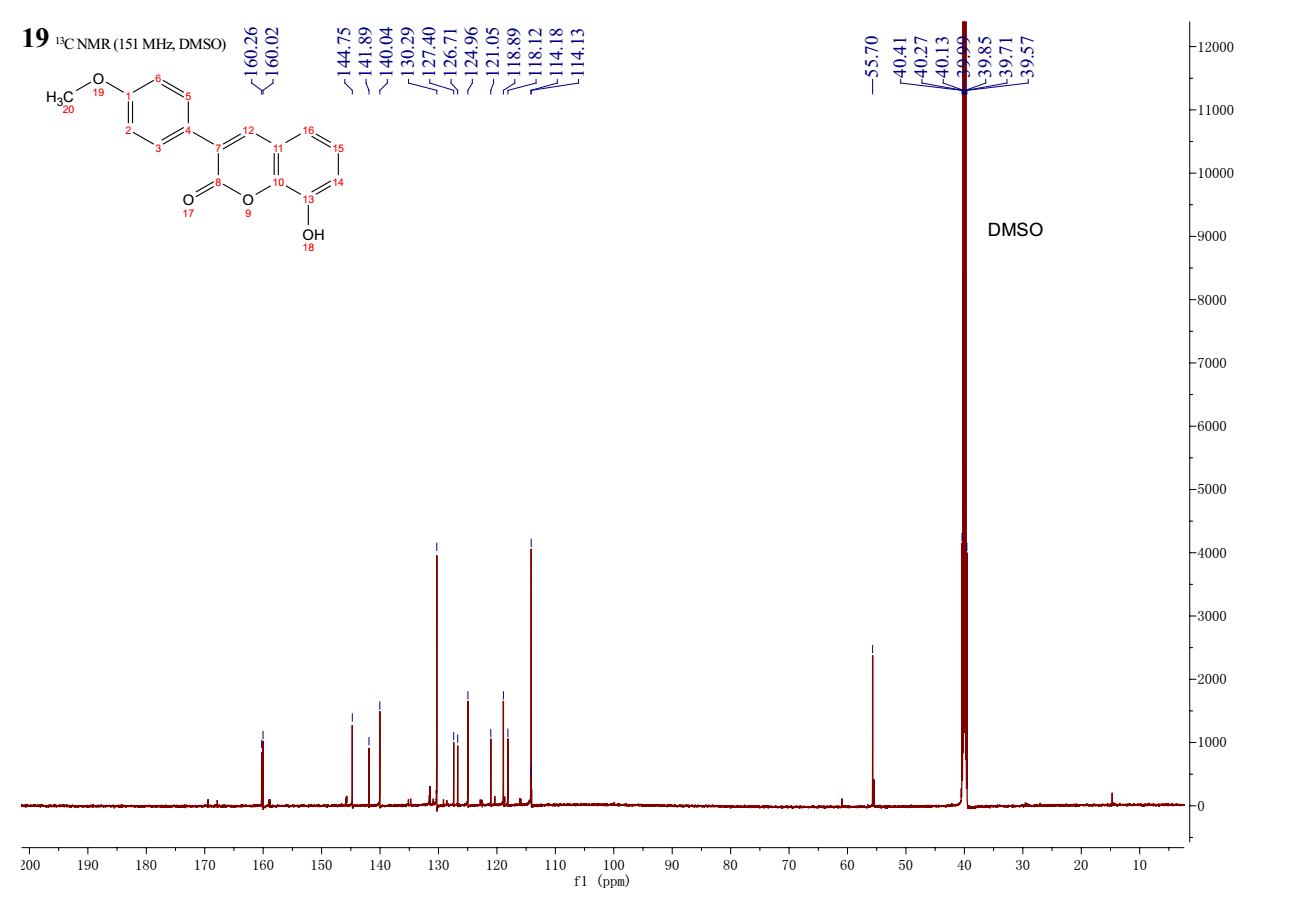


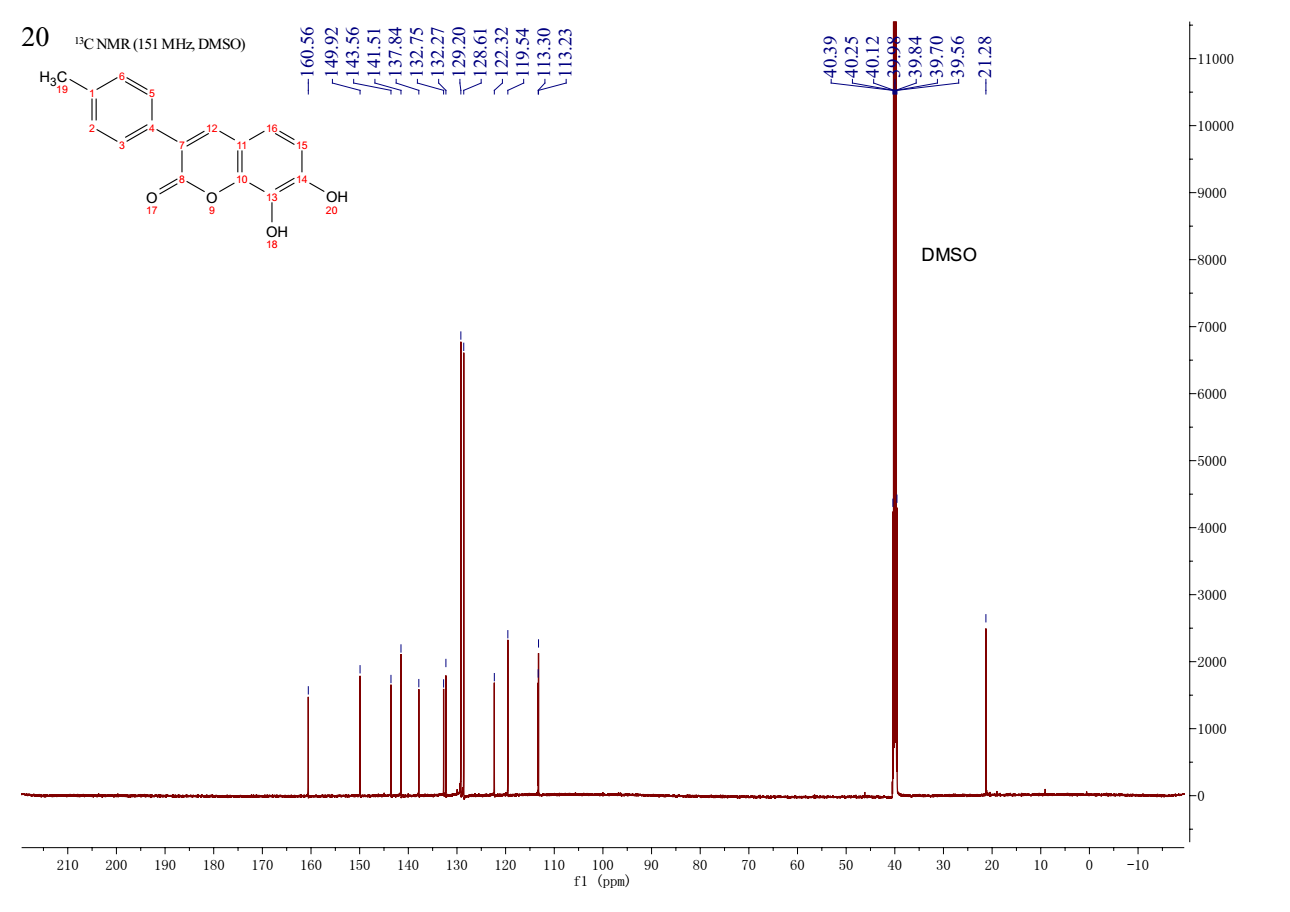


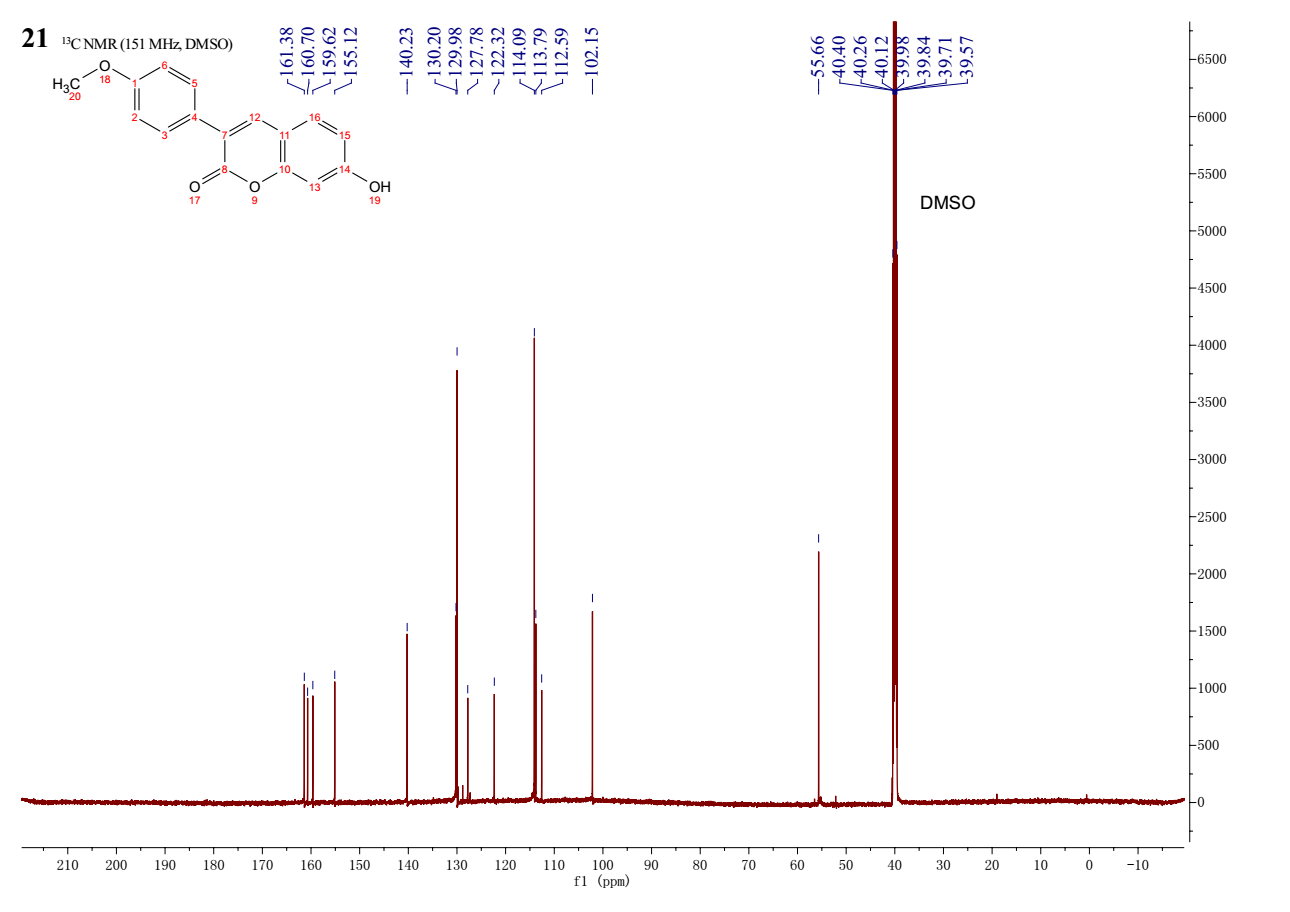


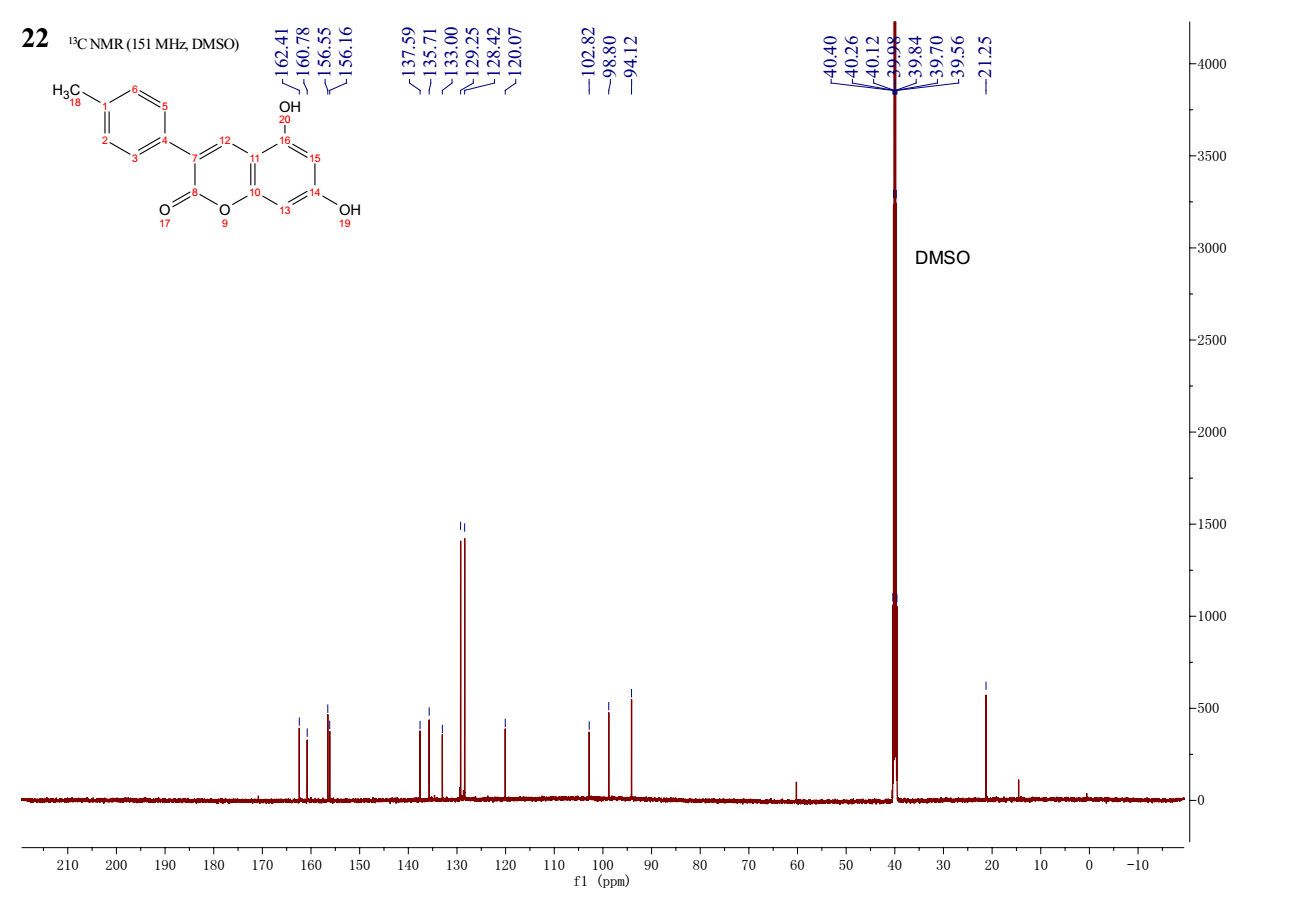


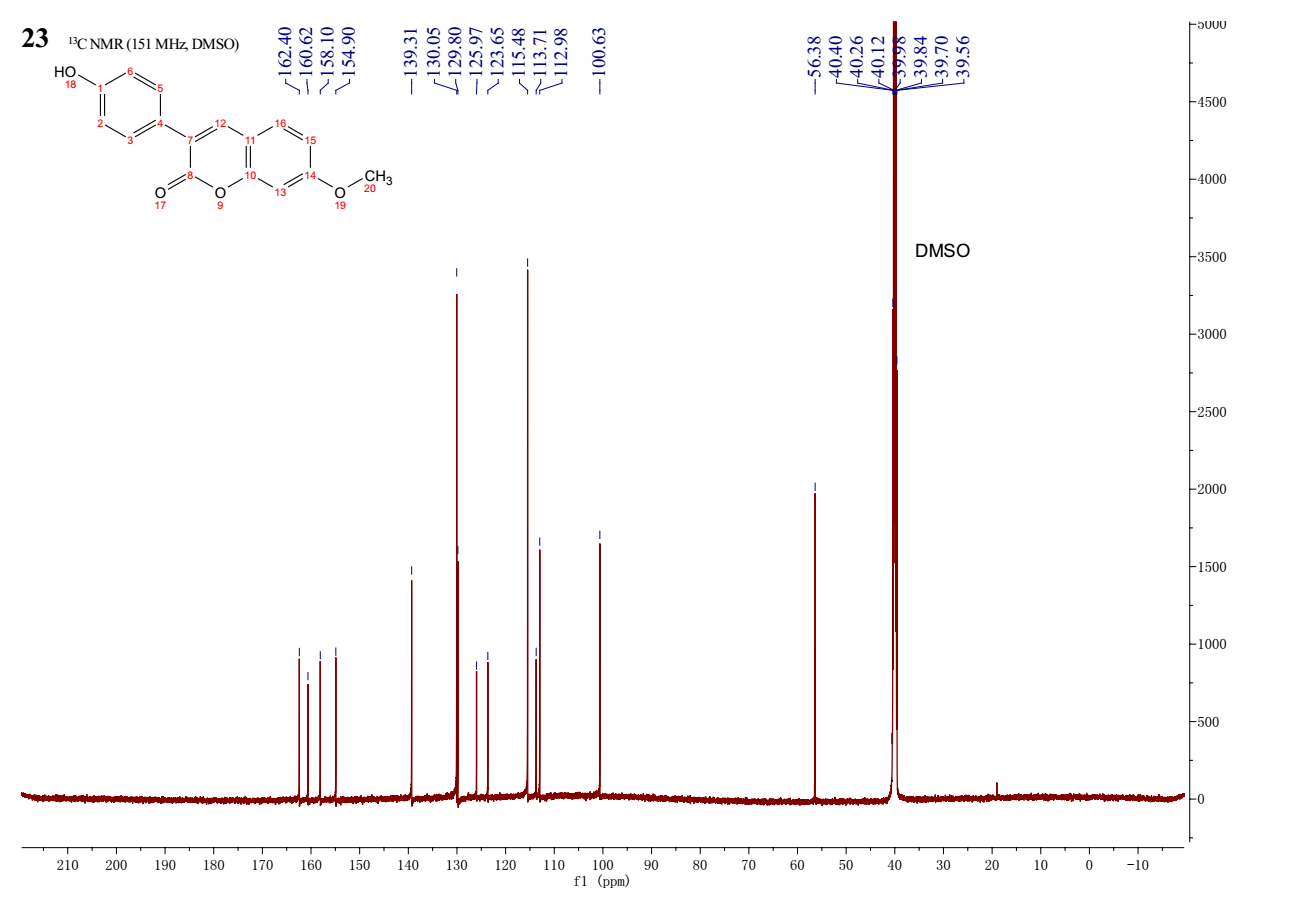


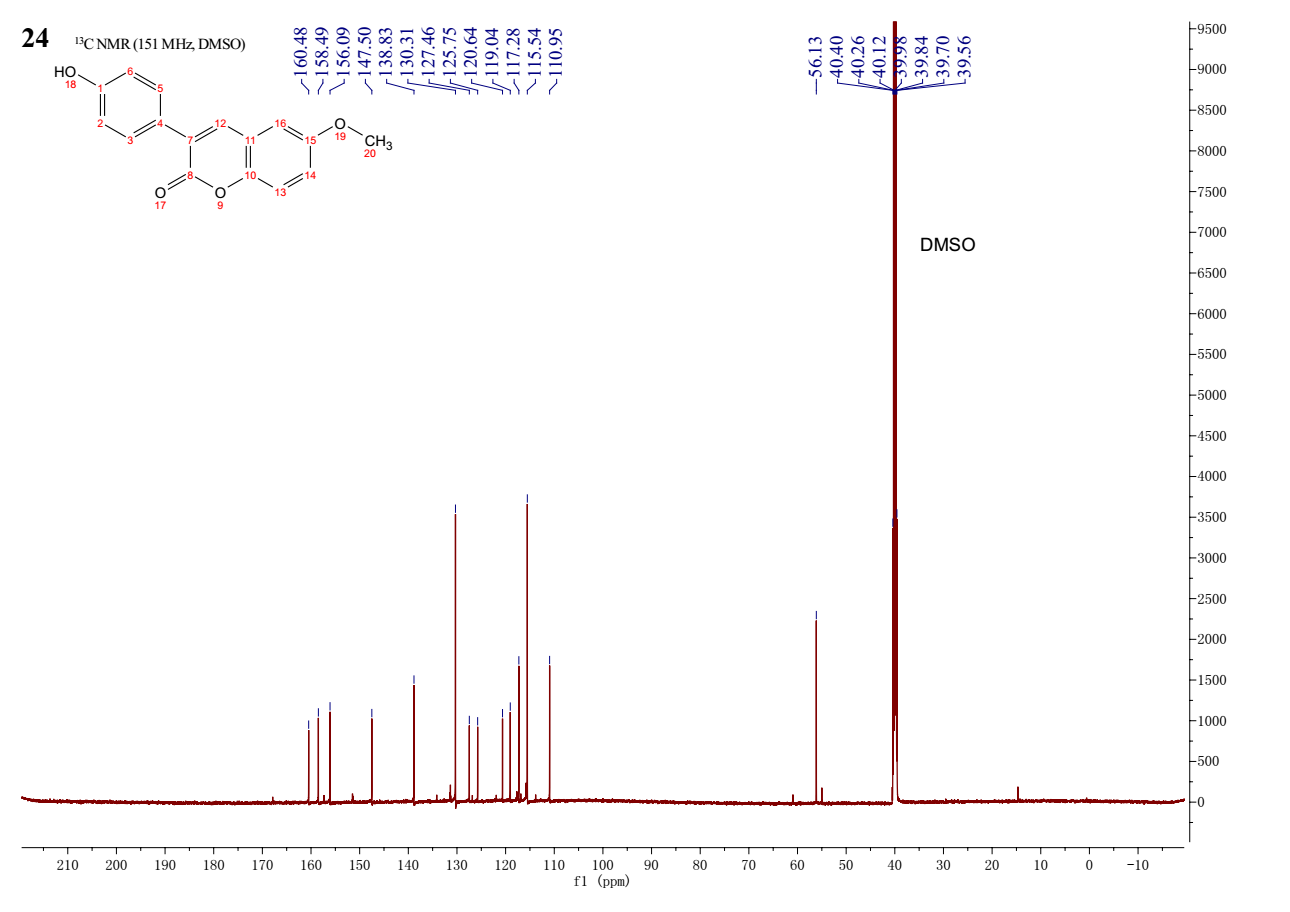


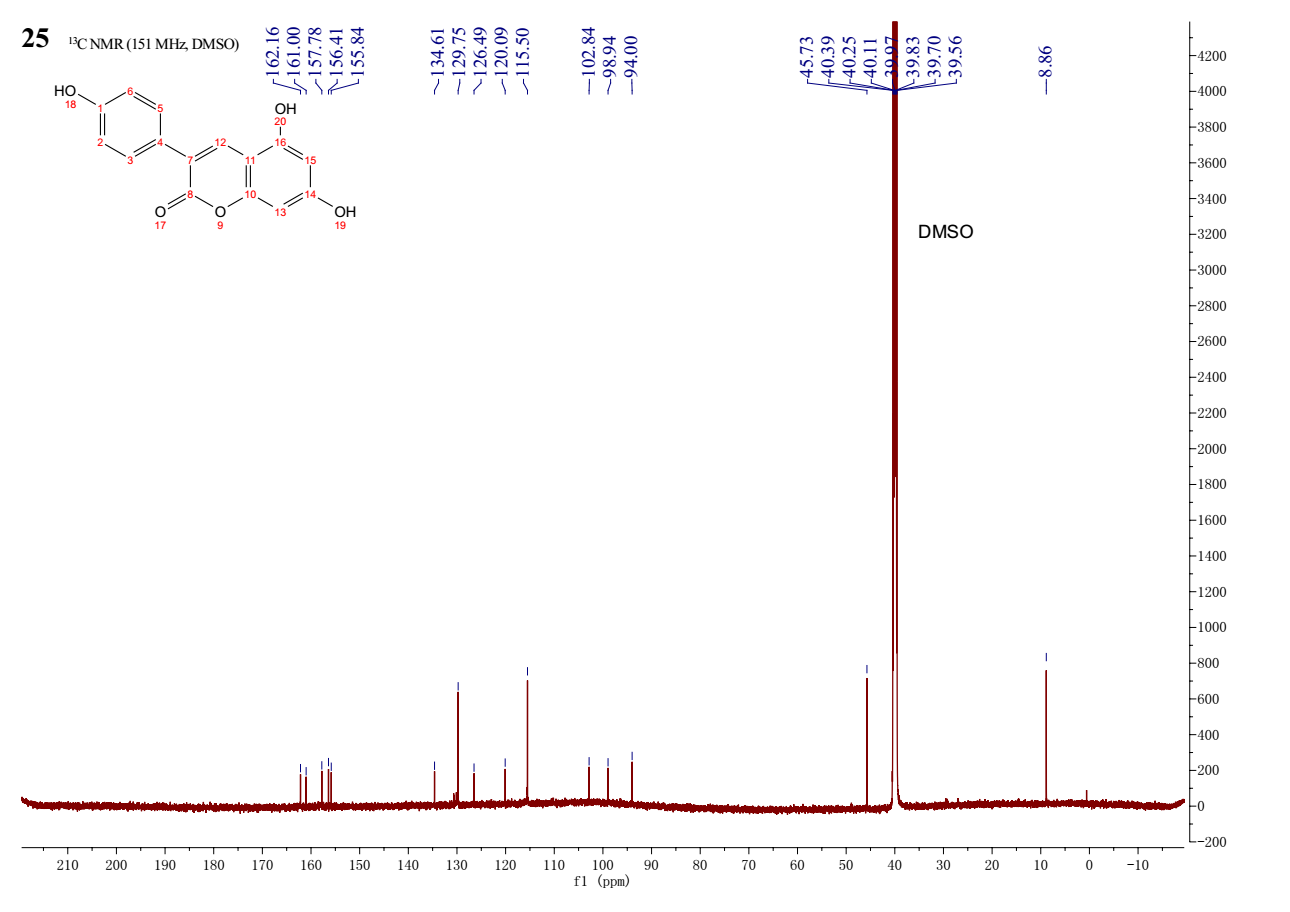


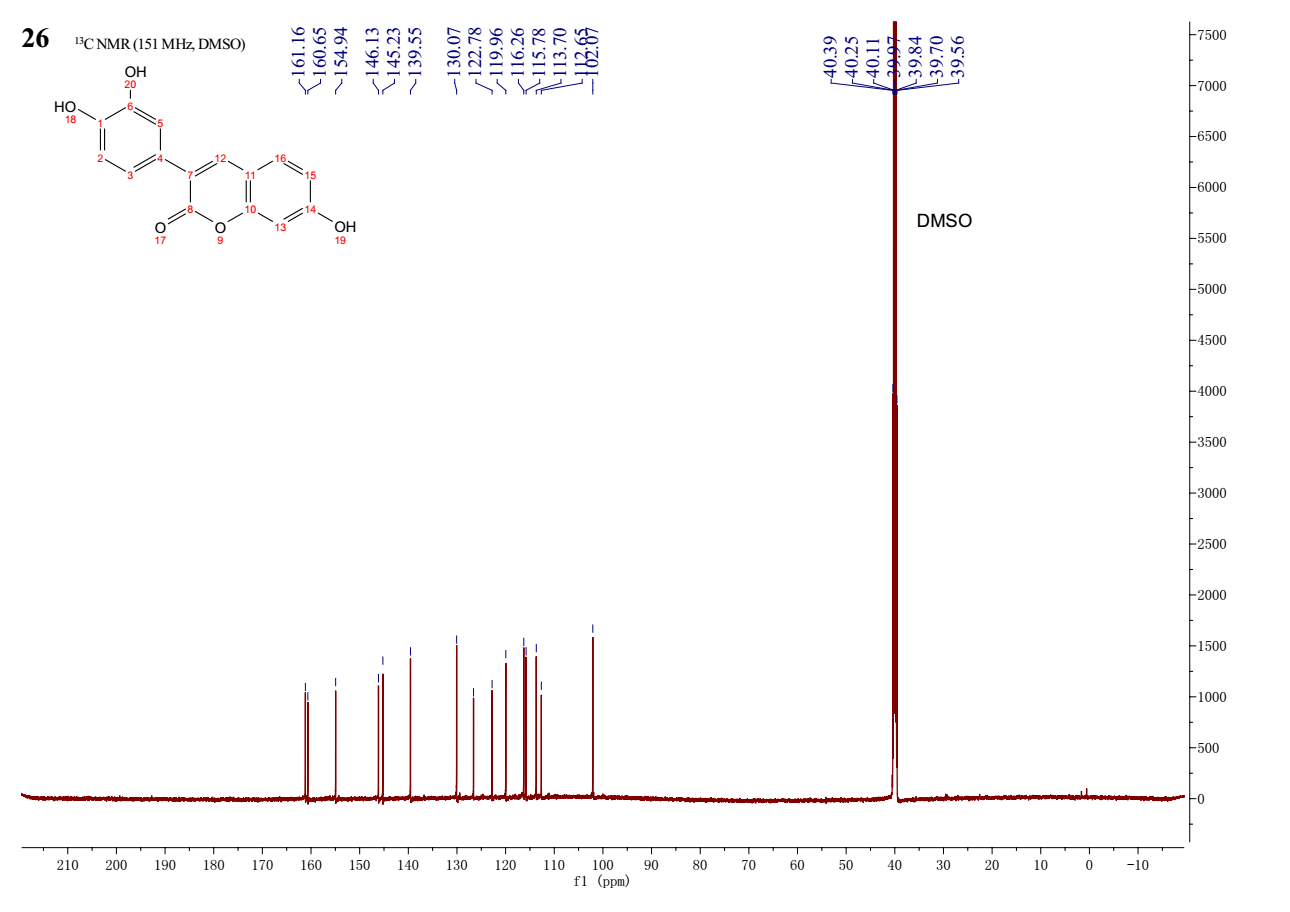


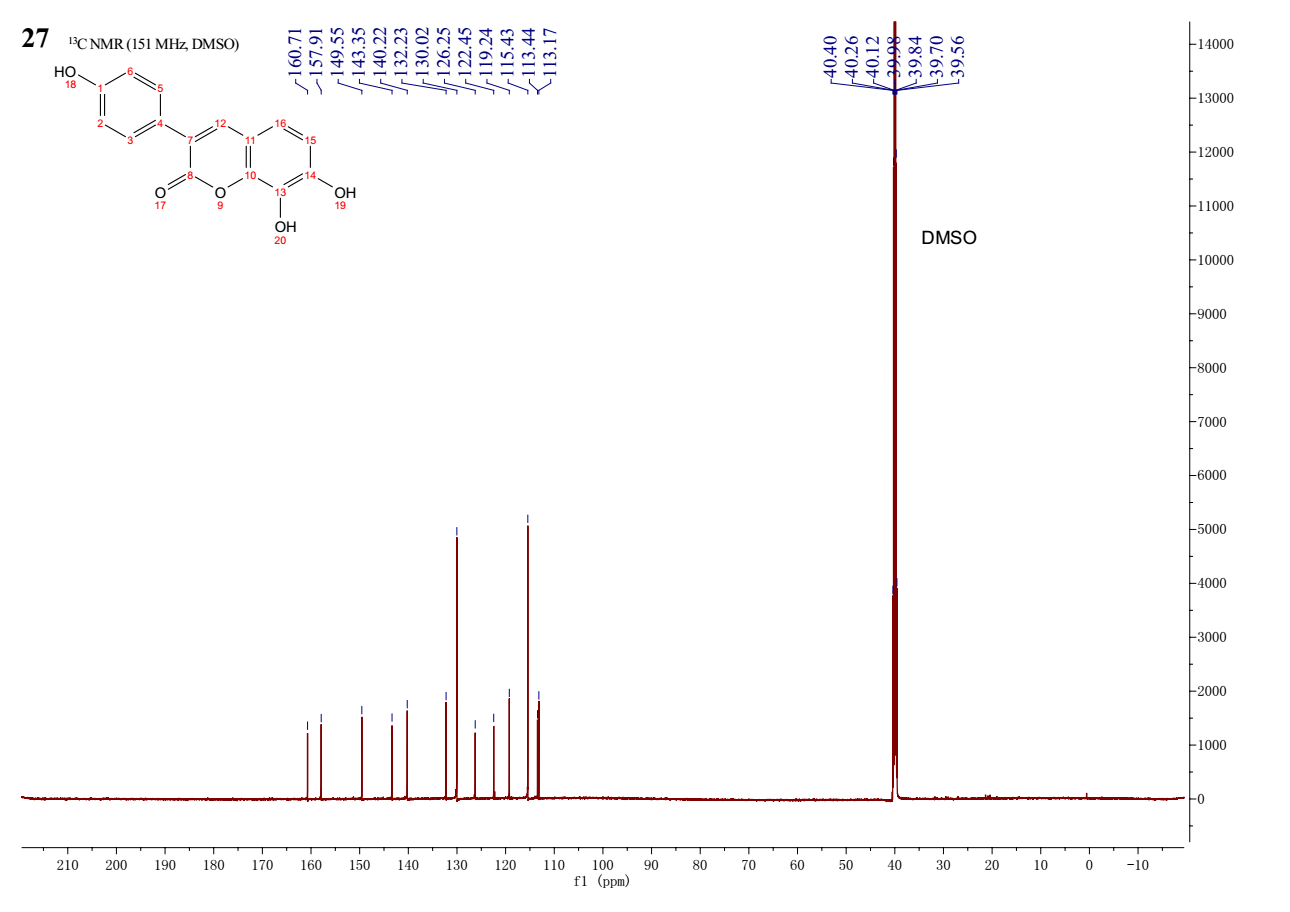


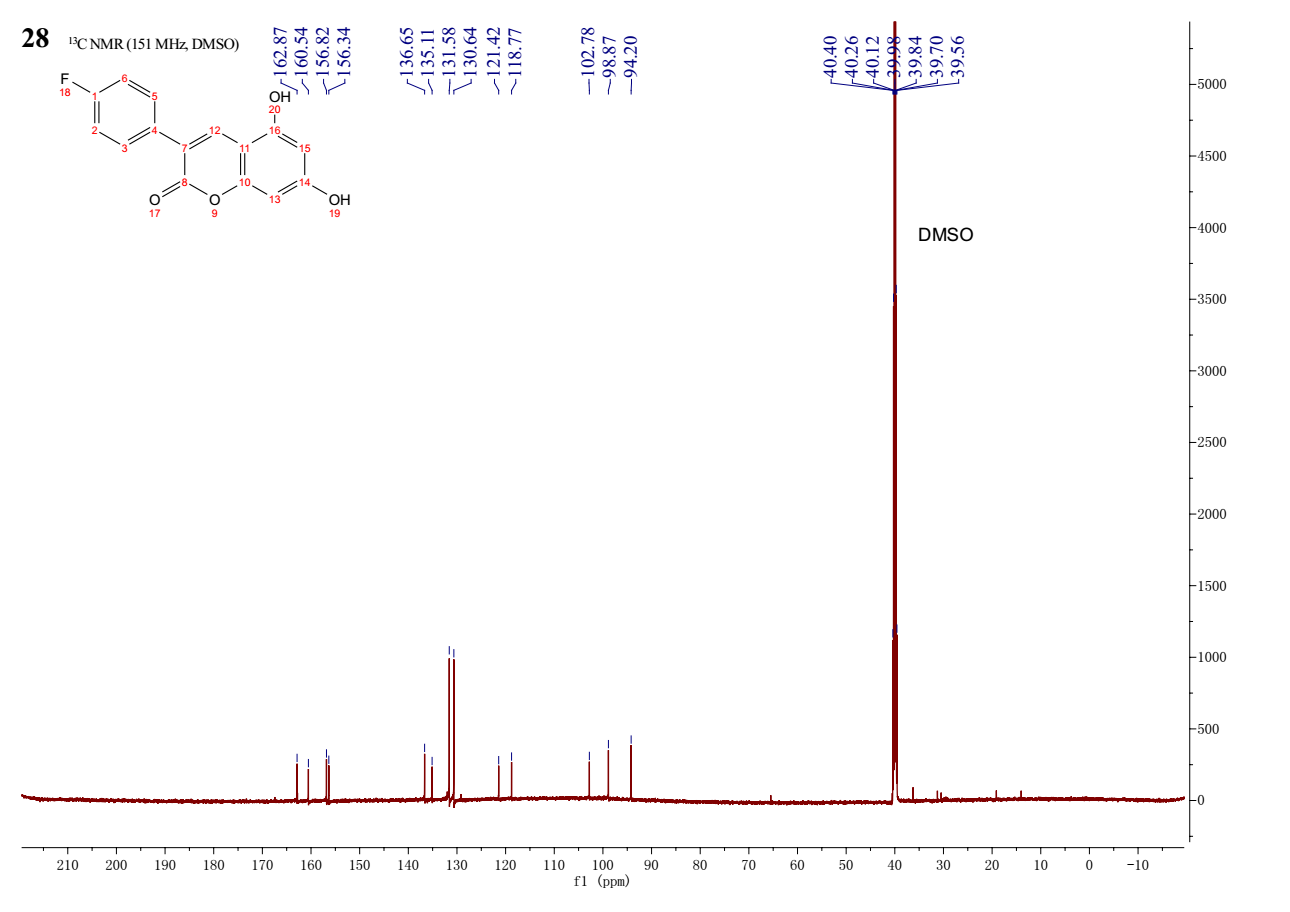


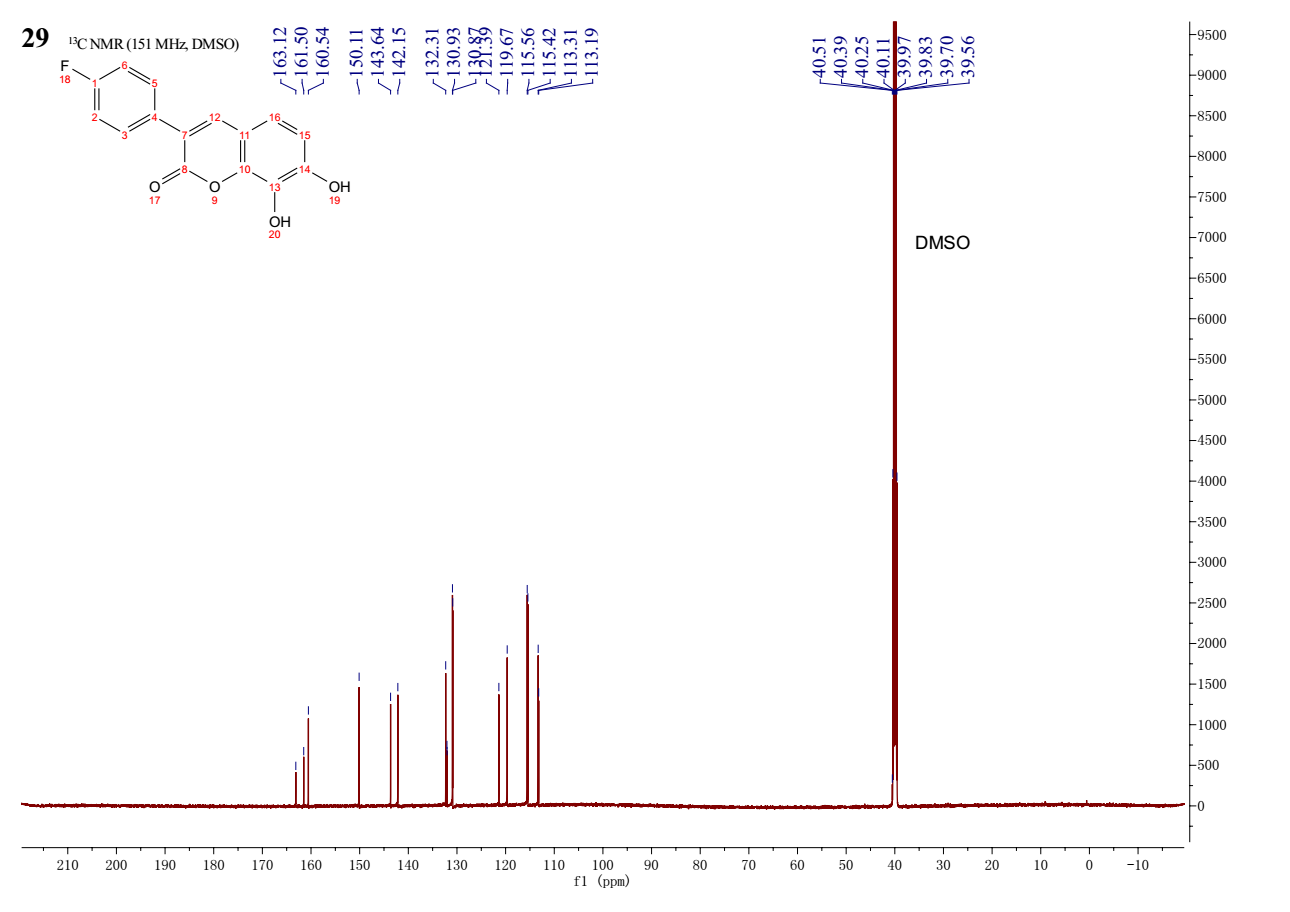


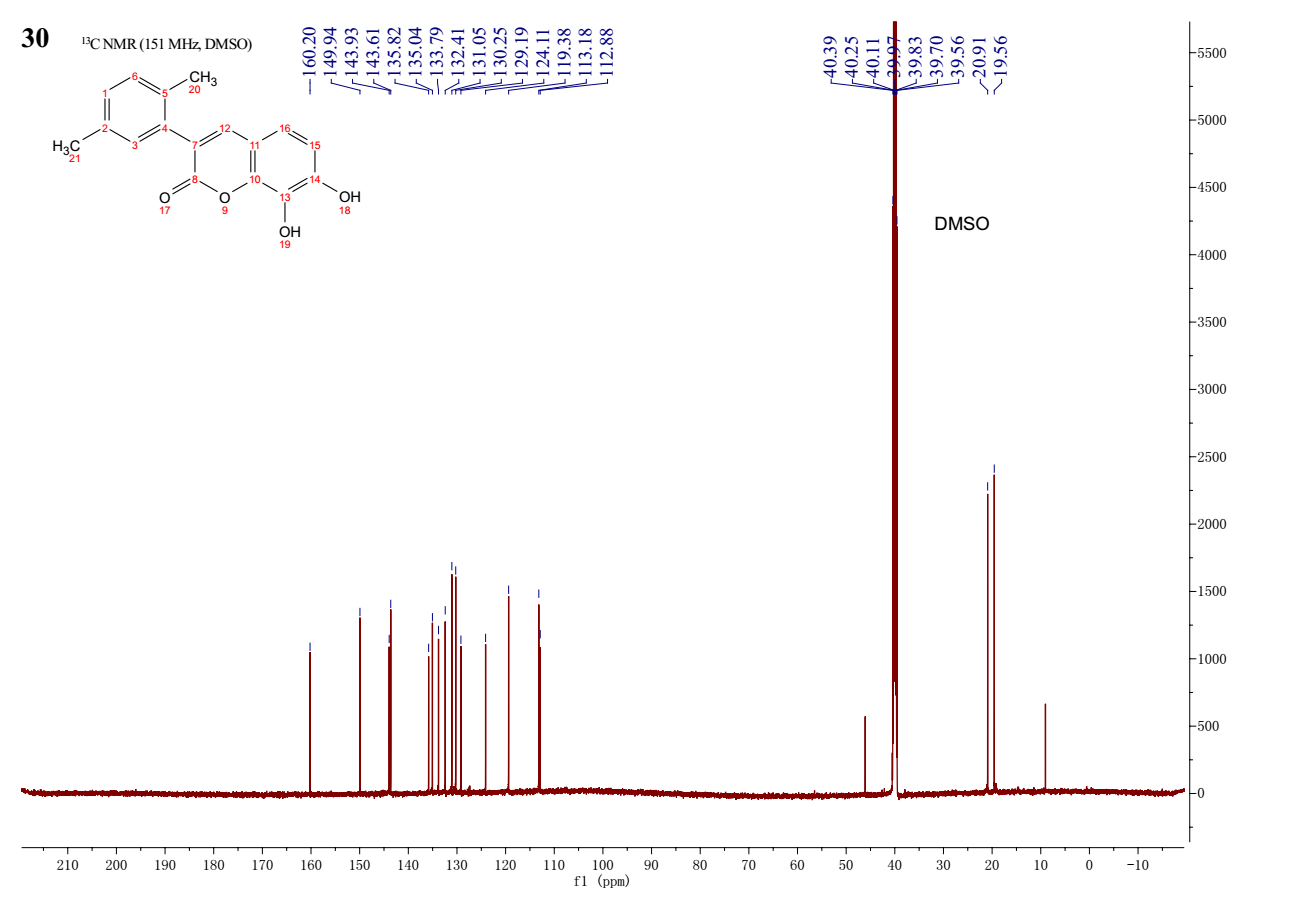

Supplement: IENZ_1518958_Supplementary Material [file IENZ_A_1518958_SM6529.docx]
